# A catalog of transcriptome genetic regulation in a consanguineous Middle Eastern biobank

**DOI:** 10.1038/s41588-026-02785-7

**Published:** 2026-11-02

**Authors:** Haroon Naeem, Ilhame Diboun, Zenab Siddig, Mohammadmersad Ghorbani, Sondoss Hassan, Radi Farhad, Shabir Moosa, Rawan Abouelhassan, Melissa Tauro, Rozaimi Mohamad Razali, Mohammed Iqbal, Elbay Aliyev, Fazulur Rehaman Vempalli, Muna Al Hashmi, Alia Al Massih, Rebecca Mathew, Valentina Mattei, Nicola James, Lisa Sara Mathew, Lee Silcock, Kun Wang, Davide Bedognetti, Sara Tomei, Damien Chaussabel, Khalid A. Fakhro, Stephen B. Montgomery, Younes Mokrab

**Affiliations:** 1 Sidra Medicine, Doha, Qatar; 2 Department of Biomedical Science, College of Health Sciences, Qatar University, Doha, Qatar; 3 Microbiology Unit, IRCCS Azienda Ospedaliero-Universitaria di Bologna, Bologna, Italy; 4 Department of Internal Medicine, University of Genova, Genova, Italy; 5 The Jackson Laboratory for Genomic Medicine, Farmington, CT USA; 6 Department of Genetic Medicine, Weill Cornell Medicine, Doha, Qatar; 7 College of Health and Life Sciences, Hamad Bin Khalifa University, Doha, Qatar; 8 Department of Pathology, Stanford University, Stanford, CA USA; 9 Department of Genetics, Stanford University, Stanford, CA USA; 10 Department of Biomedical Data Science, Stanford University, Stanford, CA USA

**Keywords:** Gene regulation, Genomics

## Abstract

Ancestry-specific genetic mapping of transcriptome regulation is pivotal for understanding genome function, especially in underrepresented consanguineous populations. Here we characterized 1,882 Qatar Biobank participants, integrating blood RNA sequencing, whole-genome sequencing and phenotypic data across 45 clinically relevant traits. We cataloged ~7 million significant quantitative trait loci (QTLs), both short and structural variants, impacting gene expression, isoform usage, splicing and allele-specific expression, uncovering 1,379 novel eGenes. Fine-mapping resolved 29,953 high-confidence, independent causal loci including 24% previously unreported as well as those with effect alleles showing significant divergence across global populations, while 1,371 were absent from public databases. Leveraging elevated autozygosity, we found that causal variants are depleted from runs of homozygosity, consistent with purifying selection, while recent autozygosity increased the burden of homozygous regulatory variants. We identified QTLs with recessive deleterious effects on 10 Mendelian disease genes and homozygous QTLs causing extreme outlier expression in 66 genes including dosage-sensitive ones. Colocalization analysis revealed 58 causal variants acting on 20 clinically relevant phenotypes, implicating novel genes including *ATP6V0B* and *RQCD1* in metabolic and blood disorders. These findings establish this QTL catalog as a valuable Middle Eastern resource for genome interpretation and genomic medicine.

## Abstract (web summary)

This study reports an atlas of transcriptomic genetic effects from a Middle Eastern population biobank, revealing population-specific regulatory variants and links to clinically relevant traits.

## Main

Genome-wide association studies (GWAS) have enabled the systematic mapping of a vast landscape of genetic variation to biological traits and diseases and are poised to scale up dramatically with fast-decreasing sequencing costs^1–3^. However, interpreting the functional relevance of this variation remains a major bottleneck, as ~90% of GWAS loci reside in noncoding regions, largely involved in gene regulation^4,5^. Large-scale mapping of molecular quantitative traits has therefore become central to elucidating regulatory mechanisms^6–8^. Advances in RNA sequencing (RNA-seq) now enable high-resolution characterization of multiple layers of transcriptional regulation, including gene expression, isoform usage, alternative splicing and allele-specific expression^9–14^, while recent advances in fine-mapping improved the identification of causal variants^15,16^.

Previous studies have characterized genetic regulation of gene expression in whole blood in various populations, including Sardinians^17^, English^18^, other Europeans (as part of GTEx^19^ and eQTLGen^20^), Puerto Ricans, Mexicans and African Americans (GALA II/SAGE)^21^, in addition to cell-line studies from 1000 Genomes Project (1KG) populations^22^. These studies showed substantial population differences in transcriptional regulation, particularly for immune-related processes^17,23–31^. Genetic ancestry also influences disease susceptibility through population-specific regulatory variation. For example, population-specific expression quantitative trait loci (eQTLs) have been identified in Korean patients with Crohn’s disease^32^, while ancestry-specific heritability and eQTLs have been reported among African Americans, Puerto Ricans and Mexican Americans^21^. Differences in allele frequencies shaped by evolutionary history further contribute to population-specific QTL effects^23,24,26^.

Despite these advances, transcriptome studies remain limited in underrepresented populations, particularly in the Middle East^33^. Owing to its unique demographic history, tribal structure and high level of consanguinity, Qatar provides a valuable model for studying Arab populations^34–36^. Qatar Biobank (QBB)^37^ collected a large phenotyped cohort from the local population in Qatar, a subset of which was characterized by whole-genome sequencing (WGS) through the Qatar Genome Program (QGP)^38^, offering a valuable resource spanning various Middle Eastern ancestry groups^36,39,40^.

**Extended Data Fig. 1:**
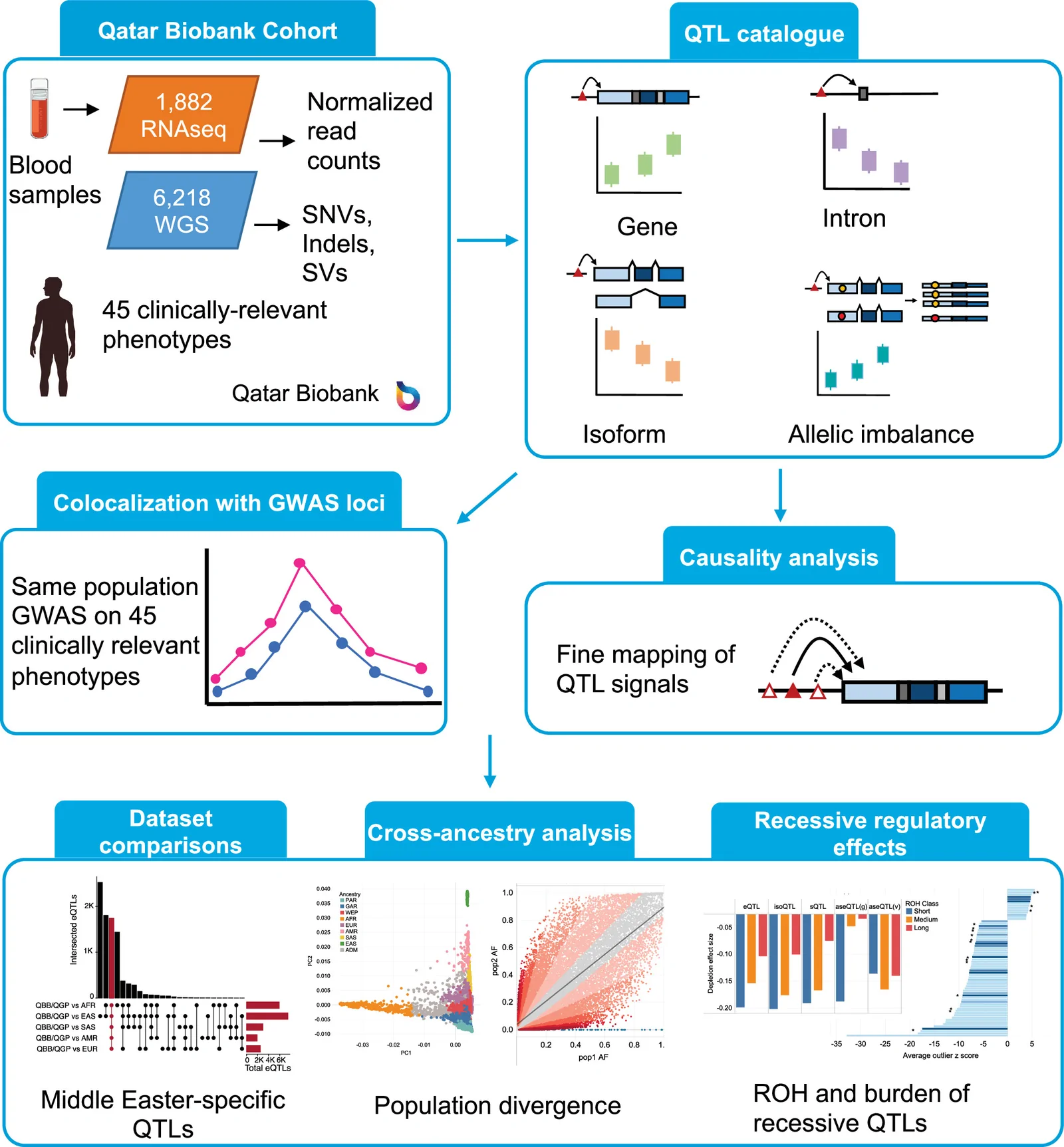
Establishing a catalogue of transcriptome genetic effects from a consanguineous Middle Eastern Biobank. Establishing an atlas of transcriptomic genetic effects from the Middle Eastern biobank of Qatar, revealing ancestry-specific causal variants, recessive regulatory mechanisms and links to clinically-relevant traits. The blood tube and human silhouette icons were obtained from NIAID Visual & Medical Arts, NIAID NIH BIOART Source: Cryo Blood Vial (https://bioart.niaid.nih.gov/bioart/87) and Human Male Outline Type VI (https://bioart.niaid.nih.gov/bioart/238), respectively.

In this Article, we characterized 1,882 predominantly healthy QBB participants by integrating whole-blood RNA-seq, DNA sequencing (DNA-seq) and phenotypic data on 45 clinically relevant traits (Extended Data Fig. 1). We generated a catalog of *cis*-acting QTLs comprising single-nucleotide variants (SNVs), short insertions/deletions (indels) and structural variants (SVs), affecting gene expression (eQTLs), splicing (sQTLs), isoform usage (isoQTLs) and allele-specific expression QTLs (aseQTLs). This is followed by fine-mapping of causal loci and analysis of population divergence, autozygosity and recessive regulatory effects in addition to colocalization with GWAS signals on clinical phenotypes from the same cohort. To our knowledge, this study represents the largest characterization of the Middle Eastern transcriptome published so far, providing a valuable resource for genome interpretation and genomic medicine.

## Results

### Study cohort and data generation

**Fig. 1:**
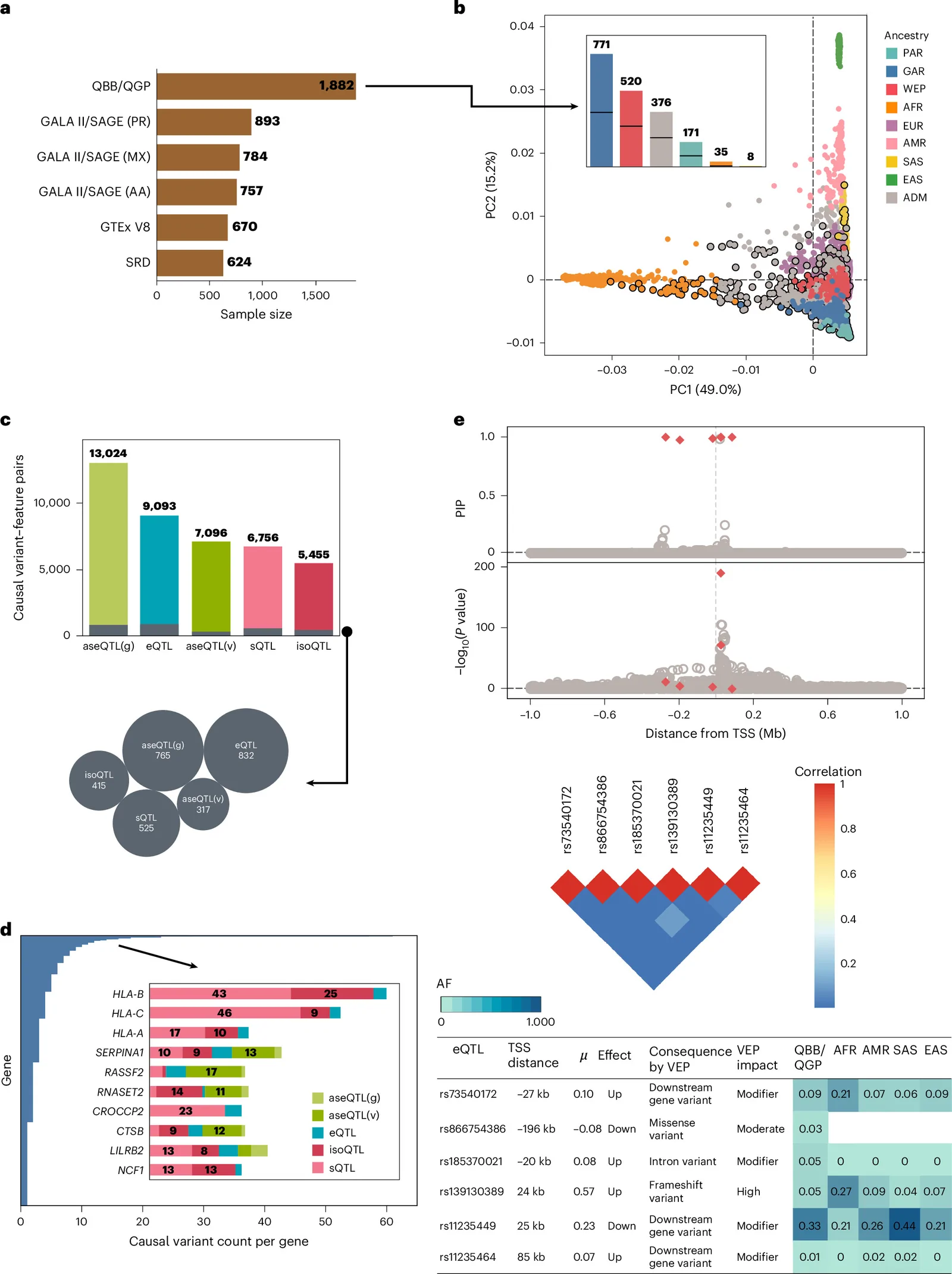
Landscape of causal QTLs in QBB. **a**, Sample sizes of the QBB/QGP study and previously published population studies of whole-blood QTLs. **b**, Principal component (PC) analysis plot showing QBB/QGP samples (highlighted dots) in the context of other QBB samples and 1KG continental populations (normal dots) (*n* = 8,720). The percentages shown on each axis indicate the proportion of total genetic variance explained by the corresponding PC. Inset: distribution of QBB/QGP samples across Arab Middle Eastern sub-ancestries. Horizontal lines indicate male and female counts. **c**, Number of significant causal variant–feature pairs identified per QTL type. The accompanying gray bubble plot (bottom) shows the number of pairs that were not significant in the univariate analysis. **d**, Multicausal variant effects. Plotted is the number of causal variants per gene across various QTL types. The inset highlights the top ten genes with the highest total QTL counts. Numbers within selected bars indicate counts per QTL type. **e**, The *FOLR3* gene is regulated by six independent fine-mapped eQTLs. Top: plot of PIP and −log_10_(*P* value) versus distance from the TSS for all tested eQTLs ±1 Mb. Causal eQTLs (PIP > 0.95) are highlighted in red. Middle: Heat map of pairwise LD (*R*^2^) among the six causal eQTLs. Bottom: attributes of the eQTLs, namely, reference SNP identifiers (rsIDs), TSS distance, effect size (*μ*), VEP annotation and allele frequencies in QGP/QBB and global populations (higher values shaded in darker green). PR, Puerto Ricans; MX, Mexican Americans; AA, African Americans; AFR, Africans; EUR, Europeans; AMR, Americans, SAS, South Asians; EAS, East Asians; ADM, admixed Arabs. Source data

We used a QBB/QGP cohort comprising 6,218 largely healthy individuals from Qatar’s local population, whole-genome sequenced and phenotyped for 45 clinically relevant traits (Supplementary Table 1). High-quality variant calls for SNVs, indels and SVs were obtained as described in Methods (Supplementary Table 2). For a subset, we generated peripheral blood RNA-seq, and after processing and quality control (QC), we retained 1,882 high-quality samples for which we quantified gene, isoform, splicing and allelic expression (Methods and Supplementary Figs. 1 and 2). This cohort had a near-equal male-to-female ratio (1.03), representing diverse Arab Middle Eastern ancestries (Fig. 1a,b), closely reflecting the broader QBB/QGP cohort whose genetic structure we had characterized^36^.

### Landscape of QTLs

**Extended Data Fig. 2:**
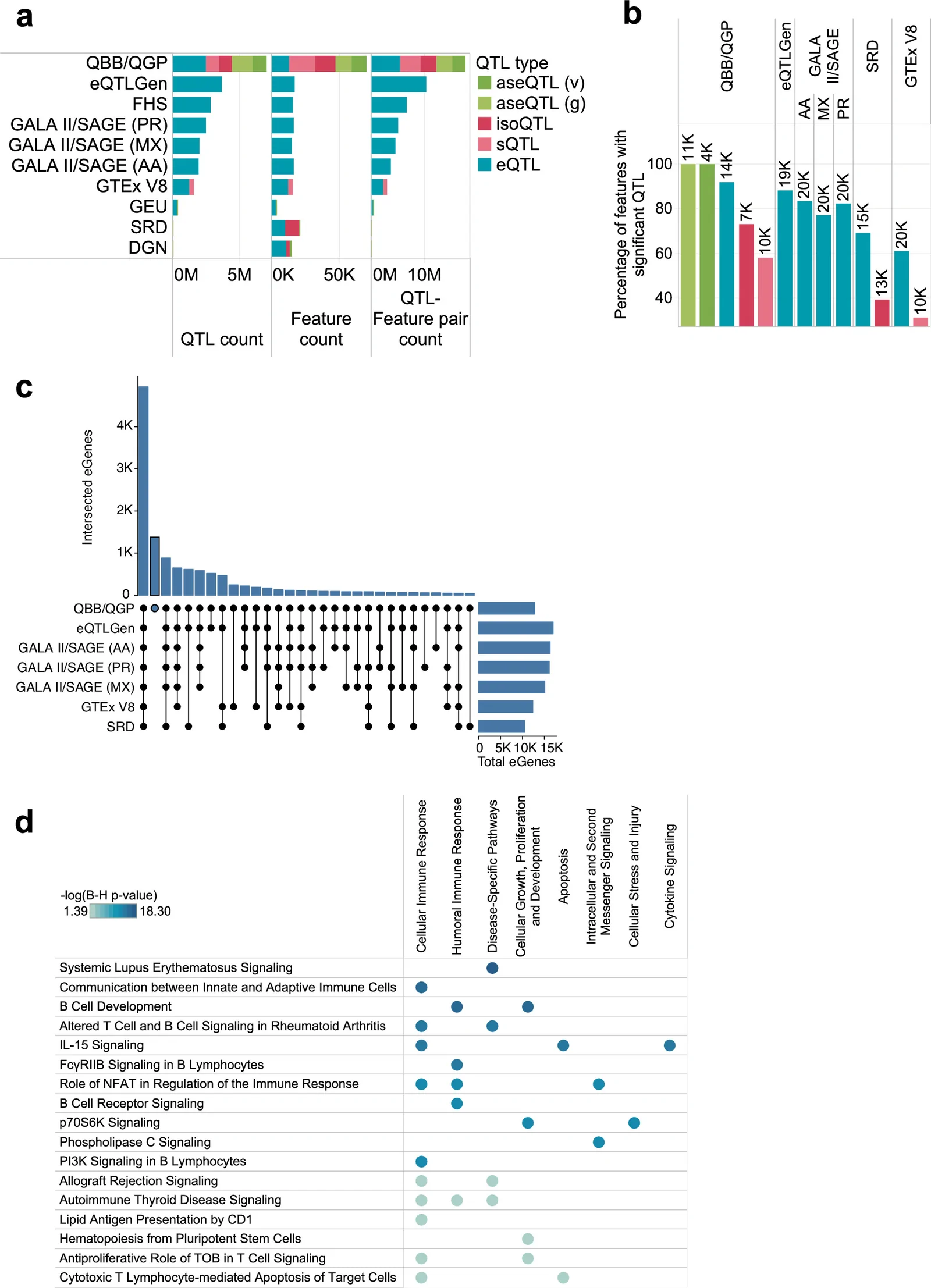
Univariate Quantitative Trait Loci in QBB/QGP. **(a)** Number of significant univariate QTLs, transcriptome features, and QTL-feature pairs identified per QTL type in QBB/QGP and other published studies (FDR < 0.05). **(b)** Proportion of transcriptomic features with at least one significant QTL, relative to the total number tested, across QBB/QGP and other studies. Absolute counts are shown atop each bar. **(c)** UpSet plot showing overlap of eGenes identified in QBB/QGP and other public datasets. Intersections are color-coded by count intensity. **(d)** Significantly-enriched functional and disease pathways associated with eGenes reported only in QBB/QGP (n = 1,379). Rows represent pathways, while columns represent IPA-defined functional categories. Dots are coloured by -log(BH-adjusted p-value) derived from Fisher’s exact test (IPA). Public studies are labelled as DGN: Depression Genes and Networks dataset; eQTLGen: eQTL Consortium dataset; FHS: Framingham Heart Study (FHS). GALA II/SAGE (PR), GALA II/SAGE (MX) and GALA II/SAGE (AA): Puerto Ricans, Mexican Americans and African Americans datasets from the GALA II/SAGE study, respectively; GEU: GEUVADIS dataset; GTEx V8: Genotype to tissue expression consortium dataset; SRD: Sardinia dataset. Populations are labelled as ADM: Admixed Arabs; AFR: Africans; AMR: Americans; EAS: East Asians; EUR: Europeans; GAR: General Arabs; PAR: Peninsular Arabs; SAS: South Asians; WEP: West Eurasian and Persian Arabs. Source data

We tested the association between SNVs, indels, and SVs and transcriptomic traits using univariate regression (Supplementary Fig. 5 and Methods), resulting in a comprehensive catalog of *cis*-acting eQTLs, isoQTLs, sQTLs, aseQTL (variant (v) level) and aseQTL (gene (g) level). Collectively, this comprised 17.9 million significant QTL–feature associations, involving 7.0 million unique QTLs and 69,900 unique features (false discovery rate (FDR) < 0.05), exceeding the scale and diversity of several public datasets (Extended Data Fig. 2a).

QTLs were predominantly enriched near transcription start sites (TSSs), with >80% located within 0.5 megabase (Mb). aseQTLs were closest to TSSs on average, followed by isoQTLs, sQTLs and eQTLs (Supplementary Fig. 7a,b). All QTL classes were enriched for functional annotations based on the variant effect predictor (VEP)^41^ (Supplementary Fig. 7c) and depleted in intergenic regions, while sQTLs were additionally depleted in transcription factor binding sites. Most QTLs (54–72%) were associated with a single gene (Supplementary Fig. 8), and most associated genes were protein coding (Supplementary Fig. 9).

A high proportion of tested transcriptomic features had significant QTLs (FDR < 0.05) (Extended Data Fig. 2b)—99.8% for aseQTLs, 91.9% for eQTLs, 73.0% for isoQTLs and 57.9% for sQTLs. These proportions exceeded those in whole-blood QTL studies of eQTLGen, GALA II/SAGE, the Sardinia dataset and GTEx, probably reflecting the quality and characteristics of our dataset^42^. Relative to those resources, QBB/QGP added 11% of eGenes, 30% of eQTLs and 52% of eQTL–gene pairs (Extended Data Fig. 2c and Supplementary Fig. 10a), with higher proportions for other QTL classes (Supplementary Fig. 10b). The 1,379 eGenes detected only in QBB/QGP were enriched for immune and cellular pathways, including signaling in systemic lupus erythematosus (Benjamini–Hochberg (BH) *P* value = 10^−18^), communication between innate and adaptive immune cells (BH *P* value = 10^−15^) and B-cell development (BH *P* value = 10^−15^) (Extended Data Fig. 2d).

### Fine-mapping of QTLs identifies causal variants

We fine-mapped *cis*-QTLs using a sum of single effects (SuSiE)-based workflow^16^ with individual-level genotypes and stringent criteria (Methods and Supplementary Fig. 11). Effect sizes (*μ*), posterior inclusion probability (PIP) and log Bayes factor (LBF) in addition to slope (*β*) versus MAF showed expected distributions (Supplementary Fig. 12). We identified 40,735 high-confidence fine-mapped causal variant–feature pairs (PIP > 0.95 and LBF > 2), involving 29,953 distinct variants, 24,083 transcriptomic features and 10,302 genes (Fig. 1c and Supplementary Fig. 13). aseQTL(g) accounted for the highest number of associations (*n* = 12,472), followed by eQTLs (*n* = 8,388), corresponding to 7,436 and 5,161 unique genes, respectively. In the univariate test, ~7% of fine-mapped pairs were not significant, consistent with previous SuSiE studies^15,16,43^, underscoring the value of fine-mapping to uncover cryptic regulatory effects (Fig. 1c).

**Extended Data Fig. 3:**
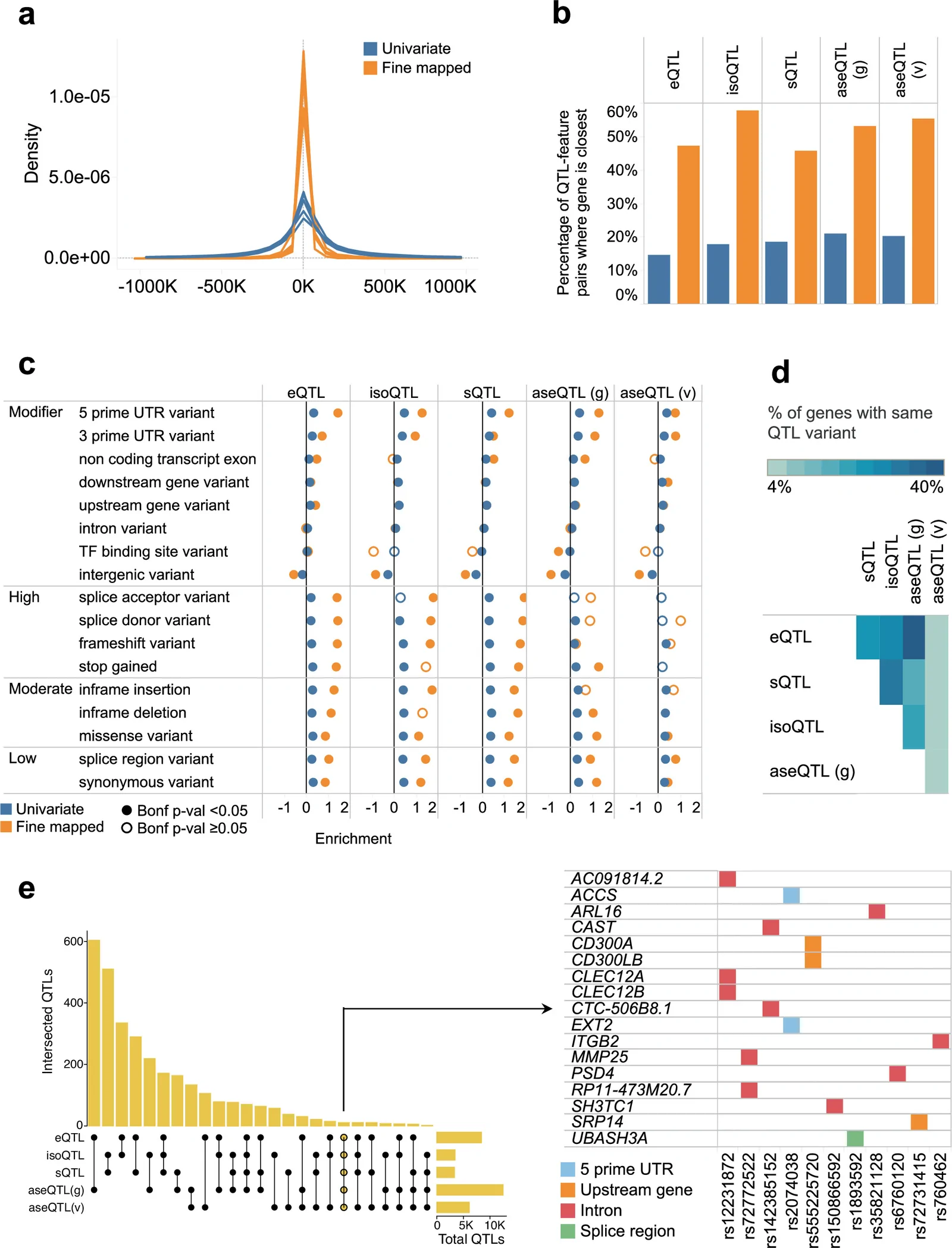
Genomic Patterns of Causal variants. **(a)** Density distribution of QTL–feature distances relative to the transcription start site (TSS), comparing univariate and fine-mapped QTLs. **(b)** Proportion of QTL–feature pairs where the gene is the closest to the variant, for both univariate and fine-mapped results. **(c)** Enrichment of univariate and causal variants across genomic annotations and impact predicted by VEP, calculated as the log ratio of observed to expected counts, both normalised for baseline variant count. Full circles denote statistically-significant enrichment ( > 0) or depletion ( < 0) at Bonferroni-adjusted p-value < 0.05, while open circles denote non-significant values. **(d)** Percentage of genes where a pair of QTL types have the same underlying causal variant. **(e)** Top: UpSet plot showing the overlap of variants acting as causal variants of different QTL types, irrespective of gene. Bottom: List of 11 variants identified as causal variants across all types, along with their affected genes and genomic annotations. Source data

Fine-mapping increased QTL density near TSSs compared with previous univariate QTLs (Extended Data Fig. 3a) and increased the proportion of QTL–gene pairs, where the gene was the closest from 15–21% to 46–58% (Extended Data Fig. 3b). VEP enrichment patterns resembled those from univariate mapping but were generally stronger, supporting previous reports^44^ (Extended Data Fig. 3c). Enrichment was observed for synonymous variants, underscoring their regulatory relevance^45,46^. Compared with public whole-blood datasets, 3.2% of eGenes, 26.4% of eQTLs and 38.7 % of eQTL–gene pairs were unique to QBB/QGP, with higher proportions for other QTL types (Supplementary Figs. 14 and 15). Overall, 6.2% of genes, 24.4% of QTLs and 47.1% of QTL–gene pairs were only reported in QBB/QGP (Supplementary Fig. 16).

### Multivariant and multigene effects of causal variants

Most fine-mapped QTLs regulated a single gene (52–76%), with progressively fewer affecting multiple genes (Supplementary Fig. 17a). Similar trends occurred in public datasets, although QBB/QGP contained more multigene QTLs (Supplementary Fig. 17b).

Most genes are influenced by few causal variants, but some by many (Fig. 1d), including *HLA-B*, *HLA-C*, *HLA-A* and clinically relevant genes such as *CTSB*, linked to dilated cardiomyopathy^47^ and immunosuppression in gliomas^48^. Among actionable genes from the American College of Medical Genetics, 36 harbored at least one causal variant (Supplementary Fig. 18). Only 17% were in ClinVar^49^, labeled as benign/likely benign or variant of uncertain significance. *GAA*, implicated in glycogen storage disease^50^, harbored the most fine-mapped QTLs (*n* = 31), 80% of which were previously unreported.

The folate receptor γ gene, *FOLR3*, carried the most fine-mapped eQTLs (*n* = 6) (Fig. 1e). Two of them (rs73540172 and rs11235449) were reported as univariate eQTLs in eQTLGen and GALA II/SAGE. rs139130389 shows the largest effect size (*μ* = 0.57), is predicted to cause a frameshift and is associated with the FGFR2/FOLR3 protein-level ratio^51^. Some of the variants are common in specific populations, including rs139130389 in Africans (allele frequency (AF) = 0.27) and rs11235449 in South Asians (AF = 0.44), whereas rs866754386 and rs185370021 are rare in QBB/QGP (AF = 0.03 and 0.05, respectively) and extremely rare globally. The eQTLs may affect folate uptake and metabolism, with implications to fetal development^52^.

For pairs of fine-mapped QTLs regulating the same gene, one underlying variant was shared in 4–37% of cases, depending on the QTL type (Extended Data Fig. 3d). Sharing was highest for eQTL–aseQTL(g) (37%) and isoQTL–sQTL (33%), reflecting related molecular traits. A total of 164 variants acted as both eQTL and aseQTL(g), while 510 acted as both isoQTL and sQTL. Eleven largely intronic variants acted as all QTL types across 17 genes (Extended Data Fig. 3e).

### Middle Eastern ancestry-specific QTLs

**Fig. 2:**
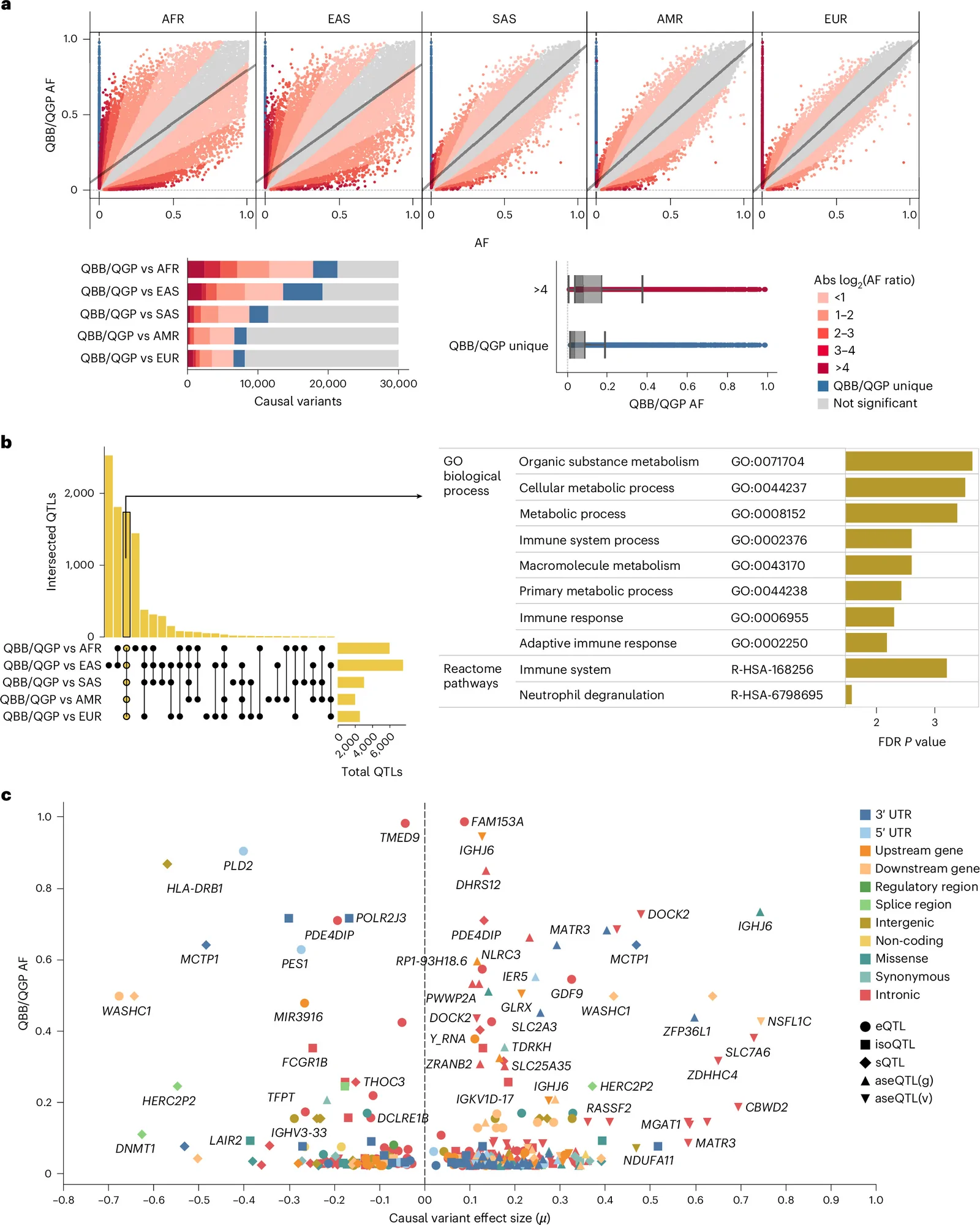
Divergence of QBB/QGP causal variants in global populations. **a**, Top: pairwise comparisons of allele frequencies between the QBB/QGP cohort and 1KG populations for the causal variants identified in this study. Each dot represents a single variant. Red and blue dots indicate variants with significantly different allele frequencies, while gray dots indicate those not significant, as assessed using a quasi-Poisson GLM (*P* < 0.05; Bonferroni corrected). Blue highlights QTLs reported only in QBB/QGP, while red marks those present in other populations, with gradient intensity reflecting the absolute (Abs) log_2_ of the AF ratio. Bottom left: number of causal variants per comparison. Bottom right*:* box plot of allele frequencies for QTLs with the largest significant differences (log_2_(AF ratio) > 4; *n* = 4,907) and for those exclusively present in QBB/QGP (*n* = 6,316). The center line indicates the median, the box limits indicate the first and third quartiles (Q1 and Q3), the whiskers extend to 1.5× the interquartile range and individual points represent QTL variants. **b**, Left: UpSet plots showing the overlap of causal variants with significant and large AF differences between QBB/QGP and each 1KG population (log_2_(AF ratio) > 4 as identified in **a**), and variants reported only in QBB/QGP. Right: biological pathways enriched (FDR < 0.05) for genes regulated by 267 SNV QTLs showing significant and large AF differences relative to all 1KG populations. GO, gene ontology. **c**, Scatter plot of the 267 causal SNVs described in **b**, illustrating the relationship between effect size (*μ*) and allele frequency in QBB/QGP. Different shapes represent different QTL types, and colors indicate VEP annotations. Gene names are labeled where space permits. Source data

Effect sizes of shared fine-mapped QTLs between QBB/QGP and GTEx V8 were highly concordant (*r* = 0.9; Supplementary Fig. 19a), indicating that common regulatory effects are broadly conserved across populations^22^, whereas ancestry specificity is more often driven by AF differences. We therefore compared QBB/QGP causal variant AF with 1KG populations^53^ using a generalized linear model (GLM) (Methods). We identified 26,907 variants with significant AF differences (Supplementary Fig. 19b,c). Divergence was greatest relative to Africans, followed by East Asians, South Asians, Americans and Europeans, with 8,200–21,400 significant variants per comparison (Fig. 2a). Of those, 9,021 had an absolute log_2_(AF ratio) > 4 and 6,316 were absent from 1KG, with median AFs of 0.08 and 0.04, respectively.

Among the 9,021 variants, 1,739 differed significantly in AF from every 1KG population or were absent from 1KG (Fig. 2b, left). These included 267 SNVs regulating 297 genes enriched for metabolic and immune pathways (Fig. 2b, right). The largest absolute effect sizes were observed for QTLs regulating *IGHJ6* (rs1950943, missense variant; *μ* = 0.74) and *WASHC1* (rs7043860, downstream gene variant; *μ* = –0.68), with AF of 0.74 and 0.50, respectively (Fig. 2c). *IGHJ6* encodes immunoglobulin heavy joining 6, while *WASHC1* encodes part of nucleation-promoting factor involved in major histocompatibility complex (MHC) and other protein trafficking^54^. A total of 13 variants are in ClinVar: 7 benign/likely benign, 5 with conflicting interpretation and 5 variants of uncertain significance (Supplementary Fig. 20).

We identified 1,301 fine-mapped QTLs whose variants are absent from public databases, including dbSNP, affecting 1,242 genes (Supplementary Fig. 21a). Their genomic distribution resembled that of other QTLs (Supplementary Fig. 21b). Across the subgroups of general Arabs (GAR), peninsular Arabs (PAR) and West Eurasian and Persian Arabs (WEP), 778 showed significant AF differences (Supplementary Fig. 21c), most prominently between PAR and WEP (42%). In total, 29 genes were affected by fine-mapped QTLs with absolute log_2_(AF ratio) > 4; the largest effect size was observed for an eQTL for *PIGV*, involved in glycosylphosphatidylinositol (GPI) anchor biosynthesis (eQTL, *μ* = –0.32), differing markedly between PAR and WEP (0.002 versus 0.068) (Supplementary Table 8).

### Landscape of structural variant QTLs

We tested 6,598 deletions (Del), 619 duplications (Dup) and 548 inversions (Inv) for *cis*-acting effects on gene expression, splicing and isoform usage (Methods). We identified 3,078 significant univariate SV QTLs (FDR < 0.05), representing 34–41% of the tested variants within each SV class; the larger number of Del QTLs reflects the larger number tested (Supplementary Fig. 22a). The SV QTLs regulated 5,307 genes and comprised 55% eQTLs, 23% isoQTLs and 22% sQTLs. Median sizes were 139 base pairs (bp) (Dup), 1.2 kilobases (kb) (Del) and 40.3 kb (Inv), with similar distributions across QTL types (Supplementary Fig. 22b). Most occurred in noncoding regions, while some spanned entire genes, untranslated regions (UTRs) and promoters and led to TSS/exon duplications and loss-of-function (Supplementary Fig. 22c).

**Extended Data Fig. 4:**
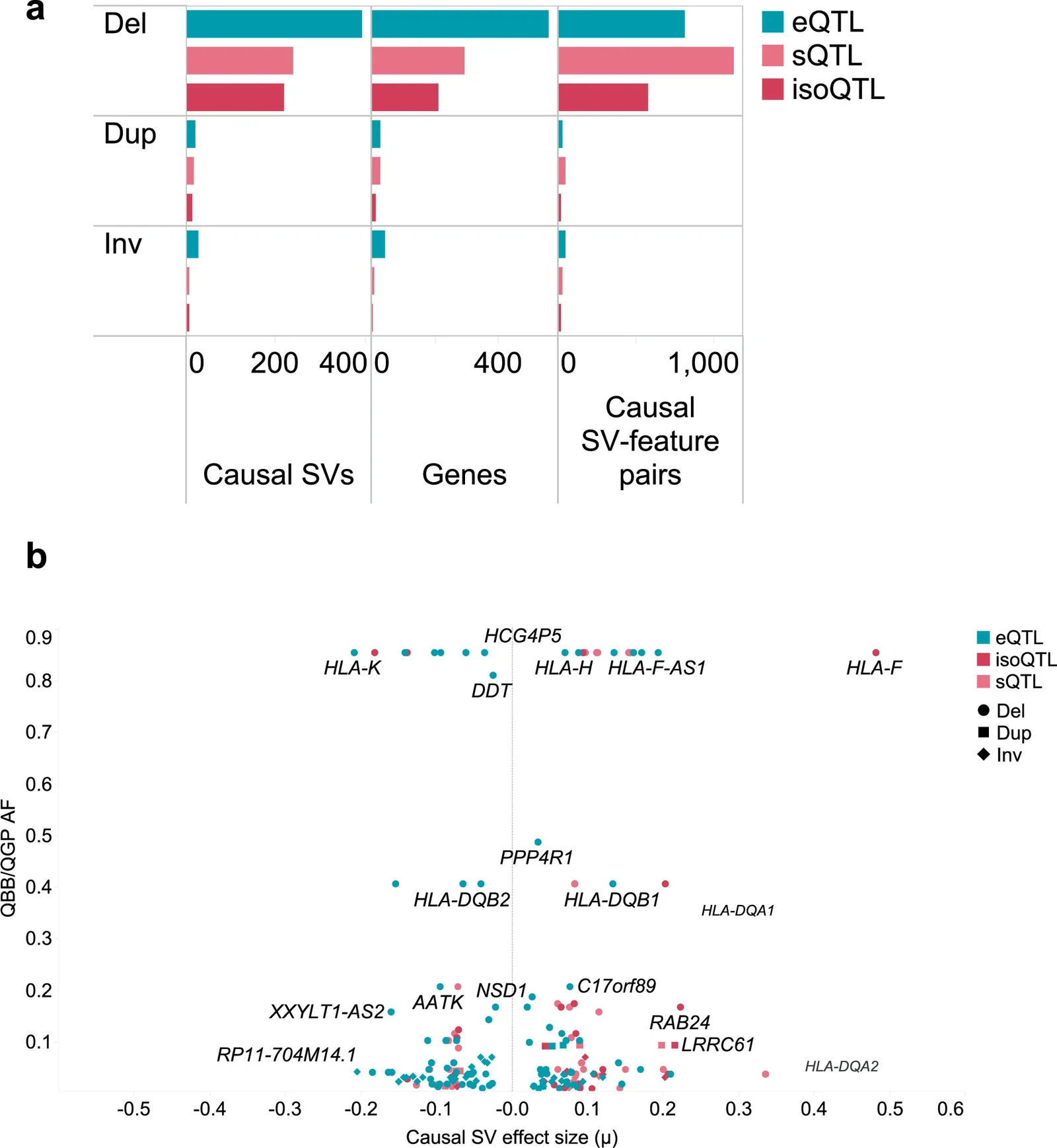
Counts of causal SVs and those only reported in QBB/QGP. **(a)** Counts of fine-mapped SVs, associated genes, and SV–feature pairs identified from a high-confidence set of Del, Ins, and Inv SVs in the QBB/QGP cohort. Fine mapping was performed while conditioning on the earlier fine-mapped SNVs/indels with the respective QTL type and transcriptomic feature. (**b)** Effect sizes (μ) versus allele frequency for the causal SVs reported only in QBB/QGP dataset. Variants are coloured by SV type and shaped by QTL type; gene names are shown where space allows. Source data

We then fine-mapped the SV QTLs using SuSiE while conditioning on fine-mapped SNVs/indels to control for tagging effects (Methods). Effect sizes and LBF distributions were consistent with expectation (Supplementary Fig. 23a). At PIP > 0.95, we identified 948 fine-mapped causal SVs: 443 eQTLs, 266 sQTLs and 239 isoQTLs, collectively affecting 870 genes (FDR < 0.05) (Extended Data Fig. 4a). Del accounted for 853 QTLs, Dup for 50 and Inv for 43, reflecting the tested variant set. Fine-mapped SV QTLs were enriched near TSSs, particularly isoQTLs (Supplementary Fig. 23b), and showed similar size distributions across QTL types (Supplementary Fig. 23c).

**Fig. 3:**
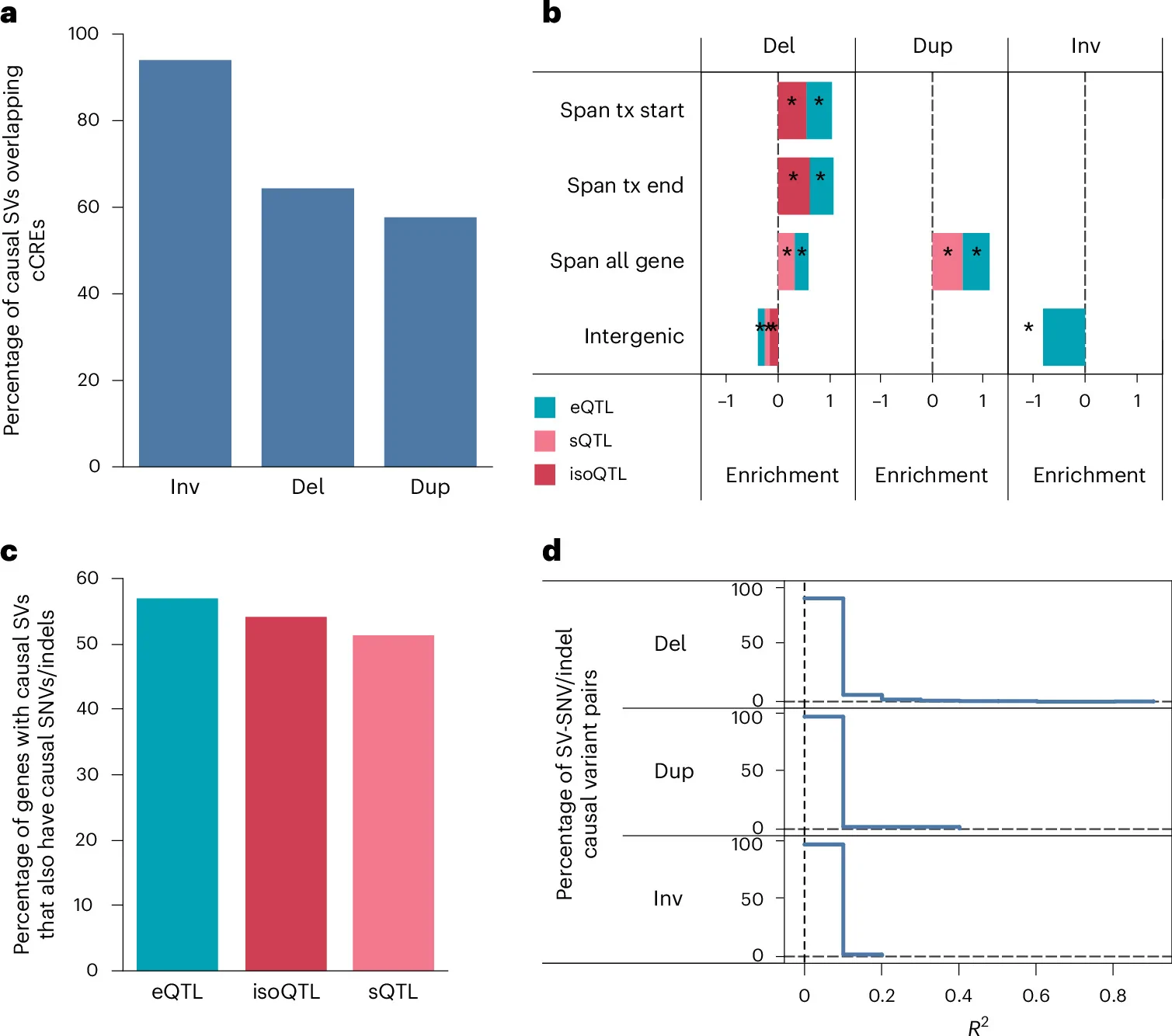
Landscape of causal SVs. **a**, Percentage of fine-mapped SVs where the SV overlaps candidate *cis*-regulatory elements (cCREs). **b**, Enrichment or depletion of causal SVs across different genic elements, shown as the log ratio of SV counts relative to a baseline from all QTL pairs, assessed using a Bonferroni-corrected binomial test. Asterisks indicate significant values (Bonferroni-adjusted *P* < 0.05) as follows: eQTL–Del (Span tx start, 1.59 × 10^−3^; Span tx end, 0.0156; Span all gene, 1.48 × 10^−^^4^; Intergenic, 2.04 × 10^−^^3^), eQTL–Dup (Span all gene, 4.69 × 10^−^^3^), eQTL–Inv (Intergenic, 0.0190), sQTL–Del (Span all gene, 4.78 × 10^−^^5^; Intergenic, 0.0330), sQTL–Dup (Span all gene, 1.02 × 10^−^^3^) and isoQTL–Del (Span tx start, 5.26 × 10^−^^3^; Span tx end, 2.82 × 10^−^^4^; Intergenic, 1.12 × 10^−^^3^). **c**, Percentage of genes having causal SNVs/indels among those with causal SVs for a given QTL type. **d**, Percentage of pairs of causal SNVs/indels and causal SVs for a given QTL type, impacting the same gene, plotted as a function of the coefficient of determination (*R*^2^) between the underlying variants. Source data

Around 60% of fine-mapped Del and Dup QTLs overlapped with genes or critical regulatory elements, compared with 94% for Inv (Fig. 3a). Del eQTLs were enriched at transcription start/end sites and across entire genes but depleted in intergenic regions. Dup eQTLs and sQTLs were enriched for whole-gene overlap, whereas Inv sQTLs were depleted in intergenic regions.

Over half of the genes regulated by causal SVs were also regulated by causal SNVs/indels (58%; *n* = 623) (Fig. 3c). However, most pairs of SV and SNV/indel QTLs per gene and QTL type showed little linkage: 89–97% had *R*^2^ ≤ 0.1 and only 0–0.8% had *R*^2^ ≥ 0.6. This is consistent with the conditional fine-mapping and indicates largely independent regulatory contributions (Fig. 3d).

Fine-mapped SVs spanned broad AF and effect size ranges (Supplementary Fig. 23d). The top 1% by effect size (26 QTL–trait pairs, absolute *μ* > 0.52) impacted 15 genes, including *HLA-DRB1*, *HLA-DRB5*, *HLA-DRB6* and *HLA-C* as well as several lncRNAs and poorly characterized genes. Del QTLs showed a larger variance in terms of *μ* and AF than other SV types (Supplementary Fig. 23e). AF correlated moderately with Genome Aggregation Database (gnomAD) and 1KG (*r* = 0.65, *P* value < 0.0001) (Supplementary Fig. 23f). A total of 70 causal SVs (7.4%) were unique to QBB/QGP and influenced 141 genes (Extended Data Fig. 4b). These included 2 very common Dels (AF > 0.8), one affecting 16 genes across the extended HLA region and another affecting *DDT*, which encodes a cytokine-like protein involved in immune regulation and proliferation^55^.

### Autozygosity and the burden of regulatory variants

**Fig. 4:**
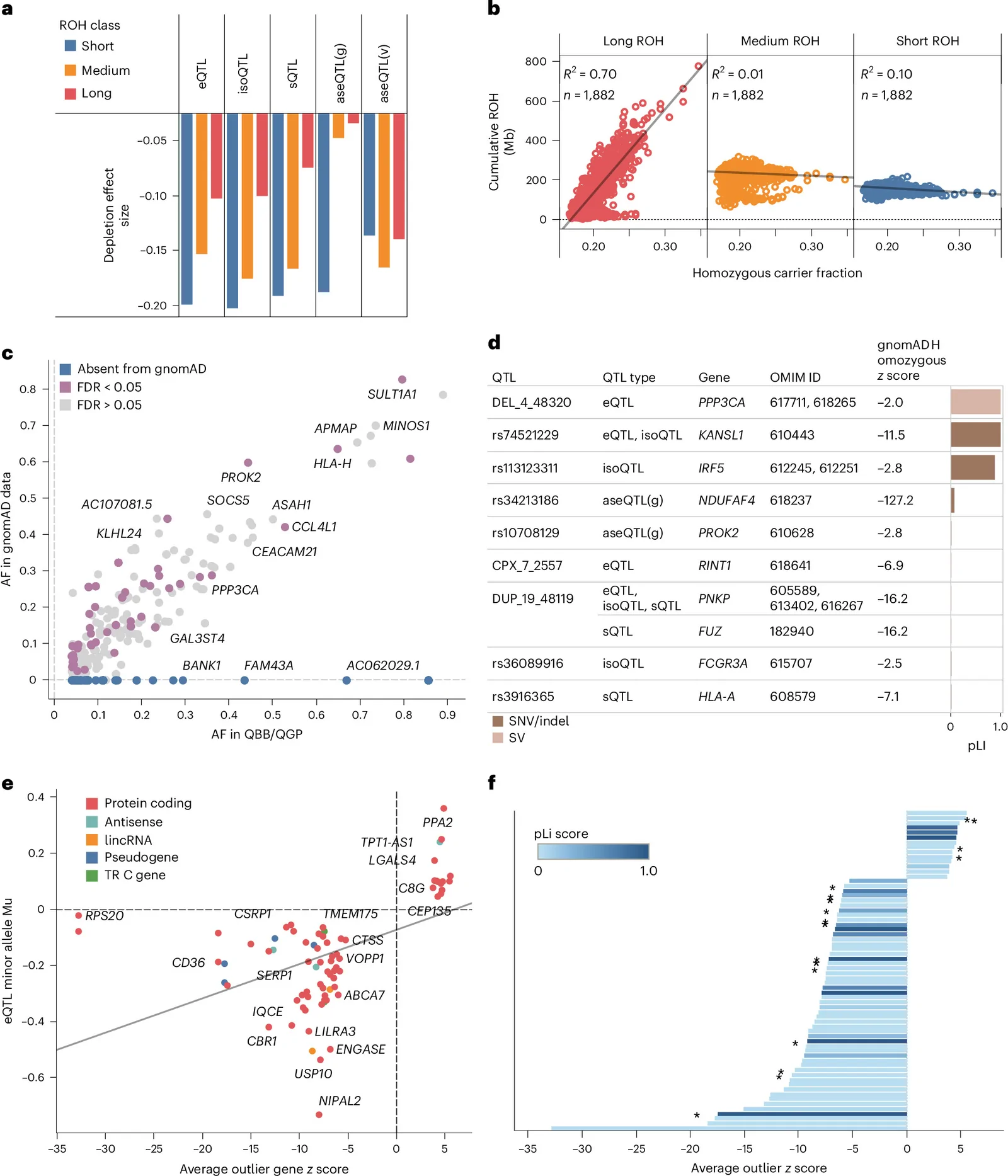
Autozygosity and recessive effects on gene regulation. **a**, Depletion of fine-mapped QTLs from ROHs for various QTL types in short, medium and long ROHs relative to non-ROH regions, assessed using an intercept-only linear model with BH correction for multiple testing. Adjusted *P* values were as follows: eQTL (short, 2.71 × 10^−^^86^; medium, 5.03 × 10^−113^; long, 4.39 × 10^−^^76^), isoQTL (short, 2.23 × 10^−^^34^; medium, 5.27 × 10^−^^55^; long, 1.08 × 10^−41^), sQTL (short, 6.57 × 10^−^^32^; medium, 1.24 × 10^−^^57^; long, 2.93 × 10^−31^), aseQTL(g) (short, 1.01 × 10^−^^85^; medium, 3.63 × 10^−^^18^; long, 1.23 × 10^−^^13^) and aseQTL(v) (short, 7.58 × 10^−^^27^; medium, 3.69 × 10^−^^85^; long, 1.17 × 10^−^^121^). **b**, Homozygous carrier fraction versus cumulative ROH length stratified by ROH class in the QBB/QGP cohort. Each point represents one participant. A strong positive association is observed for long ROH (*R*^2^ = 0.70; *P* value < 0.0001), whereas medium and short ROHs show minimal correlation (*R*^2^ < 0.1; *P* value < 0.0001. **c**, AF in QBB/QGP versus gnomAD for fine-mapped QTLs tested for depletion of minor-allele homozygotes using a one-sided exact binomial test under HWE (*P* < 0.05). Purple points indicate variants passing FDR < 0.05; gray points indicate nonsignificant variants; blue points indicate variants absent from gnomAD. Genes are labeled where space allows. **d**, Subset of FDR-significant QTLs impacting genes with known OMIM disease phenotypes. Bars show gene-level pLI scores (higher = greater loss-of-function intolerance) colored by variant type. QTL class, OMIM phenotype ID and gnomAD replication *z* scores are indicated. **e**, Average *z* scores for eQTL–gene pairs with a significant association between homozygous eQTLs and outlier gene expression (FDR < 0.05), plotted against the SuSiE fine-mapping effect size for the corresponding pairs. **f**, Genes with outlier expression significantly associated with homozygous eQTLs (FDR < 0.05). Bar sizes represent the average *z* score across implicated samples and are colored by pLI score. Genes with OMIM disease phenotypes are marked with a star (*). Source data

**Extended Data Fig. 5:**
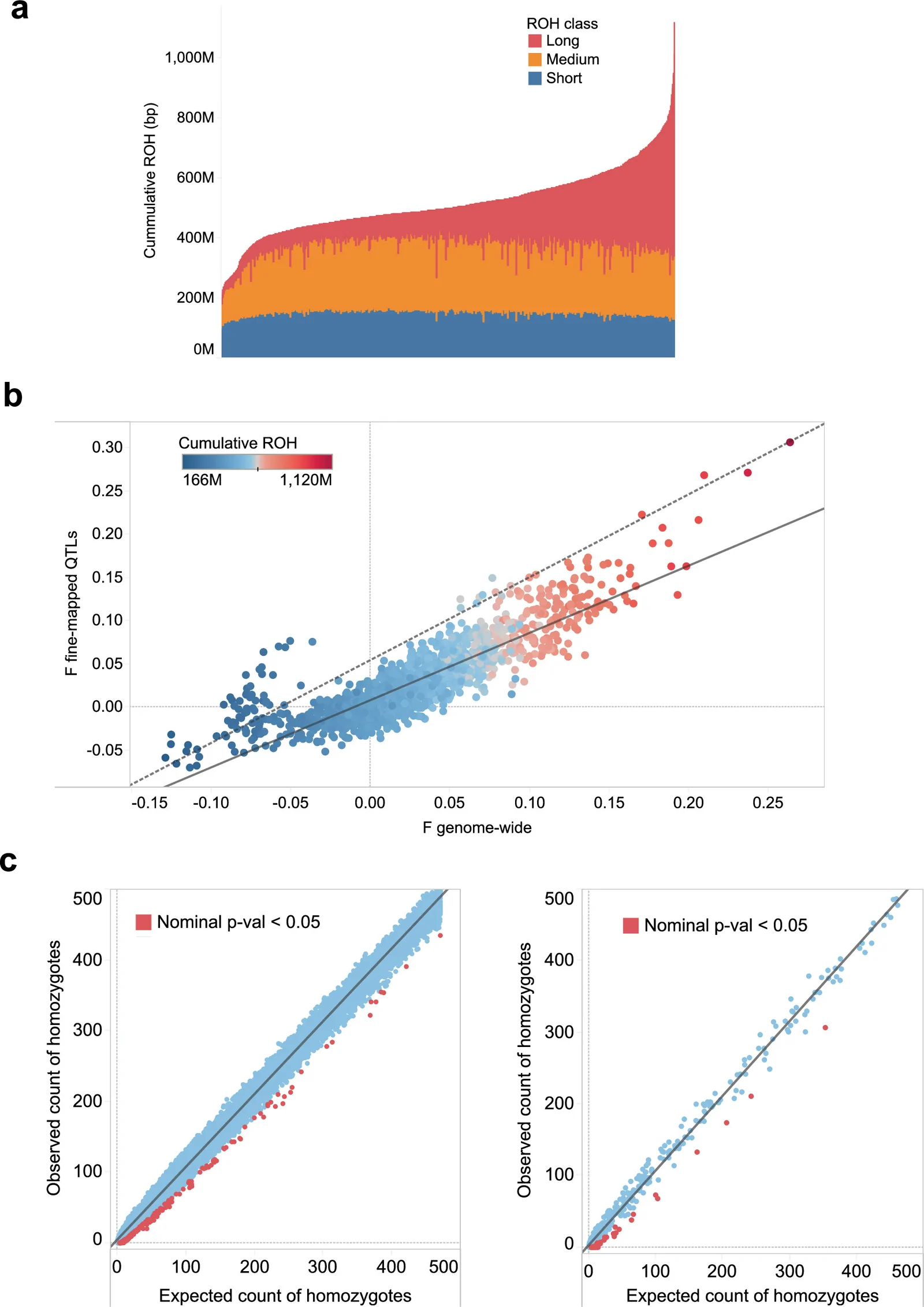
Runs of Homozygosity, Inbreeding across QTLs and causal variants with depleted Homozygotes. **(a)** Stacked cumulative ROH per individual (ordered by total burden), showing the contribution of short, medium and long ROH classes. Higher total ROH is mainly driven by long ROH. **(b)** Per-subject inbreeding coefficients (F) calculated using PLINK v2.0.0-a.6LM (--het) genome-wide versus from fine-mapped QTLs in QBB/QGP. Points are colored by cumulative ROH length. QTL-based estimates strongly correlate with genome-wide inbreeding (R-square= 0.74; p-value < 0.0001) but are systematically attenuated. The unity line is indicated by the dash line. **(c)** Observed minor-allele homozygote counts for fine-mapped QTLs versus expected counts under HWE. Red highlights denote nominally significant p-values (<0.05) from a one-sided exact binomial test. SNV/Indels QTLs are shown on the right; SV QTLs are shown on the left. Source data

The QBB/QGP cohort is characterized by high levels of runs of homozygosity (ROHs), notably medium and long ROHs, reflecting background and recent inbreeding (Extended Data Fig. 5a). Per-sample inbreeding coefficients derived from all variants and from causal variants were strongly correlated (*R*^2^ = 0.74; *P* value < 0.0001), although QTL-based estimates were systematically lower (Extended Data Fig. 5b), indicating that regulatory loci capture genome-wide autozygosity while representing a constrained subset of variation. Fine-mapped QTLs of all types were significantly depleted from ROHs, with stronger depletion in short and medium ROHs (Fig. 4a). This suggests that purifying selection has reduced regulatory variant density in older autozygous segments, whereas recent ROHs have undergone less filtering.

To assess the effect of autozygosity on regulatory burden, we compared, per participant, the fraction of minor-allele homozygous causal variants among carriers (homozygous carrier fraction (H_2_)) with cumulative ROH length (Fig. 4b). H_2_ correlated strongly with long ROHs (*R*^2^ = 0.7; *P* value < 0.0001), but weakly with other classes, indicating that recent autozygosity is the principal driver of homozygous regulatory QTL burden.

### Deleterious recessive regulatory QTLs

Consanguineous populations provide increased power to detect recessive regulatory variants depleted in homozygotes owing to purifying selection. We identified 177 fine-mapped SNV/indel QTLs with significant homozygote depletion (*P* value < 0.05) (Extended Data Fig. 5c and Supplementary Table 4), regulating 201 genes including 35 with established Online Mendelian Inheritance in Man (OMIM) phenotypes^56^. Despite not withstanding multiple-testing correction in QBB/QGP, 33 affecting 38 genes replicated in gnomAD (FDR < 0.05), while 15 were absent from it (Fig. 4c). We also found 48 homozygote-depleted causal SVs affecting 122 genes (*P* value < 0.05) (Extended Data Fig. 5d and Supplementary Table 5), of which 12 affecting 32 genes replicated in gnomAD (FDR ≤ 0.05) (Fig. 4c).

Replicated variants affected 10 genes with known OMIM phenotypes (Fig. 4d), including genes with probability of loss-of-function intolerance (pLI) > 0.9 and 5 associated with recessive disorders: *FCGR3A* (immunodeficiency 20), *NDUFAF4* (mitochondrial complex I deficiency, nuclear type 15), *PROK2* (hypogonadotropic hypogonadism 4), *RINT1* (infantile liver failure) and *PMKP* (ataxia-oculomotor apraxia, Charcot–Marie–Tooth disease, developmental and epileptic encephalopathy). The strongest depletion was for rs77028896, an aseQTL(g) for *NDUFAF4* (*z* score = −127.2). Depletion of homozygotes across these loci supports a deleterious recessive effect, consistent with the inheritance patterns for many of the impacted genes.

### Homozygous causal eQTLs driving outlier expression

**Extended Data Fig. 6:**
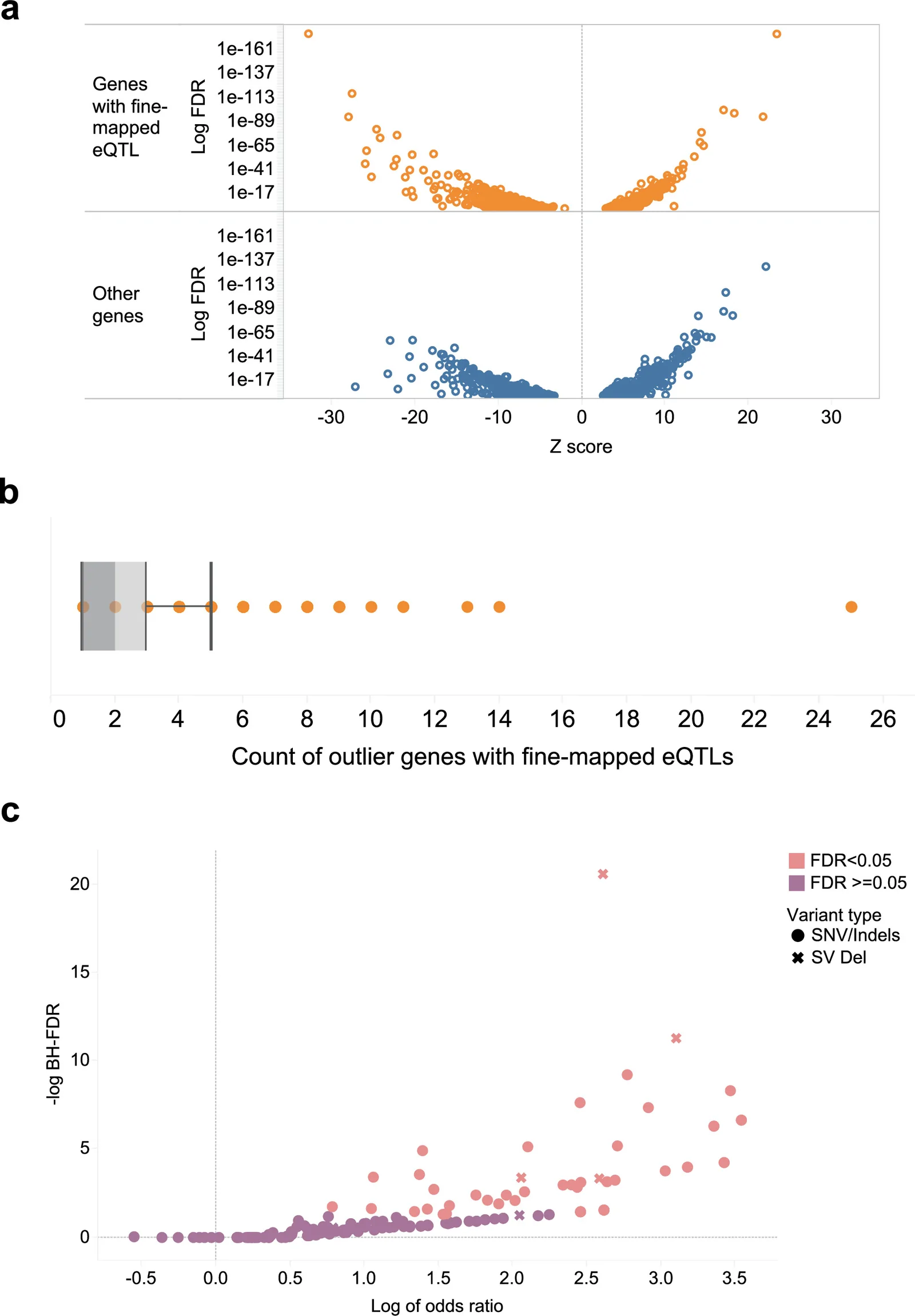
Outlier expression genes and homozygous fine-mapped eQTLs associated with expression-outlier genes. **(a)** Z score versus log FDR for gene-sample tests showing significant outlier expression (FDR < 0.05). Genes with fine-mapped eQTLs are shown in orange while the rest are shown in blue. **(b)** Distribution of count of outlier genes with fine-mapped eQTLs per sample (n = 1,419). The center line of the box plot indicates the median, the limits indicate the first and third quartiles (Q1 and Q3), the whiskers extend to 1.5 × the interquartile range (IQR), and individual points represent individual samples. **(c)** Log odds ratio from Fisher’s exact test evaluating the association between homozygous eQTL carriers and outlier gene expression, plotted against the corresponding FDR. Each data point represents a pair of a causal eQTL and an outlier expression gene. Highlighted are variant classes and significant associations at FDR 0.05. Source data

Next, we examined whether homozygous causal variants drive extreme gene expression, which has important biomedical relevance^57^. Across 1,652 participants, we identified 2,965 outlier genes (FDR < 0.05), with a median of 3 and a maximum of 50 per participant. A total of 1,445 genes (48.7%) had fine-mapped eQTLs across 1,419 participants and showed a *z* score distribution similar to other outlier genes (Extended Data Fig. 6a). Participants carried a median of 2 outlier genes with fine-mapped eQTLs with a maximum of 25 (Extended Data Fig. 6b).

Association testing between homozygous causal eQTLs and outlier gene expression identified 83 significant associations involving 76 eQTLs and 66 genes (FDR < 0.05) (Extended Data Fig. 6c and Supplementary Table 6). Outlier direction was highly concordant with SuSiE fine-mapping effect size, with 80% of associations involving reduced gene expression (Fig. 4e). A total of 12 genes showed moderate to high dosage sensitivity (pLI 0.5–1.0), and 17 had known OMIM phenotypes, mostly recessive (Fig. 4f). These eQTLs are therefore strong candidates for recessive regulatory variants contributing to extreme phenotypes and diseases.

### Colocalization of fine-mapped QTLs with GWAS loci for clinically relevant phenotypes

**Fig. 5:**
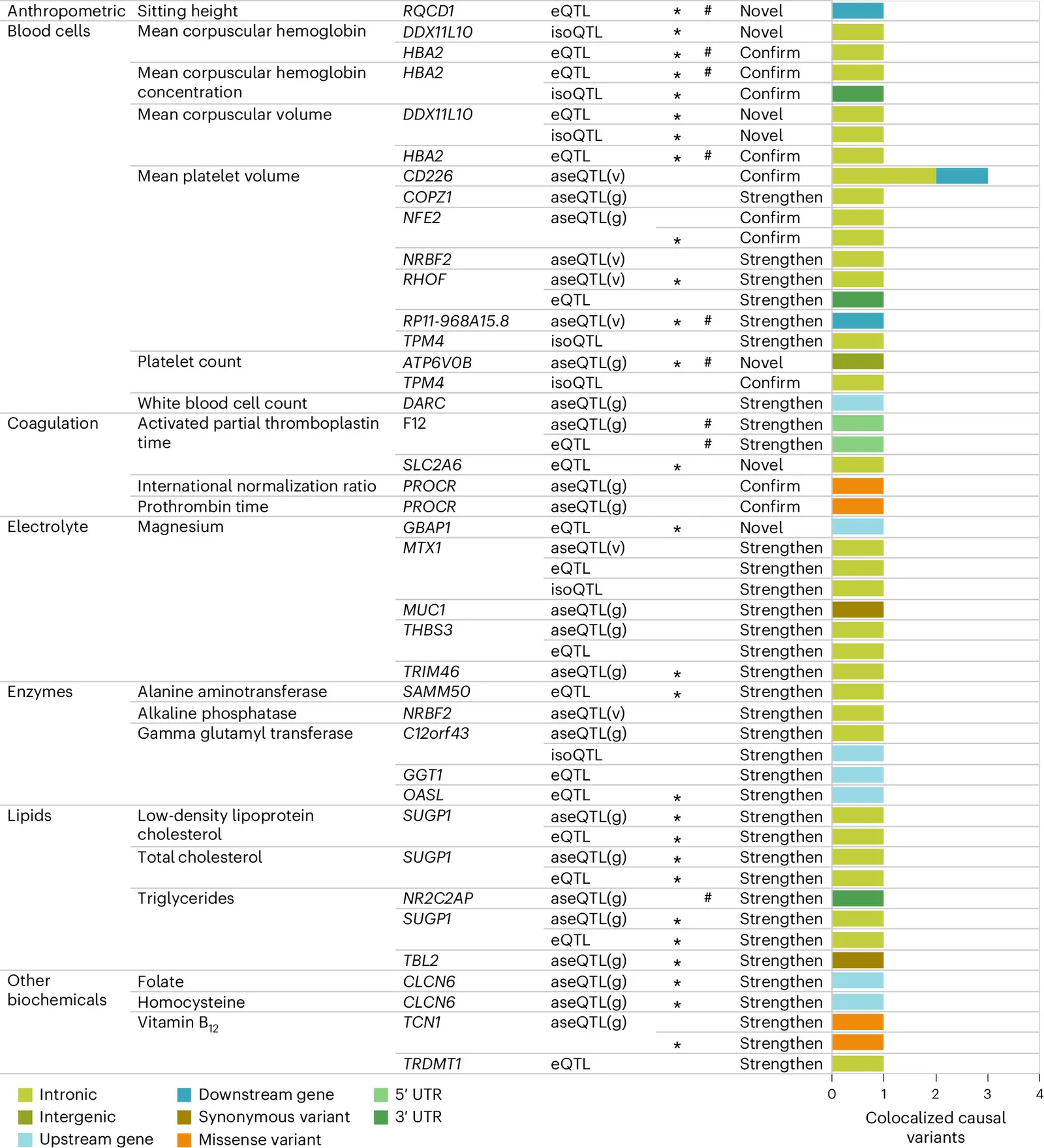
Causal variants colocalized with clinically relevant phenotypes. The identified set of 58 significant colocalizations implicating causal variants in various transcriptomic traits and biobank phenotypes. The colocalizations are grouped by phenotype, indicating the implicated gene and type of the causal variant. QTLs reported only in the QBB/QGP dataset are labeled with a hash (#) and those where the implicated gene is not the nearest to the QTL are labeled with an asterisk (*). Each colocalization is classified according to whether it confirms a well-known relationship (Confirm), strengthens a relationship that is inferred or has suggestive evidence from the literature (Strengthen), or postulates a novel link with plausible mechanistic evidence from the literature (Novel). Bar colors denote variant consequences as annotated by VEP. Source data

Using GWAS of 45 clinically relevant phenotypes in 6,218 QBB/QGP participants^58^, which encompassed the transcriptomic cohort (Supplementary Table 1), we applied SuSiE colocalization to identify shared causal mechanisms (Methods). We detected 58 significant colocalizations (PIP ≥ 0.5; posterior probability of hypothesis 4 (PPH4) ≥ 0.5), comprising 18 eQTLs, 10 isoQTLs, 9 aseQTLs(v) and 21 aseQTLs(g), involving 30 genes and 20 phenotypes (Fig. 5). In 52% of cases, the regulated gene is not the closest to the QTL, and in 17%, QTL–gene pairs were unique to QBB/QGP. Sizes of GWAS effects were generally larger than transcriptomic effects (Supplementary Fig. 25 and Supplementary Table 7). One-third of implicated genes colocalized with the same clinical phenotype through multiple QTL types (Supplementary Table 9). The 20 phenotypes spanned anthropometric, blood cells, coagulation, electrolytes, enzymes, lipids and other biochemical categories. Over half of colocalized QTLs were intronic, 32% were upstream, downstream and in UTRs, four were missense and two were synonymous. Several showed ancestry-specific AF differences (Supplementary Table 10).

A total of 12 colocalizations confirmed established gene–phenotype–disease links involving 5 genes and 5 phenotypes (Supplementary Table 7). For example, rs34466459, a novel eQTL, and rs141514155, an isoQTL, reduced *HBA2* expression and lowered mean corpuscular hemoglobin, mean corpuscular hemoglobin concentration and mean corpuscular volume (MCV), consistent with *HBA2* encoding hemoglobin α-subunit^59^. rs34466459 is predominant in WEP (AF ≥ 0.03), suggesting increased anemia risk in this group (Supplementary Table 10). Also, rs867186 increased *PROCR* allelic imbalance and reduced prothrombin time and international normalized ratio, consistent with the gene’s role in anticoagulation^60^ and association with purpura fulminans^61^, and reduced prothrombin time increases thrombotic risk^62^. Another aseQTL, rs4759076, increased *NFE2* allelic imbalance and reduced mean platelet volume, consistent with *NFE2* function in hematopoiesis^63^, and may therefore protect against myeloproliferative disorders, particularly in South Asians (AF = 0.78).

Nine colocalizations suggested novel causal links involving six variants, five genes and six phenotypes (Supplementary Table 7). For example, rs11898160, a novel eQTL, reduced *RQCD1* expression and increased raised sitting height, consistent with *RQCD1*’s role in cellular differentiation^64^ and potentially linking *RQCD1* with type 2 diabetes and cardiovascular disease, which are inversely associated with sitting height^65^. Also, rs867324077, a novel aseQTL, increased *ATP6V0B* allelic imbalance and platelet count. *ATP6V0B* encodes the vacuolar ATPase component involved in pH regulation and ion transport^66^ and is highly expressed in bone marrow, where platelets are produced in an energy-dependent manner^67^. This variant may therefore lower the risk of thrombocytopenia and increase it for thrombocytosis, both of which are associated with high platelet counts^68^. Another example, rs78641696, reduced *DDX11L10* expression and MCV; a 2-kb deletion in this pseudogene was reported in a patient with low hemoglobin levels^69^, supporting a role of this variant in increasing risk for anemia, known for association with low MCV^70^.

An additional 37 colocalizations strengthened literature-inferred gene–phenotype relationships involving 21 genes and 13 phenotypes (Supplementary Table 7). Three variants affected mean platelet volume: rs7073746 (*NRBF2* aseQTL), rs4326844 (*COPZ1* aseQTL) and rs8104011 (*TPM4* isoQTL), the first two reduced and the third increased MPV. *NRBF2* and *COPZ1* are involved in autophagy, which influences platelet formation and activation^71^, whereas *TPM4* is involved in platelet biogenesis^72,73^. rs8104011 may therefore increase thrombocytopenia risk, while rs7073746 and rs4326844 may protect against myeloproliferative disorders. In addition, two aseQTLs, rs111234557 and rs13235543, increased allelic expression imbalance in *SUGP1* and *TBL2* while reducing low-density lipoprotein cholesterol and triglycerides. *TBL2* locus influences lipid levels^74^ and elevated *TBL2* protein lowers cholesterol^75^. *SUGP1* is associated with low-density lipoprotein cholesterol, coronary artery disease and metabolic phenotypes^76^ and is implicated in cholesterol synthesis and uptake through functional studies^76^. These variants may therefore reduce cardiovascular disease risk. rs13235543 was most common in WEP (AF = 0.33) and GAR (AF = 0.28) (Supplementary Table 10). rs191859379, another example, increased *NR2C2AP* allelic expression imbalance and lowered triglyceride levels, a profile associated with reduced stroke risk^77^. It is found in PAR (AF = 0.01) and GAR (AF = 0.03), but extremely rare globally, suggesting a potentially protective Arab-enriched marker. Final examples involve various metabolites: folate, homocysteine and vitamin B_12_. rs3753587*CLCN6* allelic imbalance lowered folate and raised homocysteine. Low folate is a known risk factor for megaloblastic anemia^78^, homocysteine indicates folate deficiency and chloride inhibits folate binding^79^, strengthening a role for *CLCN6* in folate metabolism. This variant was most frequent in WEP (AF = 0.24) (Supplementary Table 10). Finally, rs9804306 reduced *TRDMT1* expression and vitamin B_12_. *TRDMT1* has been associated with B_12_ status in European and Chinese groups^80^, and B_12_ deficiency is a cause of macrocytic anemia^81^, further supporting the link between *TRDMT1* and anemia.

## Discussion

Understanding the genetic regulation of the human transcriptome is crucial for decoding genome function and enabling precision medicine^19,82,83^. Despite substantial progress, most existing knowledge stems from populations of European descent, limiting its generalizability^18,84–86^. Here we provide a comprehensive QTL resource from a Middle Eastern population by integrating WGS, whole-blood RNA-seq and phenotypic profiling in 1,882 QBB participants. We identified over 7 million significant transcriptome QTLs across 4 regulatory modalities (eQTLs, sQTLs, isoQTLs and aseQTLs) and fine-mapped almost 30,000 high-confidence causal variants. These include over 1,300 previously unreported eGenes and numerous population-specific or globally unreported variants. Integration with biobank phenotypes identified shared causal variants linking transcriptomic traits to 20 clinically relevant phenotypes, providing mechanistic insights into human diseases.

Our fine-mapping uncovered complex regulatory mechanisms, including multivariant and multigene effects. In over half of all fine-mapped QTL–gene pairs, the regulated gene was not the closest to the QTL, highlighting the limitations of assigning regulatory variants by proximity alone. Some genes were influenced by multiple independent regulatory variants, notably within the highly polymorphic *HLA* locus. *FOLR3* harbored the largest number of independent causal eQTLs, making them compelling for future studies of folate metabolism and fetal development^52^. We also identified previously unreported noncoding variants affecting medically actionable genes and may modulate disease risk as per the liability threshold model^87^. Although noncoding variants are often overlooked in clinical genetic reports, improved understanding of their regulatory mechanisms will facilitate their incorporation into clinical assessment^88^. The enrichment of QTLs among synonymous variants further supports the notion that evolutionarily neutral coding variants can exert important functional regulatory effects^19,89,90^.

Our study reveals extensive regulatory diversity and ancestry specificity. A quarter of fine-mapped QTLs were undetected in previous studies, 4% were absent from public variant databases and 21% showed marked allele frequency divergence relative to global populations, reflecting selective pressure related to adaptation to local diet and pathogens^11,23,91–93^. A total of 778 fine-mapped QTLs differed significantly across major Arab subpopulations, emphasizing the value of studying underrepresented populations to improve interpretation and equitable genomic medicine^36,38,40,94^.

Considering SVs substantially increased the completeness of regulatory variant discovery^95–97^. We identified 948 causal SVs, independent of short variant effects, including 70 absent from public databases. They regulated 870 genes with diverse functions, including polymorphic immune and disease-associated loci in the *HLA* region. These findings support the routine inclusion of SVs in transcriptomic studies and diagnostic pipelines^98–100^.

The elevated autozygosity of consanguineous populations provides a valuable framework for investigating recessive regulatory variation. We demonstrate that regulatory QTLs are depleted from ROHs, highlighting purifying selection against regulatory heterozygosity loss^101^. The burden of homozygous regulatory variants increases with long ROHs, indicating that recent parental relatedness predominantly drives the exposure of recessive regulatory effects. Extending on studies of coding variation in consanguineous populations^102^, our findings show the merit of transcriptomic QTL mapping in these populations for identifying plausible deleterious recessive regulatory variants.

Colocalization analysis identified shared causal variants at 58 loci across 30 genes and 20 phenotypes. These included established relationships, such as *HBA2* with red blood cell traits and *PROCR* with coagulation phenotypes, as well as novel regulatory links including *ATP6V0B* with platelet count and *RQCD1* with anthropometric traits. The implication of hematologic, lipid metabolism and vitamin metabolism pathways underscores the clinical relevance of transcriptomic QTLs for interpreting noncoding variants in complex diseases^103–105^. Several colocalizations also involved ancestry-divergent variants, suggesting population-specific disease risk. For example, rs34466459 (causal eQTL *HBA2*; lowers mean corpuscular hemoglobin concentration) is predominant in WEP, where it may increase anemia risk, while rs191859379 (causal aseQTL for *NR2C2AP*; lowers triglyceride) is more common in Arab groups, where it may protect against stroke and cardiovascular diseases. In over half of the colocalizations, the implicated gene was not the closest to the QTL, consistent with the distal regulatory mechanisms mostly captured by GWAS^106^. Several implicated genes are linked to diseases with increased prevalence in Middle Eastern populations^107,108^.

Our study underscores the value of incorporating transcriptomics into national biobanks, which has been limited so far^109–111^. Beyond improving interpretation and prioritization of genetic variation for functional follow-up, it would allow multimodal analyses within the same cohort, enabling more robust analysis of intermediate traits in complex disease pathways. Moreover, conducting large-scale RNA-seq studies at a single site mitigates confounding factors associated with multicenter designs^112^.

Although whole blood does not capture all tissue-specific regulatory effects, it provides accessible, clinically relevant and scalable tissue for population-scale studies, particularly for immune and metabolic traits^18,20,21,113–117^. Future studies should extend analysis to other tissues and to single-cell and spatial transcriptomics while functionally validating new novel findings. Together, our dataset provides a foundational resource for transcriptomic regulation in the Middle East and shows how population-specific genomics can accelerate biological discovery with translational clinical insights.

## Methods

### QBB/QGP study samples

The samples used in this study were obtained from QBB. They comprised predominantly healthy Qatari adults (age ≥ 18 years) randomly recruited from the general population in Qatar. Written informed consent was obtained from all participants, and institutional review board approvals for the study were secured from QBB and Sidra Medicine (1604003028). Sequencing was performed at Sidra Medicine.

### Blood QTL public datasets

The GTEx V8 dataset was downloaded from the GTEx portal^19^. It comprised *cis*-eQTL and *cis*-sQTL data from 838 individuals, including 715 of European American, 103 of African American and 12 of Asian American descents^118^. A mapping table was downloaded from the GTEx portal to assist with coordinate liftover. GALA II/SAGE datasets were obtained from the link provided by Kachuri et al.^21^, consisting of *cis*-eQTL data for African Americans (AA), Puerto Ricans (PR) and Mexican Americans (MX). Regarding the eQTLGen dataset, significant *cis*-eQTLs were obtained from the eQTLGen Consortium website (release 2019-12-11), based on a multicohort collection of 31,684 blood samples, largely of European ancestry^20^. For the Sardinia dataset on 642 participants from Sardinia, *cis*-eQTLs, *cis*-aseQTL and *cis*-isoQTLs (Bonferroni *P* value < 0.05) were obtained from Pala et al.^17^. With regard to the Depression Genes and Networks study, *cis*-eQTLs, isoQTLs and aseQTLs were extracted from Battle et al.^119^ and Pala et al.^17^. Furthermore, eQTL counts from the Framingham Heart Study were obtained from Liu et al.^120^. For the GEUVADIS dataset, *cis*-eQTLs and aseQTLs were extracted from Lappalainen et al.^121^ and Pala et al.^17^. Additionally, fine-mapped whole-blood eQTLs, isoQTLs and sQTLs were obtained from the eQTL Catalogue^122^, which combines data for GTEx V8 (ref. ^19^), Lepik et al.^123^ and TwinsUK^124^ studies (PIP > 0.95).

### WGS, variant calling and QC

WGS data generation and processing were performed previously as described in Razali et al.^36^. Briefly, DNA was extracted using the Qiasymphony automatic system (QIAGEN). PCR-free libraries were generated using standard procedures. Sequencing was performed using HiSeq X Ten (Illumina) with 150-bp paired-end reads, achieving 30× mean coverage, with a median insert size of 400 bp ± 25%. Next, the reads were aligned against the GRCh37 reference using BWA-MEM v0.7.7 (ref. ^125^). SNVs and indels were obtained using joint variant calling with Genome Analysis Toolkit v3.4 (ref. ^126^). QC was performed to assess heterozygosity levels and genotype–phenotype sex concordance. For downstream analysis, we retained variants with PASS filter label, missingness level < 1%, MAF > 1% and Hardy–Weinberg equilibrium (HWE) *P* value > 10^−6^ (ref. ^17^). This resulted in 7,448,595 biallelic SNVs and 1,944,126 indels (Supplementary Table 2). For SVs, the calls were generated for Del, Dup and Inv using a consensus pipeline based on ten callers^127^: Breakdancer v1.4.5 (ref. ^128^), Breakseq2 v2.2 (ref. ^129^), CNVnator v1.0.0 (ref. ^130^), Delly v0.7.8 (ref. ^131^), ERDS v1.0.2 (ref. ^132^), Genome STRiP v2.00.171 (ref. ^133^), Manta v1.6.0 (ref. ^134^), SpeedSeq v0.1.2 (ref. ^135^), SvABA v0.2.3 (ref. ^136^) and WHAM v1.7.0 (ref. ^137^). Consensus SV calls from these methods were produced per sample, followed by merging at multisample level using SURVIVOR v1.0.7 (ref. ^138^). The same thresholds of missingness, MAF and HWE *P* value as above were applied, yielding 10,522 high-quality SVs (Supplementary Table 2).

### RNA extraction, QC and globin depletion

Total RNA was extracted on Hamilton Chemagic Star platform using Chemagic STAR Total RNA PAXgene MV Kit (Chemagen) and on Qiasymphony automatic system (QIAGEN) and stored at −80 °C. RNA quantity and quality were assessed using DNF-471 Standard Sensitivity RNA Analysis kit on a Fragment Analyzer (Agilent), yielding >1 μg for most samples (Supplementary Fig. 1a). RNA quality number was calculated using ProSize Data Analysis Software, retaining samples with ≥7 for downstream application (Supplementary Fig. 1b). Next, as recommended^139^, we applied globin depletion on the RNA samples using the GLOBINclear Kit (Thermo Fisher Scientific)^140^ to increase the sensitivity to detect nonglobin RNAs. This kit uses nonenzymatic magnetic beads to deplete more than 95% of the alpha and beta globin mRNAs. Standard vendor protocol was followed, using a minimum input of 1 μg of RNA per sample.

### Library preparation and RNA-seq

Globin-depleted RNA integrity and concentration were further assessed using the sensitivity RNA assay on a Perkin Elmer Caliper Labchip GXII. For a set of 2,028 samples passing QC, 500 ng of RNA was used for library preparation using the Illumina TruSeq Stranded mRNA kit. Poly-A RNA was selected using an Oligo-dT magnetic bead system, followed by fragmentation, first-strand synthesis using Superscript IV and second-strand synthesis. cDNA obtained after reverse transcription was ligated with TruSeq RNA Combinatorial Dual Index adapters and amplified for 15 cycles. Library quality and concentration were assessed using DNA 1k assay on the Perkin Elmer Caliper Labchip GXII. Libraries were quantified using a KAPA HiFi Library quantification kit on Roche LightCycler 480. Cluster generation was performed on cBot 1.0 or 2.0 using HiSeq 3000/4000 PE Cluster Kit and HiSeq 3000/4000 SBS Kit (300 cycles). Flow cells were loaded at a cluster density of 1,310–1,524 K/mm^2^ and sequenced on an Illumina HiSeq 4000 instrument with 150 pair-end reads at medium (14–41 million, median = 22 million) or high depth (55–140 million, median = 70 million) (Supplementary Fig. 1c).

### RNA-seq reads QC and alignment

For the sequenced RNA samples, raw reads were filtered for quality in three steps. First, Trimmomatic was used to trim the Illumina TruSeq adapter sequences from RNA-seq paired-end reads^141^. Second, reads mapping to the human ribosomal RNA databases (Silva and Rfam) were filtered out using SortMeRNA^142^. Third, reads were screened for vector sequences by mapping to the NCBI UniVec database^143^ using SeqyClean^144^. Overall, we observed a 4% drop in the median of raw reads (Supplementary Fig. 1d). Next, we ran FastQC v0.11.2 and MultiQC v1.21 (ref. ^145^) to assess QC at the sample level and identify problematic samples based on GC content, leading to the exclusion of 71 participants from the sequenced cohort. Subsequently, QCed reads were aligned against the GRCh37 reference using STAR v2.5.0a^146^. For splicing and allelic expression, we used STAR v2.7.3a, which allowed the aspOutputMode option, and a genotype file to account for allelic mapping biases^19^. Reads that did not pass WASP filtering (that is, having the tag vW:i:[2–7]) were filtered out.

### Sample-level matching of WGS and RNA-seq data

We assessed accidental sample swaps and contamination by running verifyBAMID v1.1.3 (ref. ^147^). For a given participant, this checks consistency between RNA-seq reads in a BAM file and the corresponding genotype calls in a WGS VCF file. For each participant, we run a one-to-many analysis with –best option, using as input a given RNA BAM file and a multisample VCF corresponding to all the samples in the QBB/QGP dataset. This generated Chipmix, Freemix and identity-by-descent scores for a given participant against all the samples in the VCF file, including the best-matching sample (Supplementary Fig. 2). We identified 4 participants for which there were accidental sample swaps and 43 participants with probably contaminated samples (Chipmix and/or Freemix scores > 0.2, low identity-by-descent scores, as recommended^147^), in addition to 32 replicate samples. Therefore, we retained 1,882 high-quality RNA-seq samples for downstream analyses, with a respective coverage of 14–90 million reads.

### Quantification of transcriptome traits

#### Gene expression

Sequencing reads were aligned to Ensembl genes (GRCh37; release 75) using Subread featureCounts^148^ and subsequently converted to fragments per kilobase of transcript per million mapped reads (FPKM) values^149^. Genes with a mean FPKM < 0.1 across all samples were filtered out, leaving 14,484 genes (13,942 autosomal). Variance stabilization was applied to these genes using DESeq2 (ref. ^149^), followed by rank-inverse normal transformation using the R package RNOmni^150^.

#### Isoform expression

We performed isoform quantification using Salmon^151^ with the Ensembl reference (GRCh37; release 75). Isoform proportions were calculated for each isoform as the ratio of isoform FPKMs to the sum of FPKMs per gene. A mean FPKM threshold of 0.1 was applied. Genes with a single expressed isoform were filtered out. A total of 31,455 isoform ratios were obtained, and their expression levels were subject to rank-based inverse normal transformation using the R package RNOmni^150^.

#### Splicing

LeafCutter^11^ was used to quantify splicing based on intron excision phenotypes as follows: First, intron usage was quantified using bam2junc.sh script in LeafCutter. Next, cluster_prepare_fastqtl.py script from the GTEx pipeline (https://github.com/broadinstitute/gtex-pipeline/tree/master/qtl/leafcutter/src)^19^ was executed as a wrapper to run the following steps: (1) intron clusters were identified using leafcutter_cluster.py script with the options --min_clu_reads 30 --min_clu_ratio 0.001 --max_intron_len 500000; (2) intron clusters were mapped to genes using the map_clusters_to_genes.R script with exon coordinates derived from the Ensembl gene annotation (GRCh37; release 75); (3) introns with few counts or low complexity were filtered out; and (4) the filtered data were normalized using prepare_phenotype_table.py script, and the resulting per-chromosome files were merged and converted to BED format with the start and end positions corresponding to the TSS of the gene where the introns mapped. Finally, the results from the wrapper script were rank-based inverse normalized using the R package RNOmni^150^.

#### Allele-specific expression

To generate variant-level allele-specific expression data, we used phASER v1.1.1 as follows^152^: First, phaser.py was run on the phased variants and WASP-filtered BAM files to generate files containing haplotype details and read count statistics per sample. The ‘--blacklist’ option was applied to eliminate *HLA* gene variations in addition to the ‘--haplo_count_blacklist’ option to exclude sites with known issues, as described in ref. ^10^. Next, variant-level allelic expression was calculated as the ratio of reference allele read counts over the total depth at each heterozygous site, and allelic imbalance (AI) was calculated as the absolute deviation of this ratio from 0.5 (ref. ^17^). We considered heterozygous sites with at least 30 reads per sample, where each allele accounts for at least 2% of the total reads^17^, has AI > 0 in at least 10% of the samples and occurs within expressed genes (mean FPKM > 0.1). The resulting AI matrix contained 11,182 heterozygous variants and was rank-inverse normalized using RNOmni^150^.

To generate gene-level haplotype counts, we run *phASER Gene AE* with a minimum haplotypic minor-allele frequency (--min_haplo_maf) of 0.05. Next, phaser_expr_matrix.py script was used to compile the results to a single matrix, which was subsequently used to calculate gene-level allele-specific expression as the ratio of read counts from a given haplotype over the total number of reads per gene, followed by calculating AI as the absolute deviation of this ratio from 0.5. Genes with AI > 0 in at least 10% of the samples were retained. The resulting AI matrix containing 11,189 genes was rank-inverse normalized using RNOmni^150^.

### Discovery of univariate QTLs

#### Identification of PEER factors and other covariates

We generated probabilistic estimation of expression residue (PEER) factors to use as covariates for QTL analysis^153^. To optimize the number of PEER factors, we counted significant eQTLs (FDR < 0.05, using Matrix eQTL)^154^ as a function of PEER factors, based on a random set of 1,500 genes. An interval of 5 was used across six replicates. This identified 45 as an optimal number of PEER factors (Supplementary Fig. 3). Furthermore, we compiled other sample metrics, including age, BMI, gender, counts of different white blood cell types, RNA extraction and sequencing batches, alignment metrics, genetic principal component analyses and ancestry groups (Supplementary Table 3). Pearson correlation was calculated among these metrics, allowing the selection of relatively independent variables to use alongside the 45 PEER factors as covariates for QTL analysis (Supplementary Fig. 4).

#### Calculation of univariate QTLs

We used an established pipeline based on FastQTL v2.184 (ref. ^155^) to map various types of *cis*-QTLs, specifying a window of 1 Mb upstream and downstream of TSS for a given gene (Supplementary Fig. 5). For eQTLs, we used as input the quantified gene-level expression data and covariates (Supplementary Fig. 5a). We run FastQTL in nominal pass mode to compute nominal *P* values for all variant–gene pairs, then we run it in permutation pass mode using the parameter --permute 1000 10000. Using outputs from both modes, we run outputs.py script from FastQTL to obtain significant eQTL pairs at a FDR threshold of 0.05. For isoQTLs, we run a similar approach to eQTLs (Supplementary Fig. 5b), using as input quantified isoform-level expression data and tested variant–isoform pairs involving variants within 1 Mb on either side of the TSS of a given transcript. Regarding sQTLs, we run the same pipeline using quantified intron excision ratios while applying the --grp in the permutation step to jointly determine the empirical *P* value over all intron clusters of a gene (Supplementary Fig. 5c). Furthermore, we performed aseQTL mapping at the v and g levels by running the FastQTL pipeline using quantified allele expression data as input (Supplementary Fig. 5d,e). Genotypes in the input VCF file were classified as either homozygous or heterozygous following Pala et al.^17^. Also, calculateSignificanceFastQTL.R script from FastQTL v2.184 was run on the permutation output to adjust the *P* values using the *q*-value R package with the pi0 = 1 parameter and to calculate the pval_nominal_threshold per phenotype^156^. Quantile–quantile plots for the various QTL analyses were generated using qqman v0.1.9 (ref. ^157^) (Supplementary Fig. 6).

### Fine-mapping of QTLs

We used susieR package^16^ to test univariate *cis*-QTLs (within 1 Mb of TSS) and generate credible sets of causal variants (Supplementary Fig. 11). This method calculates approximate posterior distributions for multiple causal-variant fine-mappings by summing individual single-effect vectors, whereby each vector represents a potential causal variant. For improved accuracy, we run the method using genotypes as input instead of summary statistics. First, we coded the genotypes as dosage using process.pl script from the GTEx pipeline (https://github.com/xqwen/dap/tree/master/gtex_v8_analysis). Then, we processed the genotype and expression matrices (rank-based inverse normal transformed) using remove.covariate.effects function from susieR to account for confounding factors. Next, we run the fine-mapping step using susie function from susieR with the following parameters: *L* = 10, coverage = 0.95, compute_univariate_zscore = TRUE and max_iter = 1000. We retained credible sets with lead variant having PIP > 0.95 and LBF > 2, as recommended^158^. We removed any given credible set, where a lead variant is correlated (linkage disequilibrium (LD) *R*^2^ > 0.25) with lead variants from other credible sets with a higher effect.

SV QTL fine-mapping was performed by conditioning on fine-mapped SNV/indel QTLs for the same QTL type and common transcriptomic feature. For each gene, a partial regression framework was applied, adjusting gene expression for standard covariates together with the dosages of the conditioning SNV/indel variants. Residualized expression values were obtained using remove.covariate.effect, with a design matrix comprising both technical covariates and SNV/indel dosages. To account for linkage disequilibrium between SNVs/indels and SVs, SV dosages were residualized using the same design matrix, yielding SV genotype residuals orthogonal to both covariates and SNV/indel effects. SV fine-mapping was then performed using SuSiE with residualized expression as the phenotype and residualized SV dosages as predictors. Posterior inclusion probabilities and credible sets were computed as performed for SNV/indel QTLs.

### Colocalization of fine-mapped QTLs with GWAS loci

We performed colocalization of fine-mapped QTLs with GWAS loci associated with 45 clinically relevant traits, obtained from a GWAS conducted by Thareja et al. on 6,218 participants from the QBB/QGP cohort, including the current study cohort^58^. We used coloc v5.1.0 R incorporating the SuSiE framework for fine-mapping in the presence of multiple causal variants^159,160^ (Supplementary Fig. 24). First, we downloaded GWAS summary statistics^58^ from the GWAS catalog (https://www.ebi.ac.uk/gwas/publications/33623009) and converted to VCF format, for which we fixed REF and ALT allele swaps using bcftools +fixref and adjusted beta sign accordingly^161^. Next, we conducted fine-mapping of eQTLs using the susieR susie with parameters 95% coverage and max_iter 1,000, using as input individual-level genotypes, expression data and covariates. Subsequently, we fine-mapped GWAS variants using coloc runsusie providing as input GWAS summary statistics and LD matrices calculated from the QBB/QGP dataset, resulting in 406 fine-mapped loci. Using the fine-mapped results from both QTL and GWAS, we run coloc R coloc.susie pipeline to test colocalization across QTL types whereby there is at least one GWAS variant with *P* value < 5 × 10^−8^ and one QTL with an FDR < 0.05 in any region of 1 Mb upstream and downstream of the TSS of a given transcriptome feature (number of tests = 14,255). Ultimately, this generated the following: (1) a summary table with posterior probabilities of hypotheses H1, H2, H3 and H4 relating to each colocalization test; and (2) a results table containing the PIPs for the top variant being jointly causal for both QTL and GWAS traits, assuming H4 is true. On the basis of these outputs, we selected pairs of credible sets that have PPH4 ≥ 0.5 and where SNP PIP ≥ 0.5.

### Comparison of QTL allele frequencies between populations

We performed pairwise comparisons of QTL allele frequencies between populations by applying a GLM in glm R v4.1.3. For each variant, allelic dosage (0, 1 or 2) was derived per participant in each population. The GLM, genotype ~ population, was applied for a given population pair based on the quasi-Poisson family distribution derived from the dosage values with a log link function. R multcomp package (v1.4.19) was used for fitting linear contrasts on the GLM fit comparing AF for predefined pairs of populations for a given variant^162^. The Bonferroni procedure was used to correct for multiple testing^163^.

### Variant functional annotation

SNV and indel QTL variants were annotated using VEP v99 (ref. ^41^), based on the Ensembl database v99 (ref. ^164^) and GRCh37 (ref. ^165^). SV QTL variants were annotated using AnnotSV^166^ and SVAnnotate - Genome Analysis Toolkit v4.4.0.0 (ref. ^167^).

### Enrichment of QTLs in genomic features

We used a binomial test to calculate the enrichment of QTLs in various genome features as annotated by VEP. For each QTL type and genome feature, we calculated the log ratio of significant over tested QTLs, both normalized for baseline variant count as derived from the VCF file. The Bonferroni procedure was applied to correct for multiple testing^163^.

### Pathway analysis

We conducted pathway analysis of QTL genes using Ingenuity Pathway Analysis (QIAGEN), which relies on curated functional annotations, biological pathways and disease associations.

### Venn diagrams and UpSet plots

Venn diagrams were generated using ggVennDiagram v1.2.3/v1.5.2/v1.7.3 (ref. ^168^). UpSet plots were generated using ComplexHeatmap v2.1.1 (ref. ^169^).

### Identification of ROHs

ROHs were identified with bcftools roh (v1.22) using default parameters^170^. They were called on autosomal variants passing QC, retaining segments with Phred quality scores > 50 and exceeding default thresholds for minimum length and marker density. As recommended^171^, population-specific boundaries for short, medium and long ROH classes were obtained by fitting a Gaussian mixture model (*G* = 3) to the genomic lengths of the ROH segments, implemented in mclust in R^172^. The resulting clusters were ordered by their mean length, resulting in the following ROH boundaries: short (<182 kb), medium (182–864 kb) and long (>864 kb).

### Enrichment of fine-mapped QTLs within ROHs

Cohort-level enrichment of QTLs within ROHs was derived from participant-level ratios of QTL counts inside versus outside ROH. For each participant and ROH class, the ratio was defined as the number of fine-mapped QTL variants overlapping ROH divided by the number of fine-mapped QTL variants outside ROH, both terms normalized by the corresponding count of other tested variants. Ratios were log_10_-transformed and aggregated within each ROH class using a linear intercept-only model implemented with the lm() function in R (stats package, v4.3.2) to test whether the mean ratio across participants differed from the null expectation. Statistical significance was assessed using intercept tests, with multiple-testing correction applied per QTL type using BH FDR via p.adjust() in the stats package.

### Fraction of homozygous QTLs among carriers

The fraction of minor-allele homozygous QTLs among carriers (H_2_) was calculated per participant as *N*_11_/*N*_11_ + *N*_01_, where *N*_11_ denotes the count of minor-allele homozygotes and *N*_01_ denotes the count of heterozygotes across all QTLs.

### Identification of QTLs with depleted homozygotes

For each QTL variant, we tested whether the minor-allele homozygous genotype occurred less frequently than expected under HWE, using base R functions. The minor allele was defined based on AF: variants with AF ≤ 0.5 were tested for depletion of 1/1 genotypes, whereas variants with AF > 0.5 were tested for depletion of 0/0 genotypes. Under HWE, the expected probability of the minor-allele homozygote is AF^2^ for AF ≤ 0.5 and (1 − AF)^2^ for AF > 0.5. Depletion was assessed using a one-sided exact binomial test comparing the observed minor-allele homozygote count with its HWE expectation, implemented in the binom.test() function from the stats package. As a standardized effect size, we computed the *z* score by subtracting the expected count from the observed count and dividing the result by the square root of the binomial variance; negative *z* scores indicate depletion. Multiple testing correction was performed using BH FDR via p.adjust() in the stats package. Variants with nominal significance (*P* < 0.05) were tested for replication in gnomAD (v2.1.1) using the same approach.

### Identification of genes with outlier expression

Outlier gene expression was detected using OUTRIDER R package (v1.20.0) using default parameters^173^. This involved filtering RNA-seq count data to remove low-expression genes, normalization to account for library size differences and applying a denoising autoencoder to capture latent factors, reflecting technical and biological variation across samples. For each gene–sample pair, OUTRIDER computed *P* values and associated *z* scores based on deviations from the fitted model. Genes were considered expression outliers in a given sample if they showed statistically significant deviation after multiple testing correction (FDR ≤ 0.05).

### Homozygous fine-mapped eQTLs associated with expression outlier genes

For each gene showing expression outliers, we tested whether homozygosity at its fine-mapped eQTL variant was associated with outlier status for that gene. Homozygous genotypes were defined as 1/1 when alternate AF ≤ 0.5 and as 0/0 when alternate AF > 0.5, thereby ensuring that the minor-allele homozygote was evaluated. For each gene–variant pair, a 2 × 2 contingency table was constructed and association was assessed using Fisher’s exact test (fisher.test). Analyses were done on genes that are outliers in ≥3 participants and eQTL variants with ≥3 tested homozygotes. Multiple testing correction was performed using BH with p.adjust. All analyses were conducted in R using the stats package (v4.3.2).

### Statistics and reproducibility

Sample size was not predetermined because the study cohort already exceeds that required for statistically significant discovery based on previous studies. No data were excluded except as part of the QC and filtering procedures as described in Methods. Experiments were not randomized, and investigators were not blinded. Pearson correlation coefficients with two-sided significance testing were used. Appropriate multiple testing correction was applied using FDR. Data were log-transformed where appropriate and explicitly mentioned to ensure proper scaling and distributional assumptions. All software tools and version details are provided in the Methods section to ensure reproducibility. In box plots, boxes represent the interquartile range, and whiskers extend to 1.5× the interquartile range, with lower quartiles shaded darker and upper quartiles lighter.

### Reporting summary

Further information on research design is available in the Nature Portfolio Reporting Summary linked to this article.

## Online content

Any methods, additional references, Nature Portfolio reporting summaries, source data, extended data, supplementary information, acknowledgements, peer review information; details of author contributions and competing interests; and statements of data and code availability are available at https://doi.org/10.1038/s41588-026-02785-7.

## Supplementary information

Supplementary Information Supplementary Figs. 1–25.

Reporting Summary

Supplementary Tables Supplementary Tables 1–10.

## Source data

Source Data Fig. 1 Statistical source data.

Source Data Fig. 2 Statistical source data.

Source Data Fig. 3 Statistical source data.

Source Data Fig. 4 Statistical source data.

Source Data Fig. 5 Statistical source data.

Source Data Extended Data Fig. 2 Statistical source data.

Source Data Extended Data Fig. 3 Statistical source data.

Source Data Extended Data Fig. 4 Statistical source data.

Source Data Extended Data Fig. 5 Statistical source data.

Source Data Extended Data Fig. 6 Statistical source data.

## Data Availability

QTL summary statistics from this study are openly available via Zenodo at https://doi.org/10.5281/zenodo.20144650 (ref. ^174^). Individual-level data are available from QBB, now part of the Qatar Precision Health Institute (QPHI). Requests for data access should be submitted through the QPHI portal (https://www.qphi.org.qa/research/how-to-apply; accession ID: QPHI-RES-FO-009) and are subject to review by the QPHI Access Committee and QBB Institutional Review Board. Previously published data used in this study are accessible as follows: ClinVar database v2023-10-01 (ref. ^49^) (https://www.ncbi.nlm.nih.gov/clinvar/), eQTL Catalogue^122^ (https://www.ebi.ac.uk/eqtl/Data_access/), eQTLGen^20^ (https://www.eqtlgen.org/cis-eqtls.html), GALA II/SAGE^21^ (https://zenodo.org/record/7735723), GTEx V8 (ref. ^19^) (https://gtexportal.org/home/downloads/adult-gtex/qtl), GWAS summary statistics^58^ (https://www.ebi.ac.uk/gwas/publications/33623009), NCBI UniVec database^143^ (www.ncbi.nlm.nih.gov/tools/vecscreen/univec), QBB^37^ (https://www.qphi.org.qa/) and Sardinian QTL^17^ (http://eqtlsdownload.irgb.cnr.it/). Source data are provided with this paper.

## Code availability

The study used previously published analysis tools as described in the Methods section. No custom tools were used.
