# Supplementary material for: A catalog of transcriptome genetic regulation in a consanguineous Middle Eastern biobank
Source: Nat Genet. 2026 Nov 2;58(11):2832–44. doi: 10.1038/s41588-026-02785-7 (PMC13754583; doi:10.1038/s41588-026-02785-7)

nature genetics

Supplementary information

https://doi.org/10.1038/s41588-026-02785-7

# A catalog of transcriptome genetic regulation in a consanguineous Middle Eastern biobank

In the format provided by the authors and unedited

# Contents

Supplementary Figures

**Supplementary Fig. 1. Yield, RNA quality score (RQN), Sequencing depth and Preprocessing for the Study Samples. (a)** Histogram of RNA yield obtained for the study samples. **(b)** Histogram of RNA Quality Number (RQN). **(c)** Count of raw reads (R1) for the sequenced samples at either medium or high depth. **(d)** The number reads for the sequenced samples at various QC and filtering steps. The left panel displays the absolute counts, while the right panel shows the percentage drop relative to the initial raw reads count. Dot indicates individual samples and are shown in distinct colors.

a

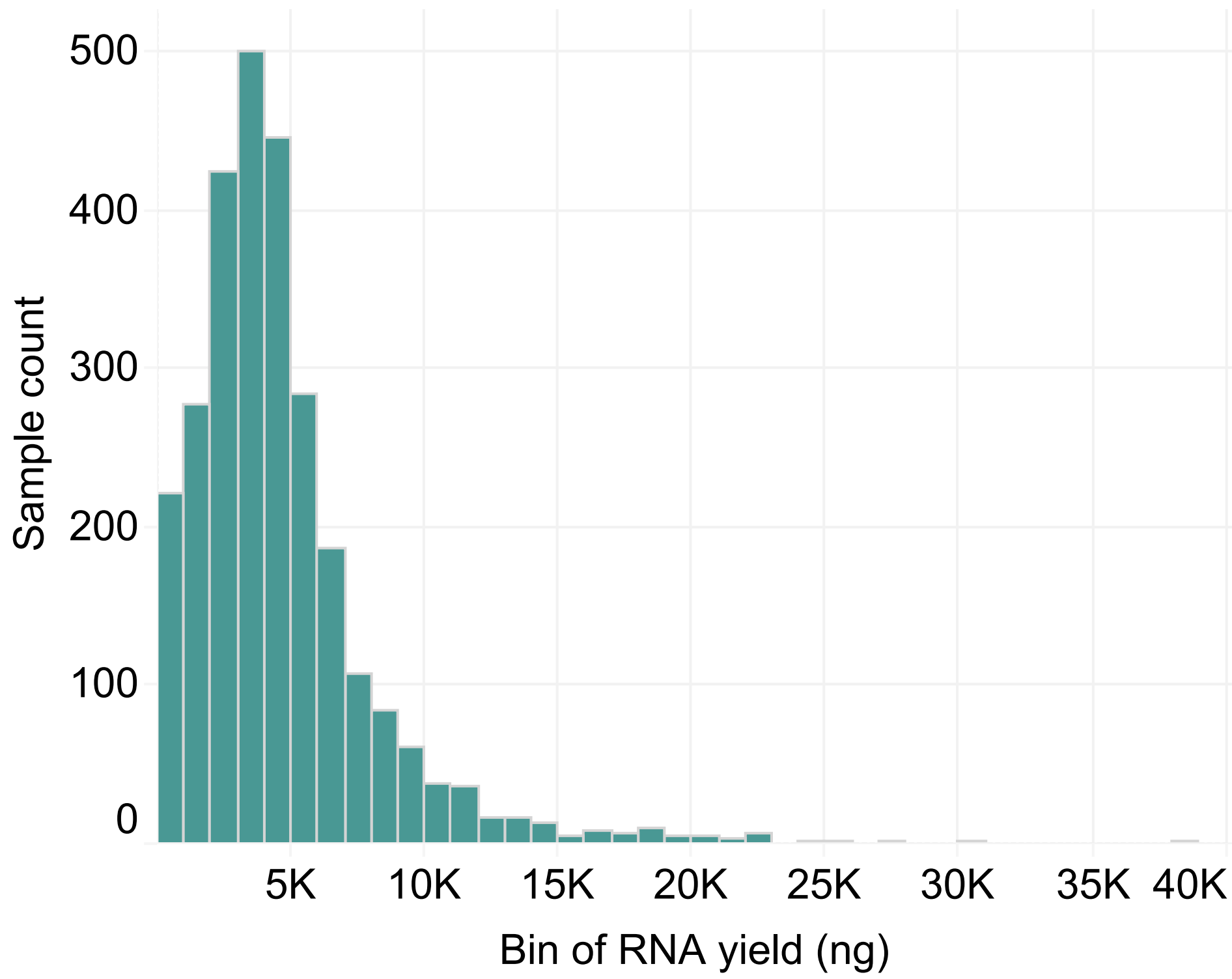


b

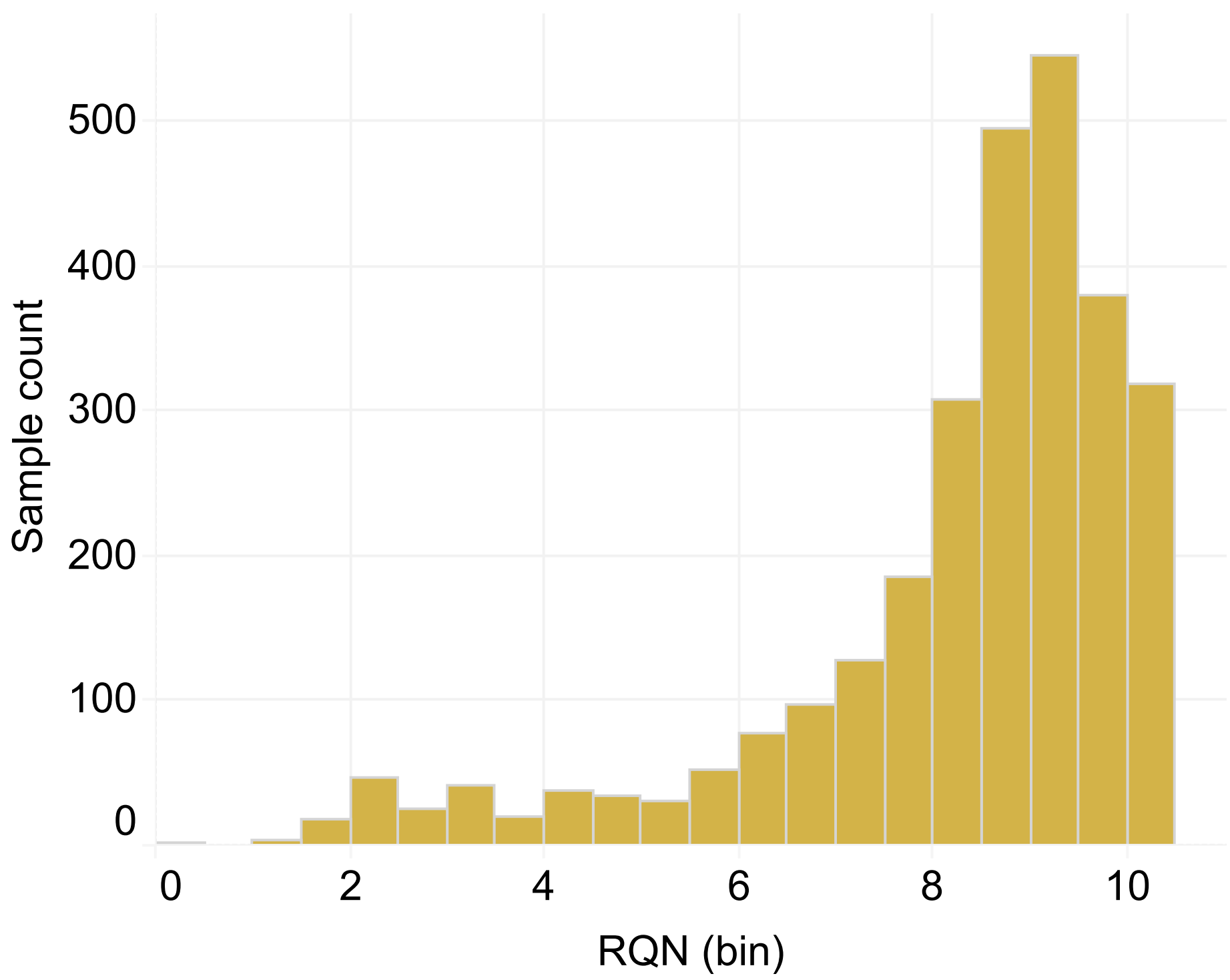

c

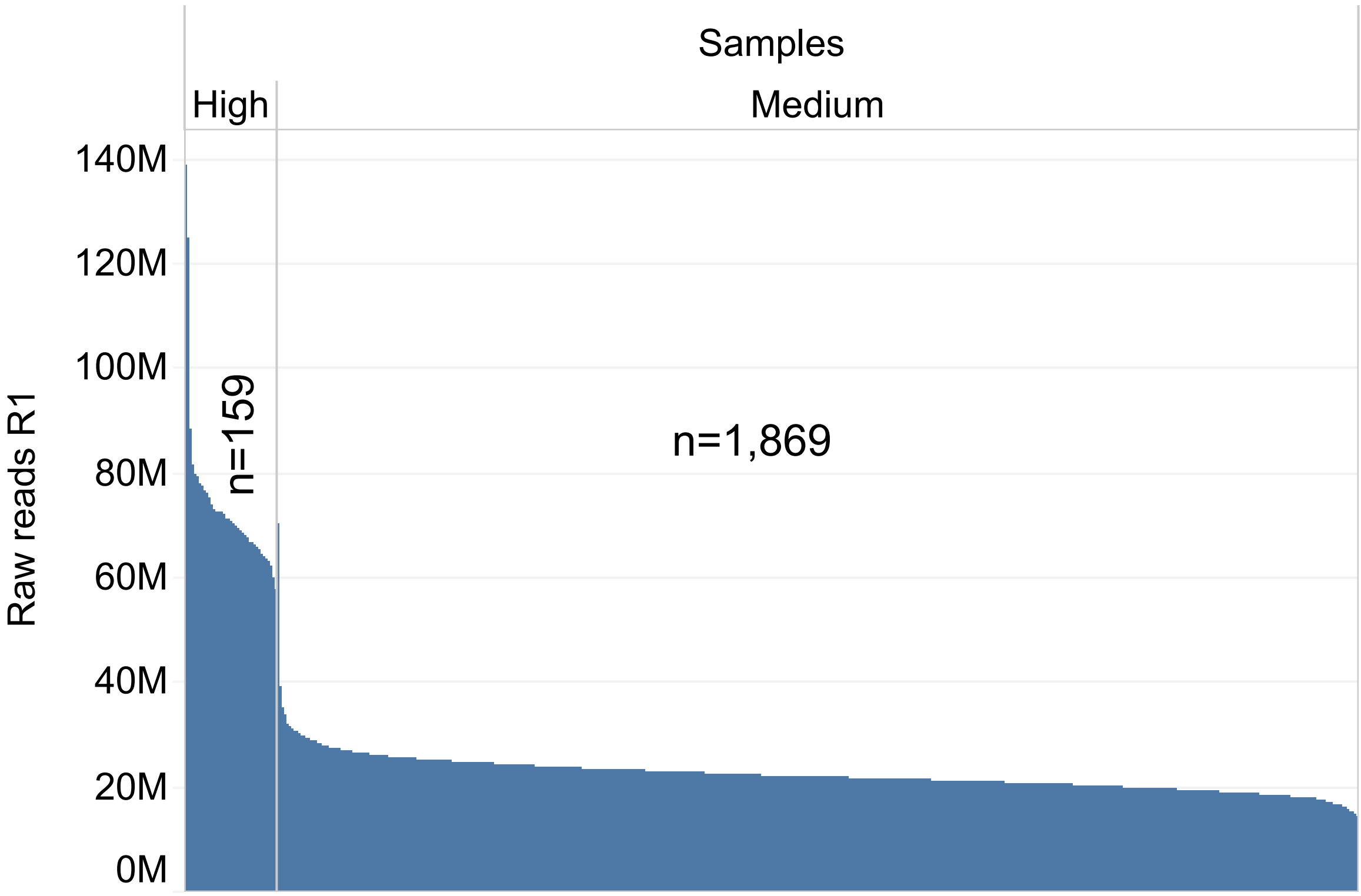
Samples
High
Medium
140M
120M
100M
80M
60M
40M
20M
0M
Raw reads R1
n=159
n=1,869

d

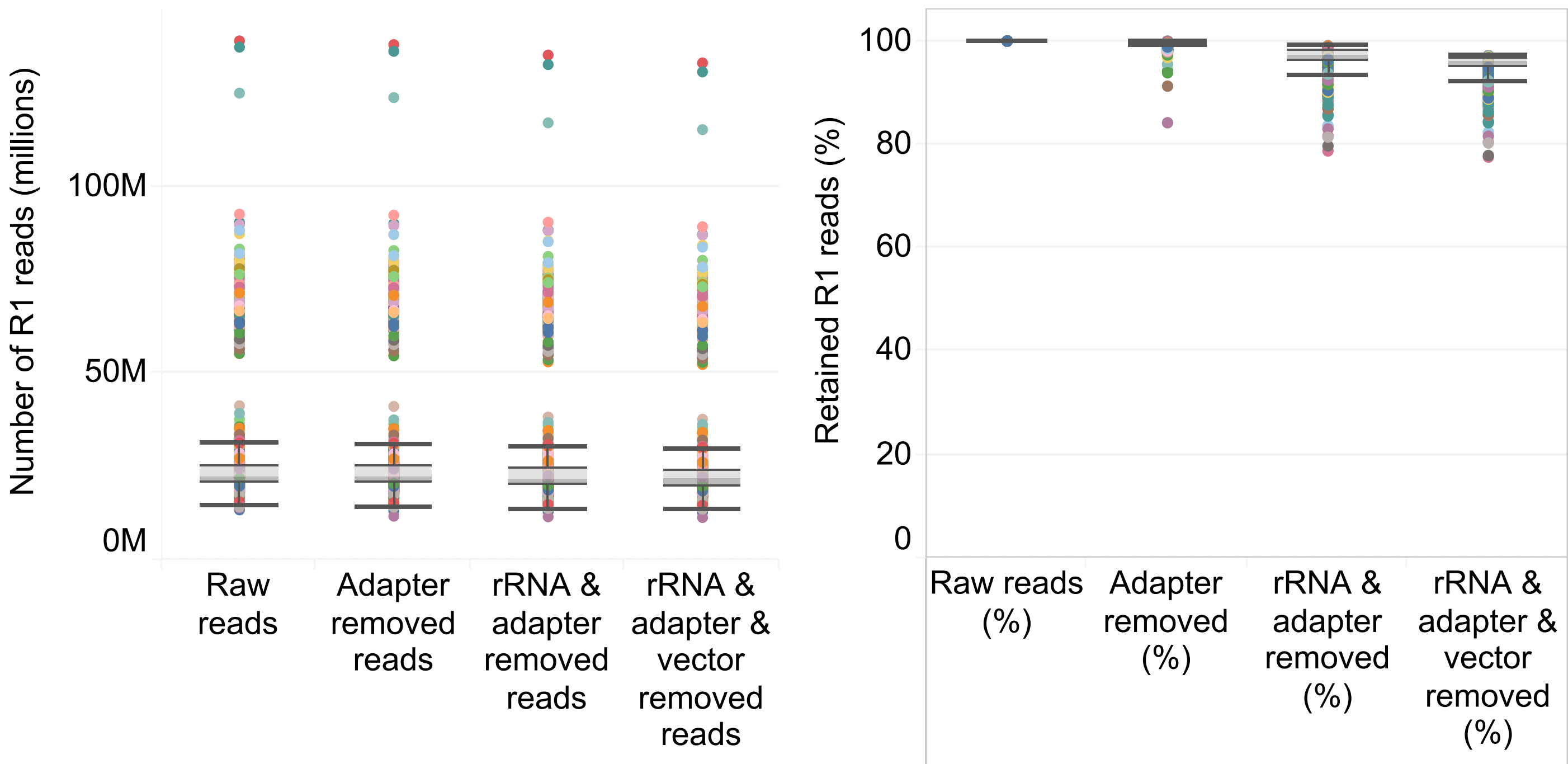
100M
50M
0M
Number of R1 reads (millions)
Raw reads
Adapter removed reads
rRNA & adapter removed reads
rRNA & adapter & vector removed reads
100
80
60
40
20
0
Retained R1 reads (%)
Raw reads (%)
Adapter removed (%)
rRNA & adapter removed (%)
rRNA & adapter & vector removed (%)

**Supplementary Fig. 2. Sample-level Matching of WGS and RNAseq Data for the Sequenced Cohort.** Scatter plot of Freemix versus Chipmix scores. Each dot is one sample. Colour gradient represents IBD (identity-by-descent) scores, indicating genetic similarity. Dashed lines show the recommended minimal thresholds for contamination.

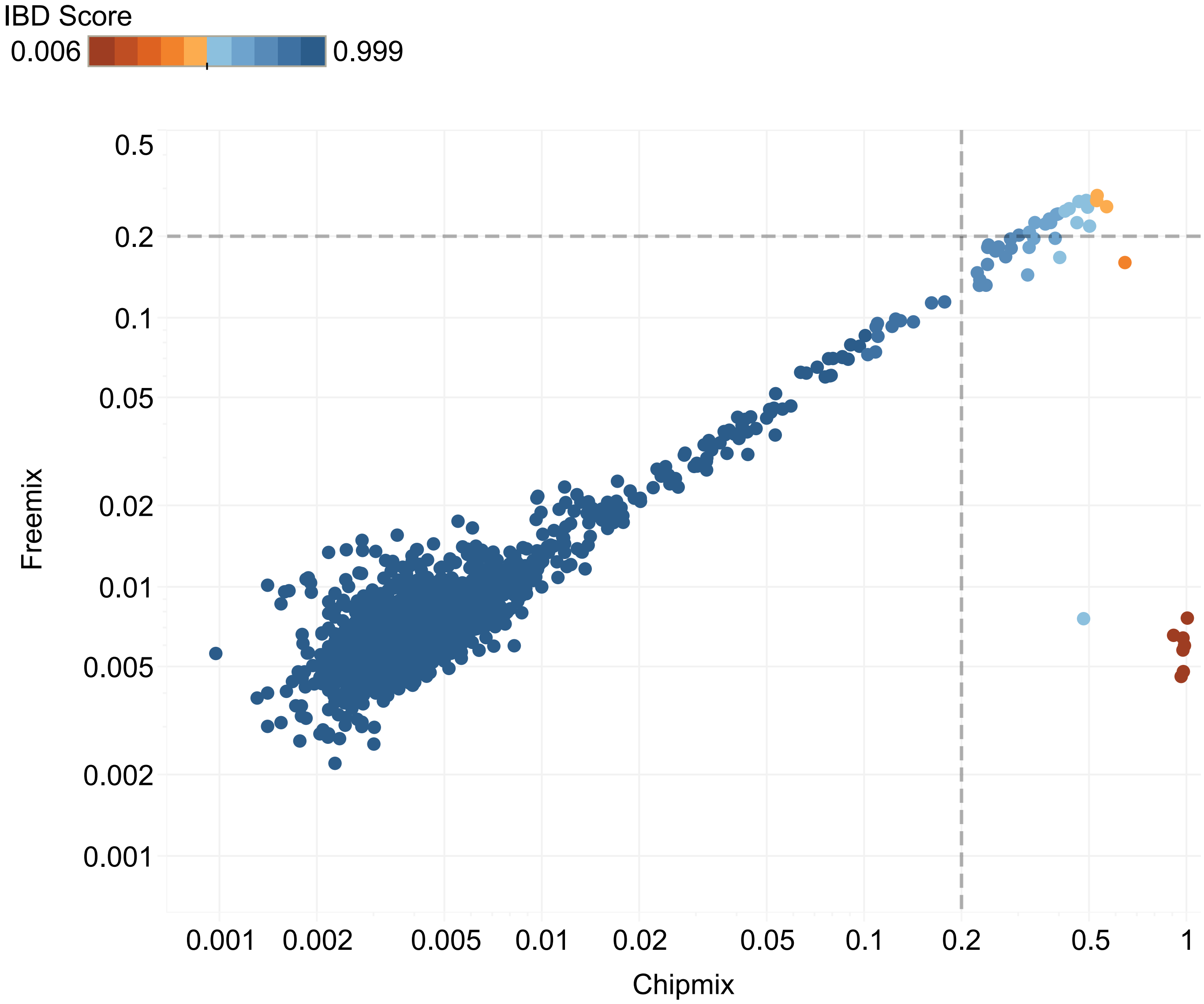

**Supplementary Fig. 3. eQTL Discovery as a Function of Cumulative PEER Factor Covariates.** Count of significant eQTLs (FDR < 0.05) detected using an increasing number of PEER factors as covariates, based on a random set of 1,500 genes, interval of 5 and six replicates. The vertical line at n = 45 indicates the selected number of PEER factors for downstream analysis.

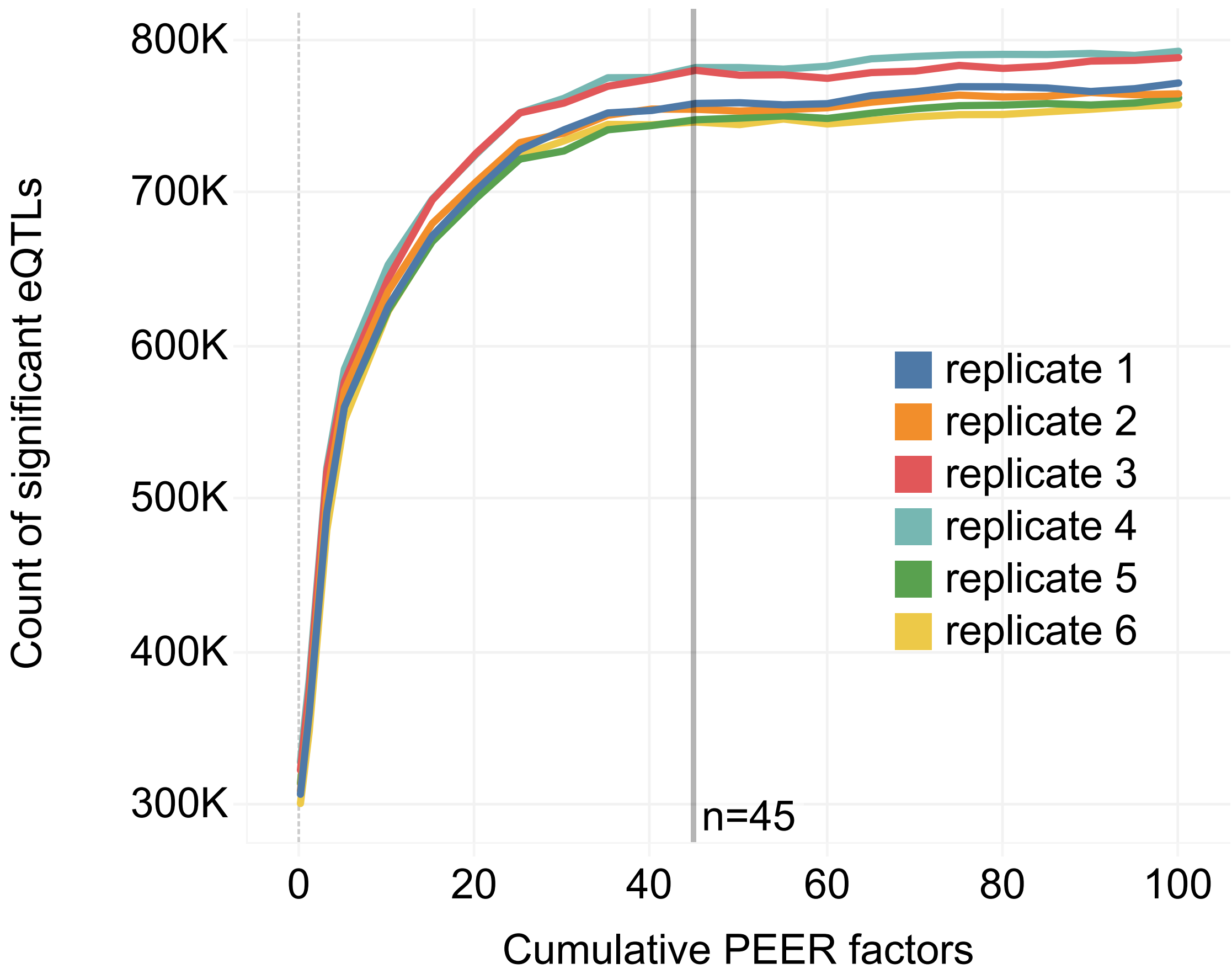

**Supplementary Fig. 4. Correlation of Covariates Across Study Samples.** Heatmap of Pearson correlation coefficients *(r)* between various compiled and calculated PEER factor covariates.

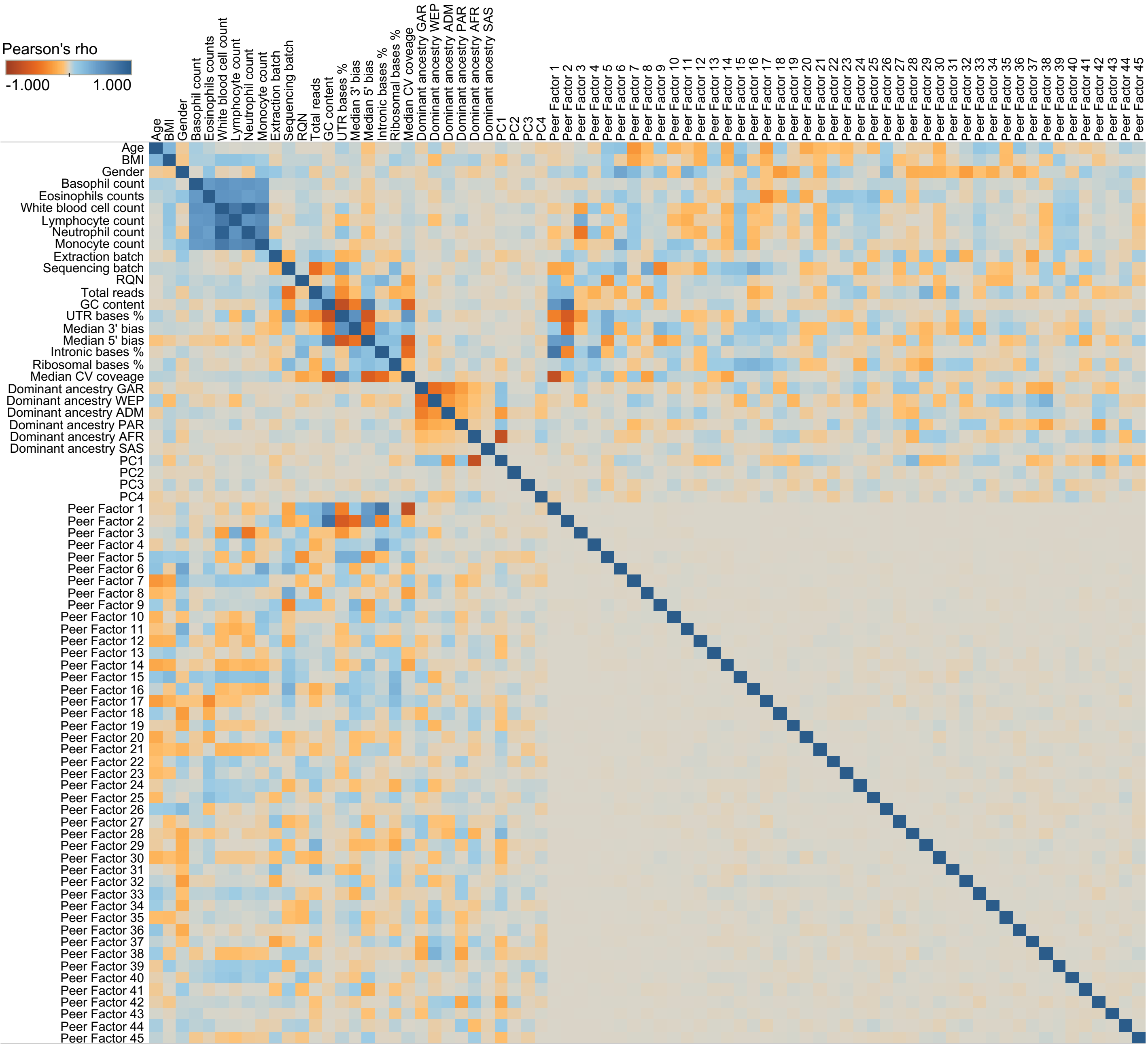

**Supplementary Fig. 5. Pipelines of Univariate QTL Discovery.** Flowcharts are shown for the various steps involved in calculating **(a)** eQTLs, **(b)** isoQTLs, **(c)** sQTLs, **(d)** aseQTL (v) and **(e)** aseQTL (g).

a

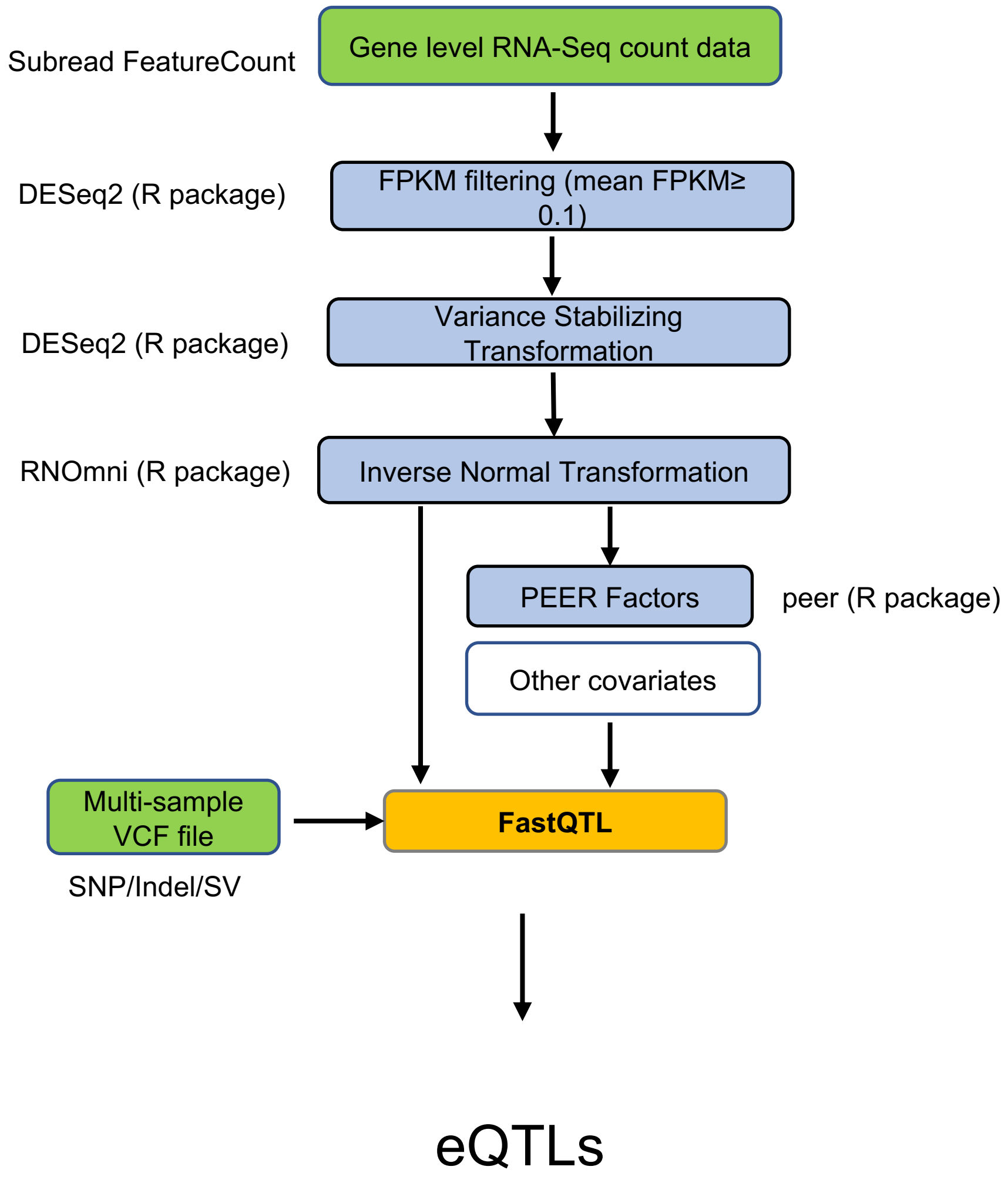

b

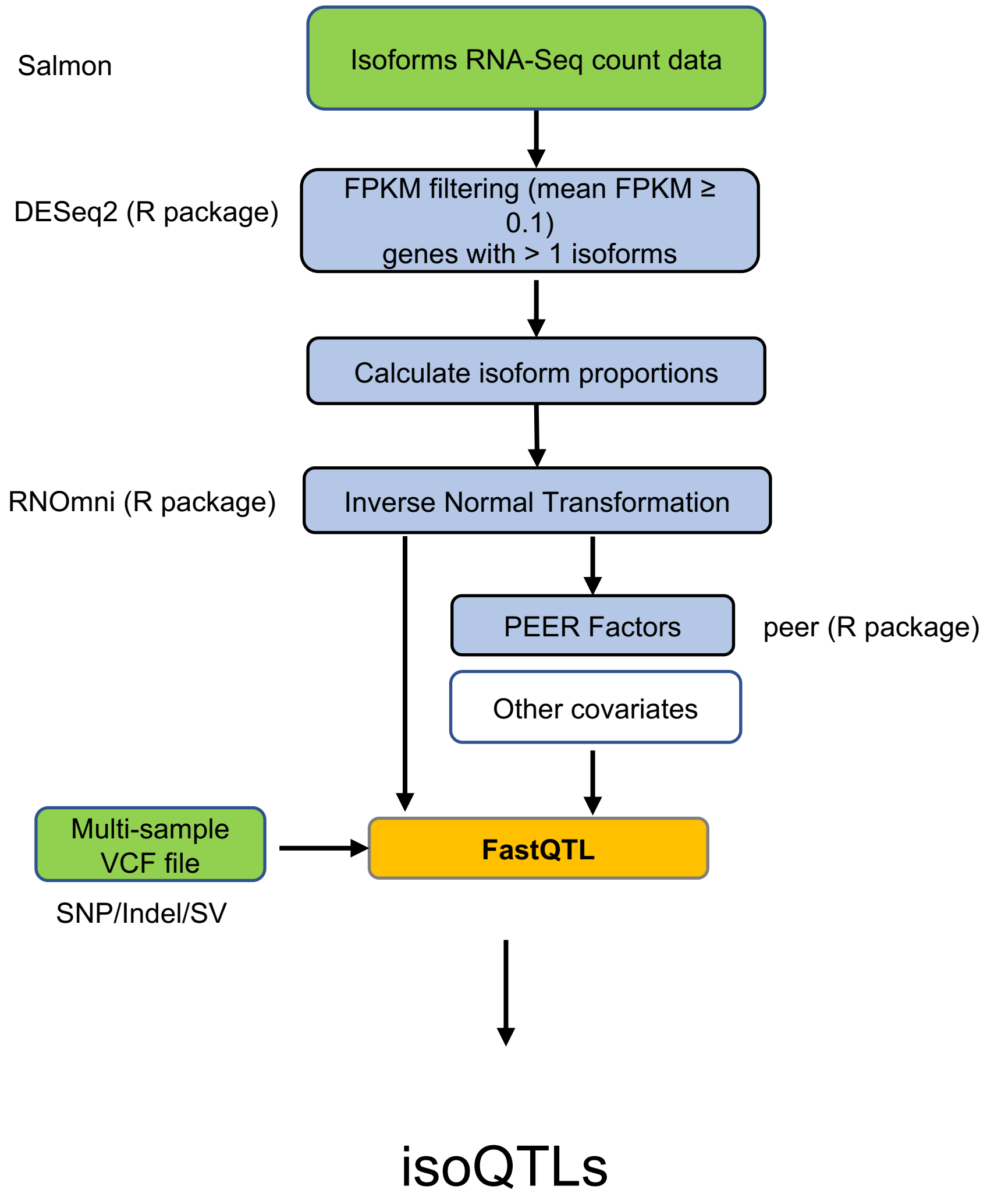
Salmon
Isoforms RNA-Seq count data
DESeq2 (R package)
FPKM filtering (mean FPKM ≥ 0.1)
genes with > 1 isoforms
Calculate isoform proportions
RNOmni (R package)
Inverse Normal Transformation
PEER Factors
peer (R package)
Other covariates
Multi-sample VCF file
SNP/Indel/SV
FastQTL
isoQTLs

c

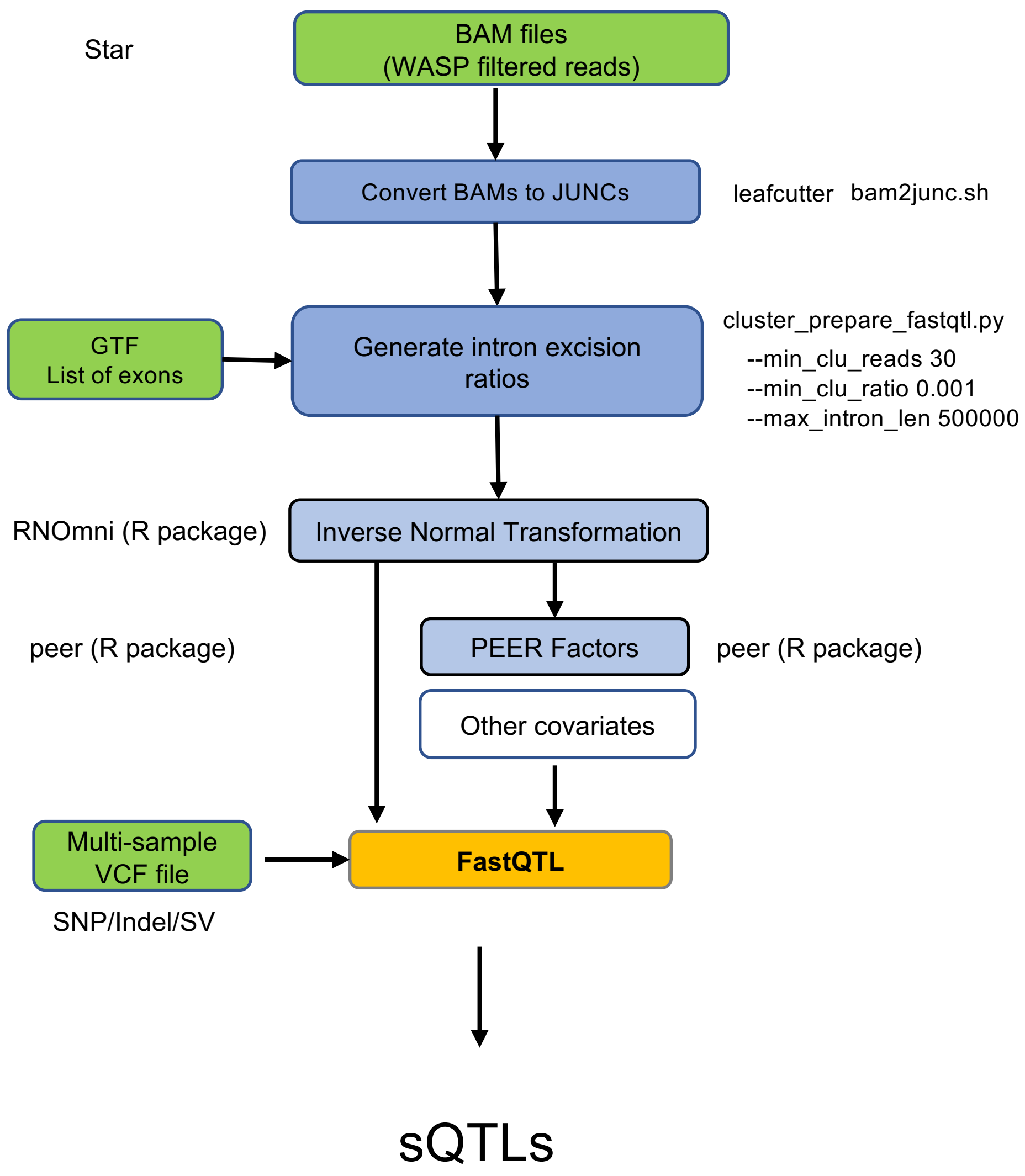
Star
BAM files
(WASP filtered reads)
Convert BAMs to JUNCs
leafcutter bam2junc.sh
GTF
List of exons
Generate intron excision ratios
cluster_prepare_fastqtl.py
--min_clu_reads 30
--min_clu_ratio 0.001
--max_intron_len 500000
RNOmni (R package)
Inverse Normal Transformation
peer (R package)
PEER Factors
peer (R package)
Other covariates
Multi-sample
VCF file
SNP/Indel/SV
FastQTL
sQTLs

d

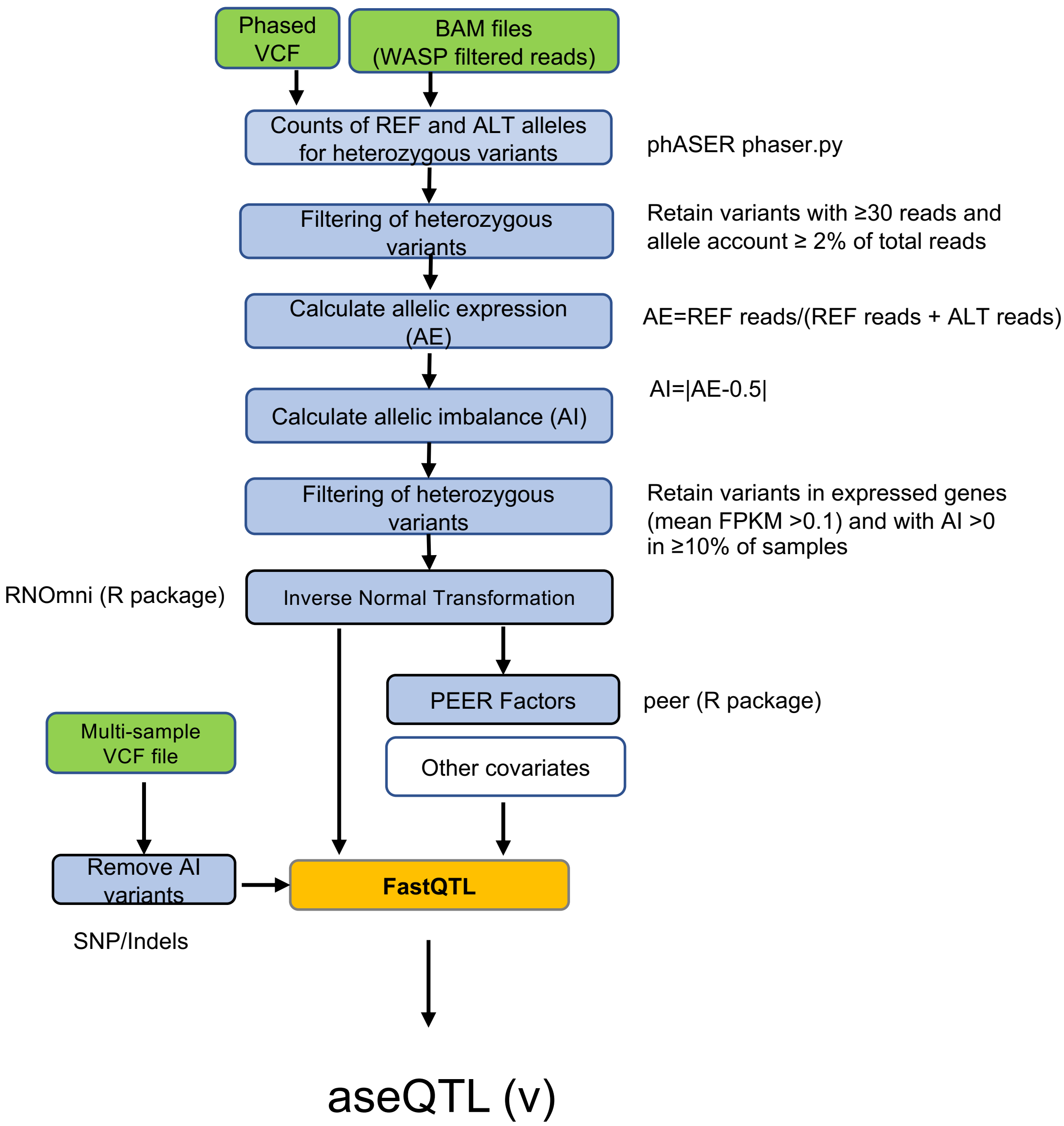
Phased VCF
BAM files (WASP filtered reads)
Counts of REF and ALT alleles for heterozygous variants
phASER phaser.py
Filtering of heterozygous variants
Retain variants with ≥30 reads and allele account ≥ 2% of total reads
Calculate allelic expression (AE)
AE=REF reads/(REF reads + ALT reads)
Calculate allelic imbalance (AI)
AI=|AE-0.5|
Filtering of heterozygous variants
Retain variants in expressed genes (mean FPKM >0.1) and with AI >0 in ≥10% of samples
RNOmni (R package)
Inverse Normal Transformation
PEER Factors
peer (R package)
Other covariates
Multi-sample VCF file
Remove AI variants
SNP/Indels
FastQTL
aseQTL (v)

e

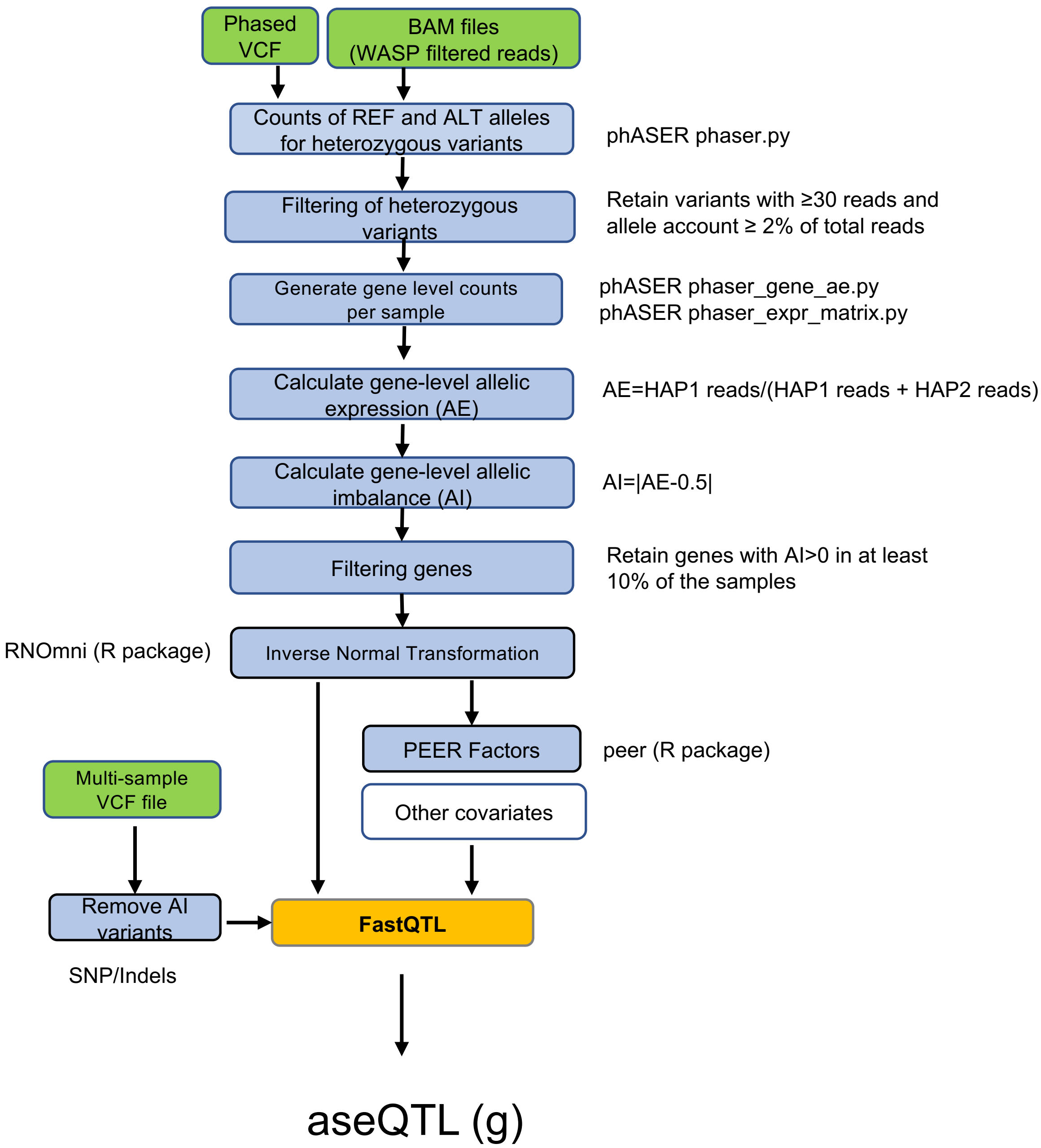

Phased VCF
BAM files (WASP filtered reads)
Counts of REF and ALT alleles for heterozygous variants
phASER phaser.py
Filtering of heterozygous variants
Retain variants with ≥30 reads and allele account ≥ 2% of total reads
Generate gene level counts per sample
phASER phaser_gene_ae.py
phASER phaser_expr_matrix.py
Calculate gene-level allelic expression (AE)
AE=HAP1 reads/(HAP1 reads + HAP2 reads)
Calculate gene-level allelic imbalance (AI)
AI=|AE-0.5|
Filtering genes
Retain genes with AI>0 in at least 10% of the samples
RNOmni (R package)
Inverse Normal Transformation
PEER Factors
peer (R package)
Multi-sample VCF file
Other covariates
Remove AI variants
FastQTL
SNP/Indels
aseQTL (g)

**Supplementary Fig. 6. Quantile-Quantile plots for various QTL analyses.** Each dot delineates the expected versus observed p-values for a given QTL-feature pair in this study. Red line indicates unity line. The lowest p-value observed reflect the limits of machine precision.

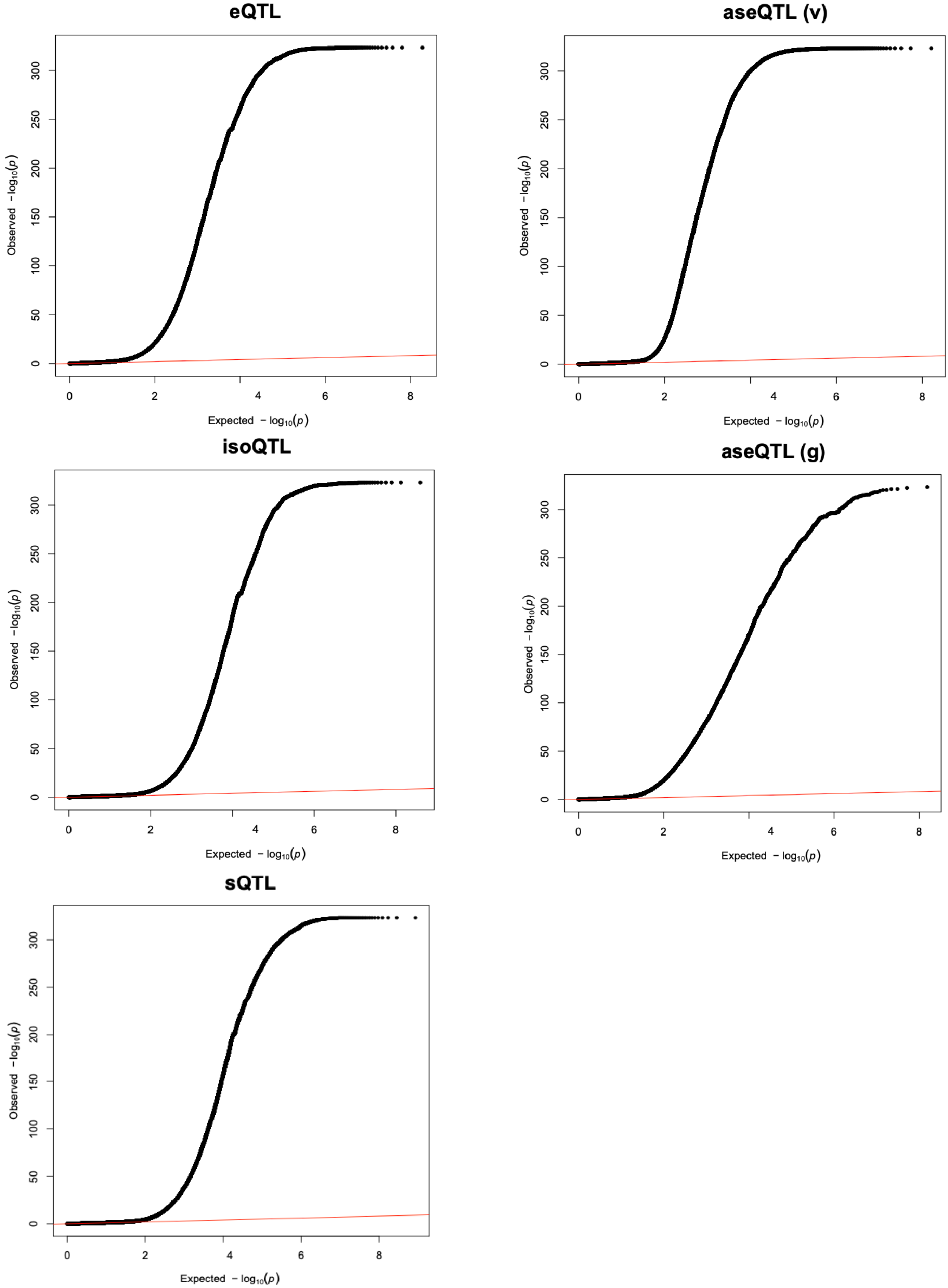

**Supplementary Fig. 7. Genomic Distribution of Univariate QTLs. (a)** Density of QTLs as a function of distance from transcription start sites (TSS). **(b)** Cumulative percentage of QTL pairs as a function of absolute distance from TSS. **(c)** Log count and enrichment of univariate QTLs across genomic annotations and impact predicted by VEP. Enrichment is calculated as the log ratio of observed to expected counts, both normalised for baseline variant count. Full circles denote statistically-significant enrichment (>0) or depletion (<0) at Bonferroni-adjusted p-value < 0.05, while open circles denote non-significant values.

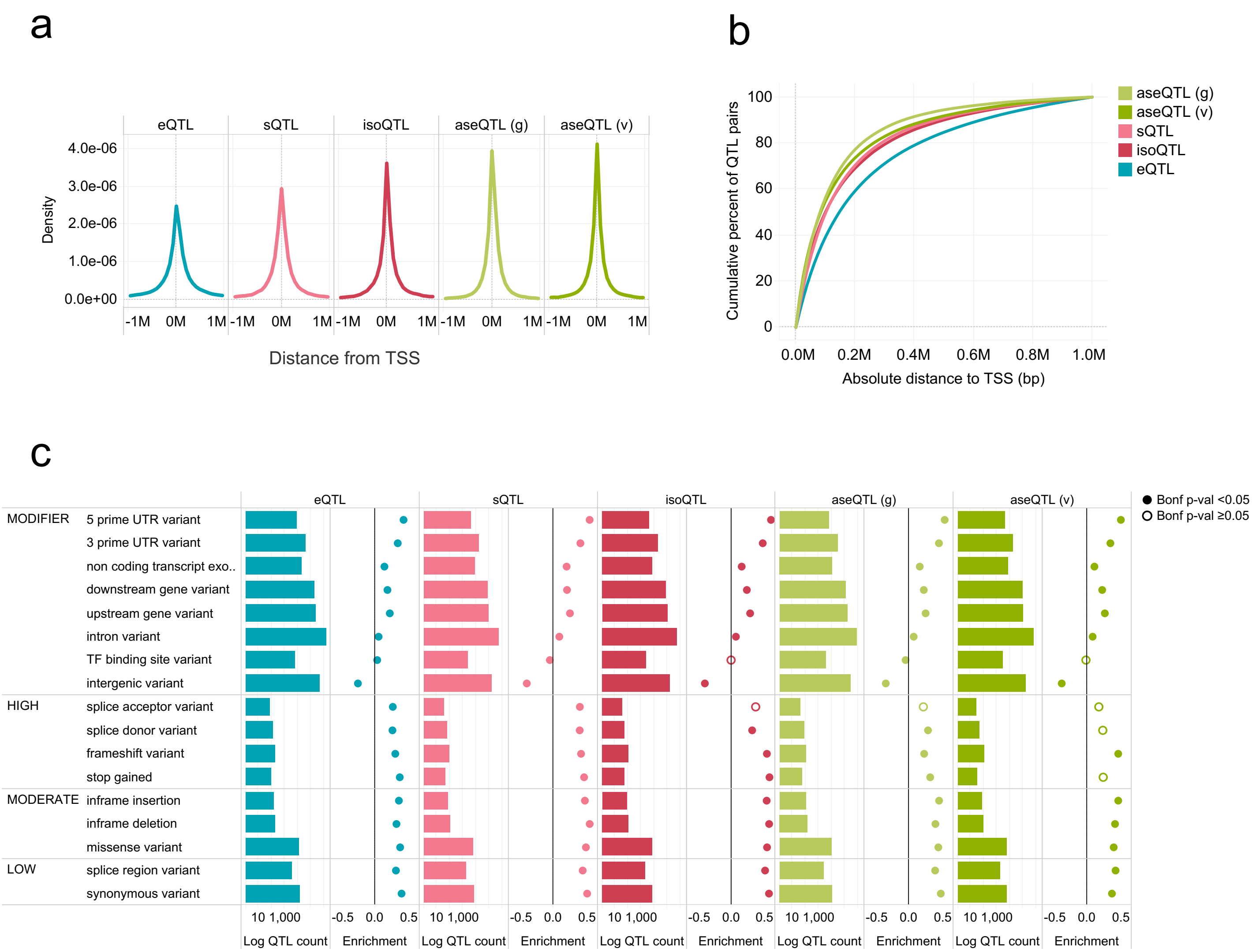

**Supplementary Fig. 8. Percentage of Univariate QTLs Associated with a Given Number of Genes.** Corresponding counts are shown on each bar.

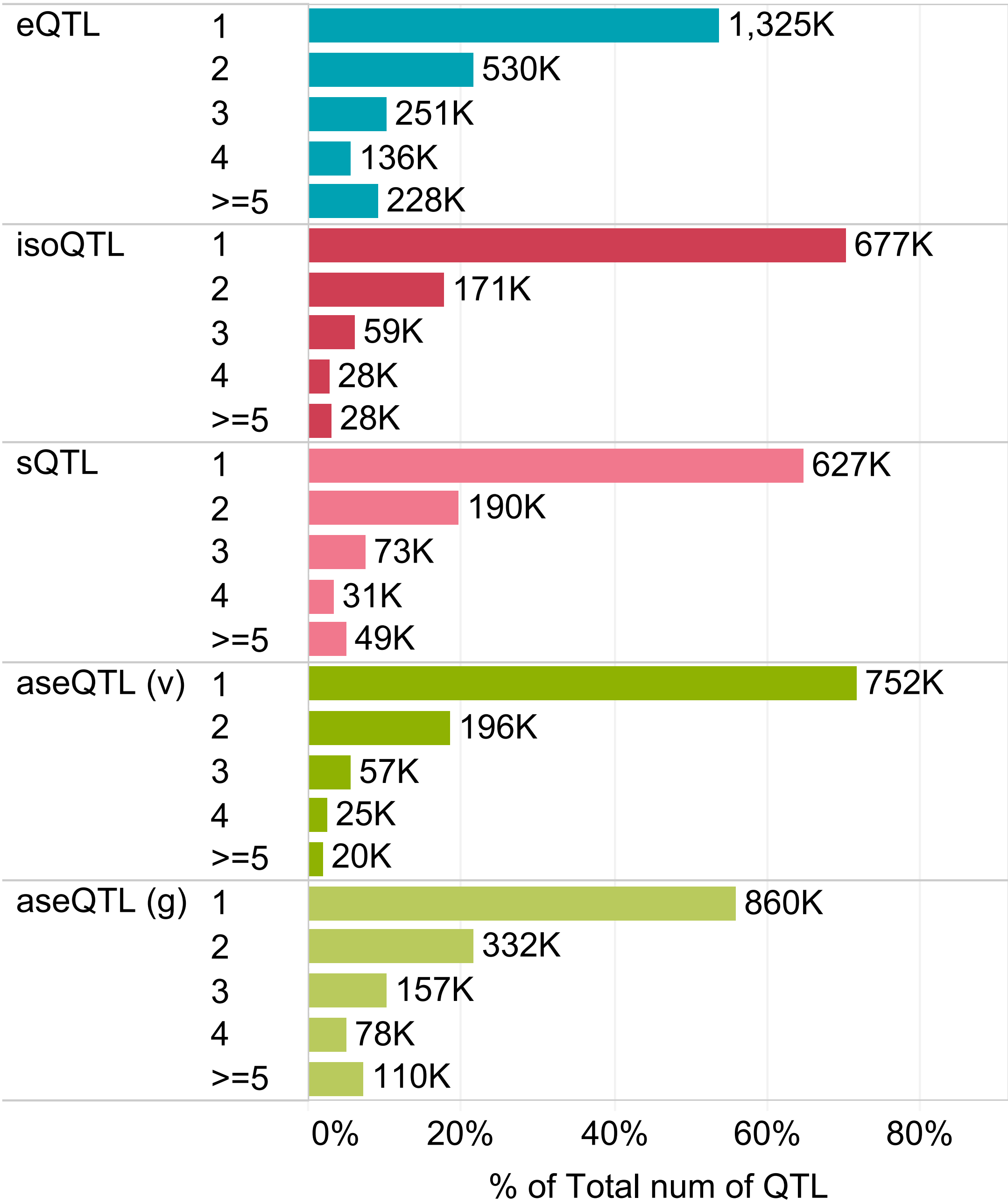

**Supplementary Fig. 9. Biotype of Univariate QTL Genes.** Percentage and count of QTL genes across various functional gene classes.

| | | eQTL | isoQTL | sQTL | aseQTL (v) | aseQTL (g) |
|---|---|---|---|---|---|---|
| Protein coding | protein_coding | 9,374 | 4,785 | 4,917 | 3,830 | 9,210 |
| lncRNA | antisense | 1,061 | | 218 | 162 | 889 |
| | lincRNA | 799 | | 202 | 65 | 557 |
| | sense_overlapping | 47 | | 4 | 6 | 46 |
| | sense_intronic | 3 | | 15 | | 1 |
| | 3prime_overlapping_ncrna | 4 | | 2 | 2 | 3 |
| ncRNA | processed_transcript | 106 | | 104 | 27 | 98 |
| | miRNA | 66 | | | 1 | 6 |
| | snRNA | 41 | | | | 3 |
| | misc_RNA | 27 | | | | 2 |
| | snoRNA | 14 | | | | 2 |
| | rRNA | 2 | | | | 1 |
| Pseudogene | pseudogene | 927 | 122 | 163 | 25 | 270 |
| | IG_V_pseudogene | 20 | 1 | 1 | | 4 |
| | polymorphic_pseudogene | 6 | 4 | 5 | 2 | 5 |
| | TR_V_pseudogene | 11 | | 1 | | 5 |
| | IG_C_pseudogene | 4 | | 1 | | |
| | IG_J_pseudogene | 3 | | | | |
| | TR_J_pseudogene | 1 | | | | |
| IG gene | IG_V_gene | 114 | 1 | 12 | 9 | 23 |
| | IG_C_gene | 12 | 1 | 11 | 4 | 3 |
| | IG_J_gene | 11 | | 15 | 1 | 1 |
| | IG_D_gene | 19 | | | | 1 |
| TR gene | TR_V_gene | 84 | | 17 | 13 | 45 |
| | TR_J_gene | 44 | | 44 | | 9 |
| | TR_C_gene | 5 | | 5 | 5 | 5 |
| | TR_D_gene | 1 | | | | |
| | | 0% 100% QTL genes (%) | 0% 100% QTL genes (%) | 0% 100% QTL genes (%) | 0% 100% QTL genes (%) | 0% 100% QTL genes (%) |

**Supplementary Fig. 10. Overlap of QBB/QGP Univariate QTLs with Public Datasets. (a)** UpSet plot of eQTL and eQTL-gene pairs. Intersections are colored with a gradient based on the counts. **(b)** Analogous Venn diagrams for sQTL, isoQTL, and aseQTL (v).

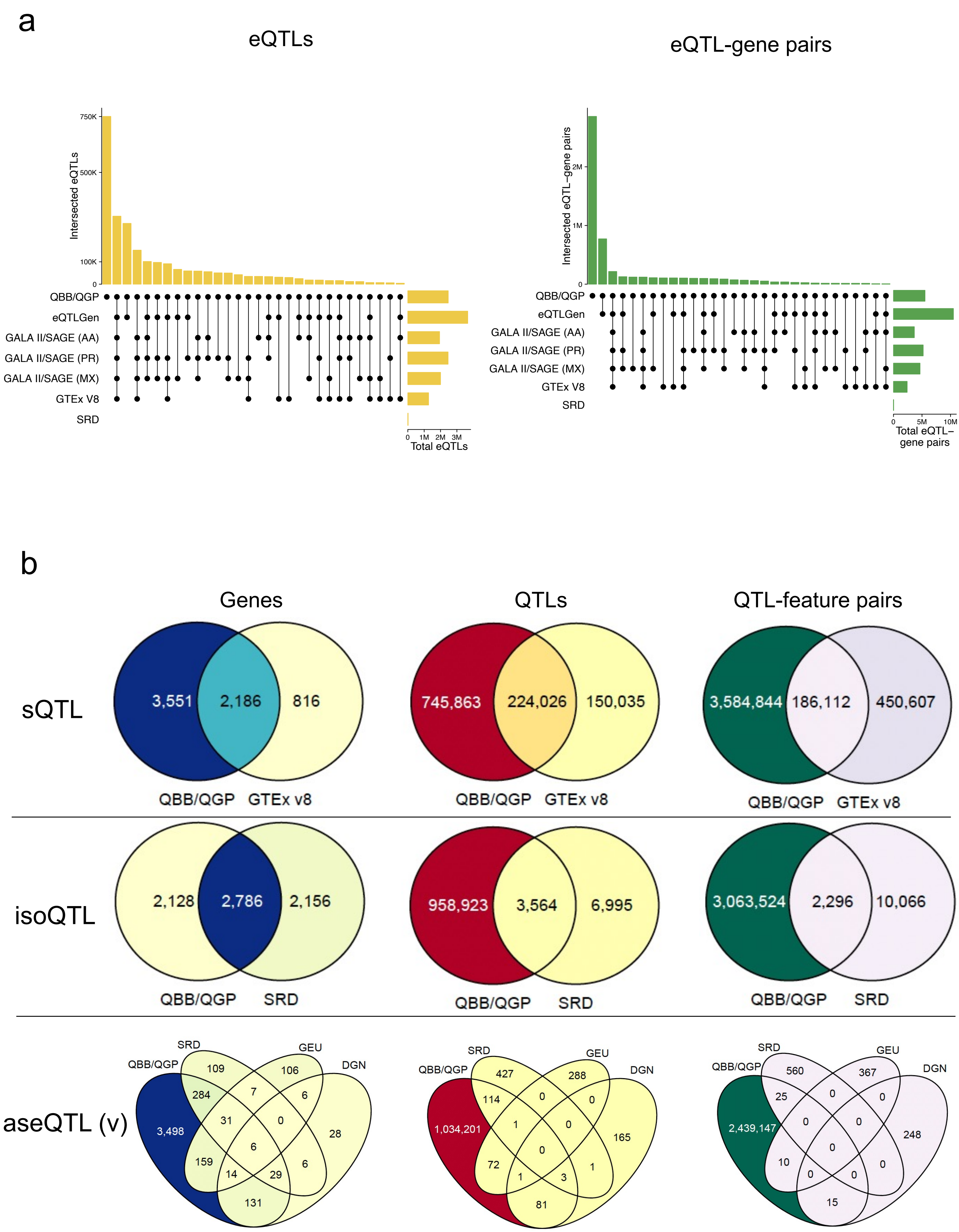

**Supplementary Fig. 11. Pipeline for QTL Fine Mapping.** Flowchart indicates the main steps used in the implemented SuSiE workflow.

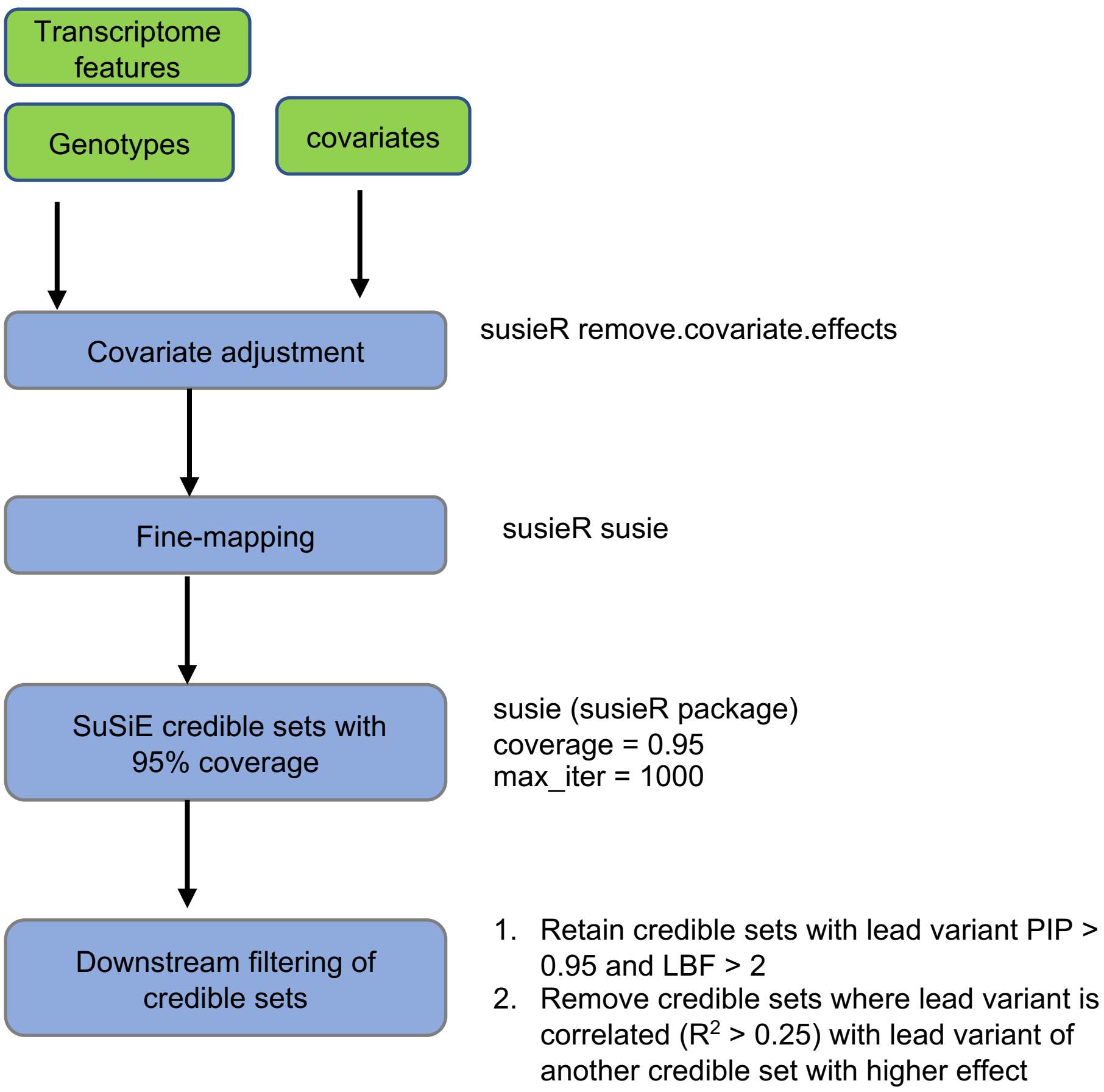

**Supplementary Fig. 12. Relationship Between Causal QTL Effect Size, Log Bayes Factor, PIP, Slope (β) and MAF. (a)** Volcano plots of causal QTL effect size (μ) versus log Bayes factors (LBF) per QTL type, coloured by z-score. **(b)** Scatter plots of causal QTL effect size (μ) versus Posterior Inclusion Probability (PIP) per QTL type, coloured by z-score. **(c)** Minor allele frequency (MAF) versus absolute slope (β). For visualization, causal QTLs with an absolute fine-mapping effect size (μ) > 0.2 are shown. **(d)** β versus μ for fine-mapped QTLs. Opposite direction effects are rare (<0.69% of QTL pairs) in line with expectations.

a

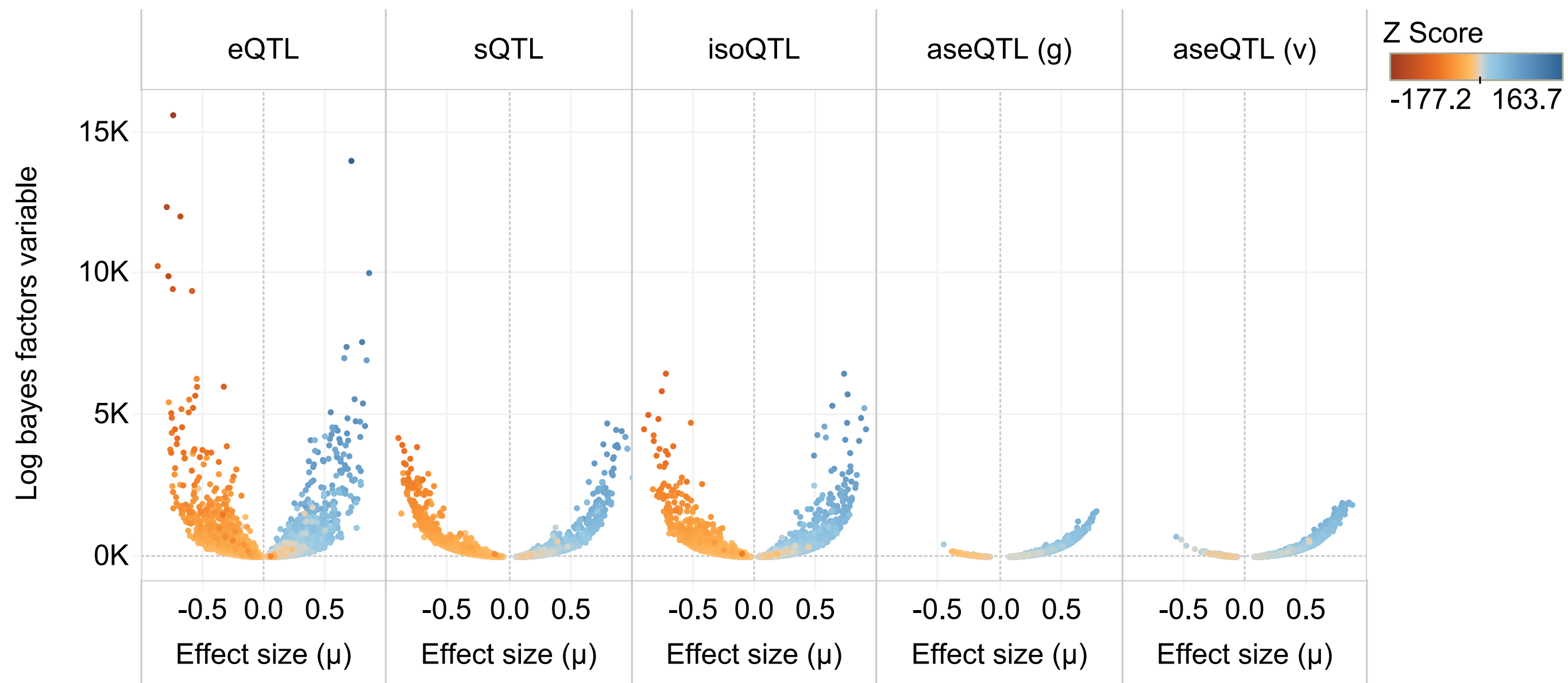


b

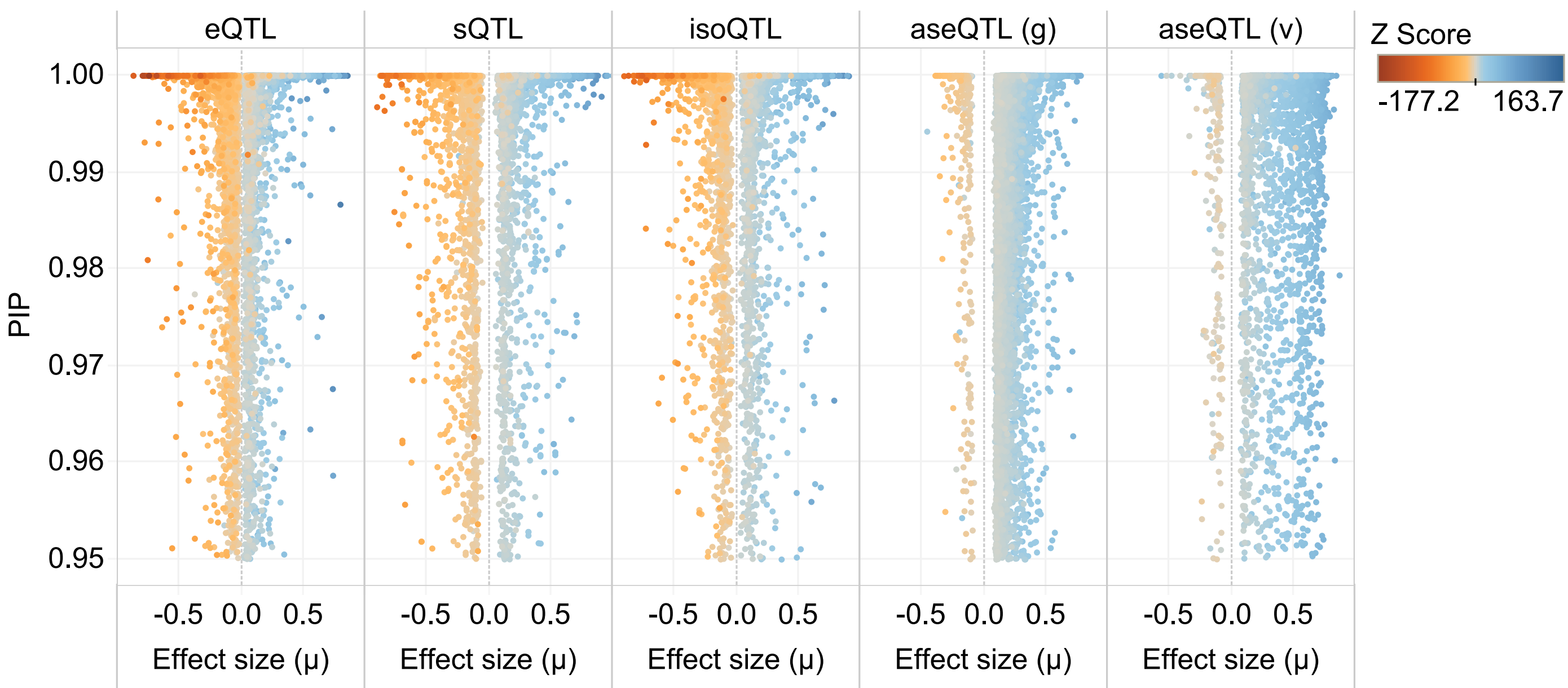

c

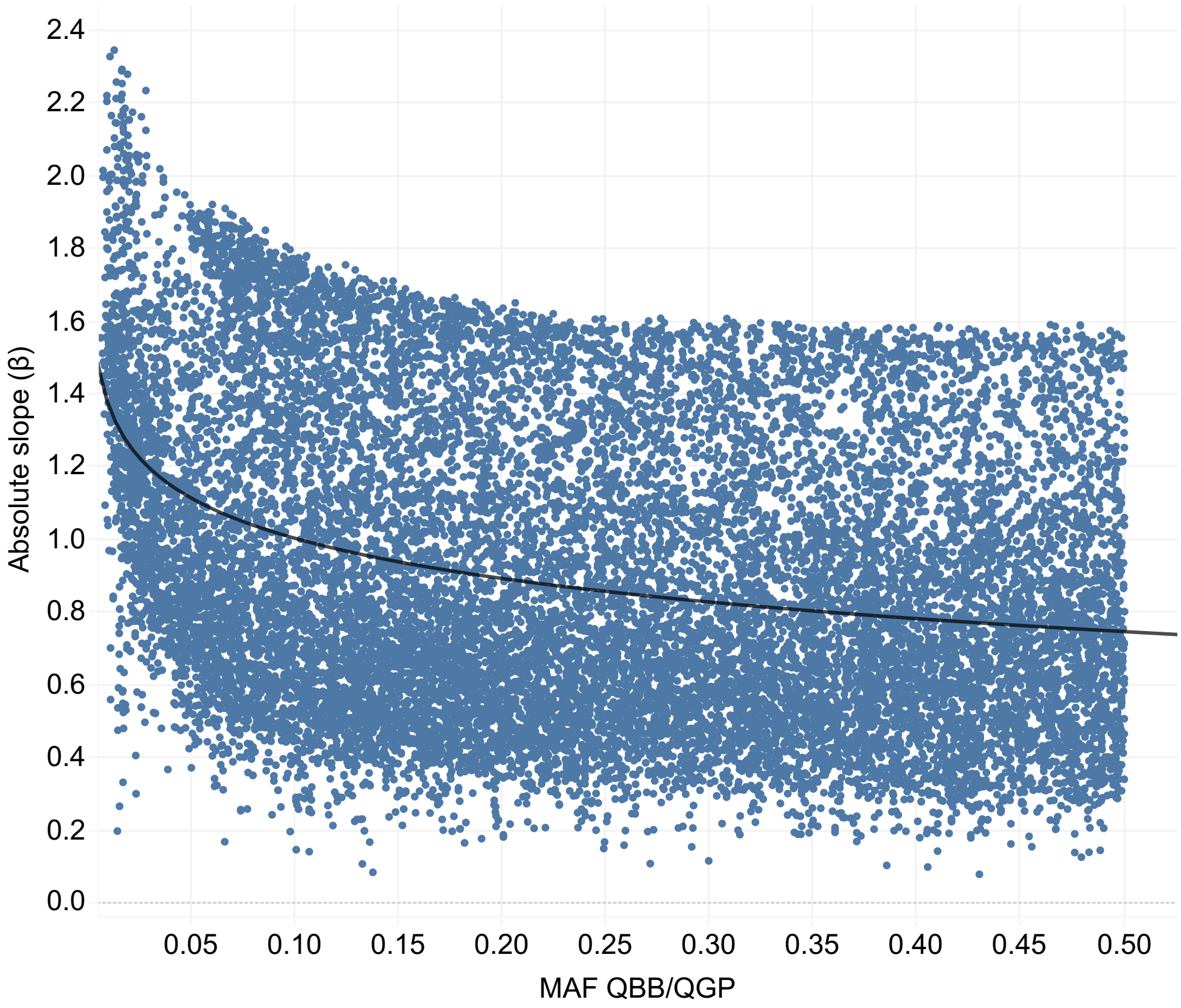

2.4
2.2
2.0
1.8
1.6
1.4
1.2
1.0
0.8
0.6
0.4
0.2
0.0
Absolute slope (β)
0.05
0.10
0.15
0.20
0.25
0.30
0.35
0.40
0.45
0.50
MAF QBB/QGP


d

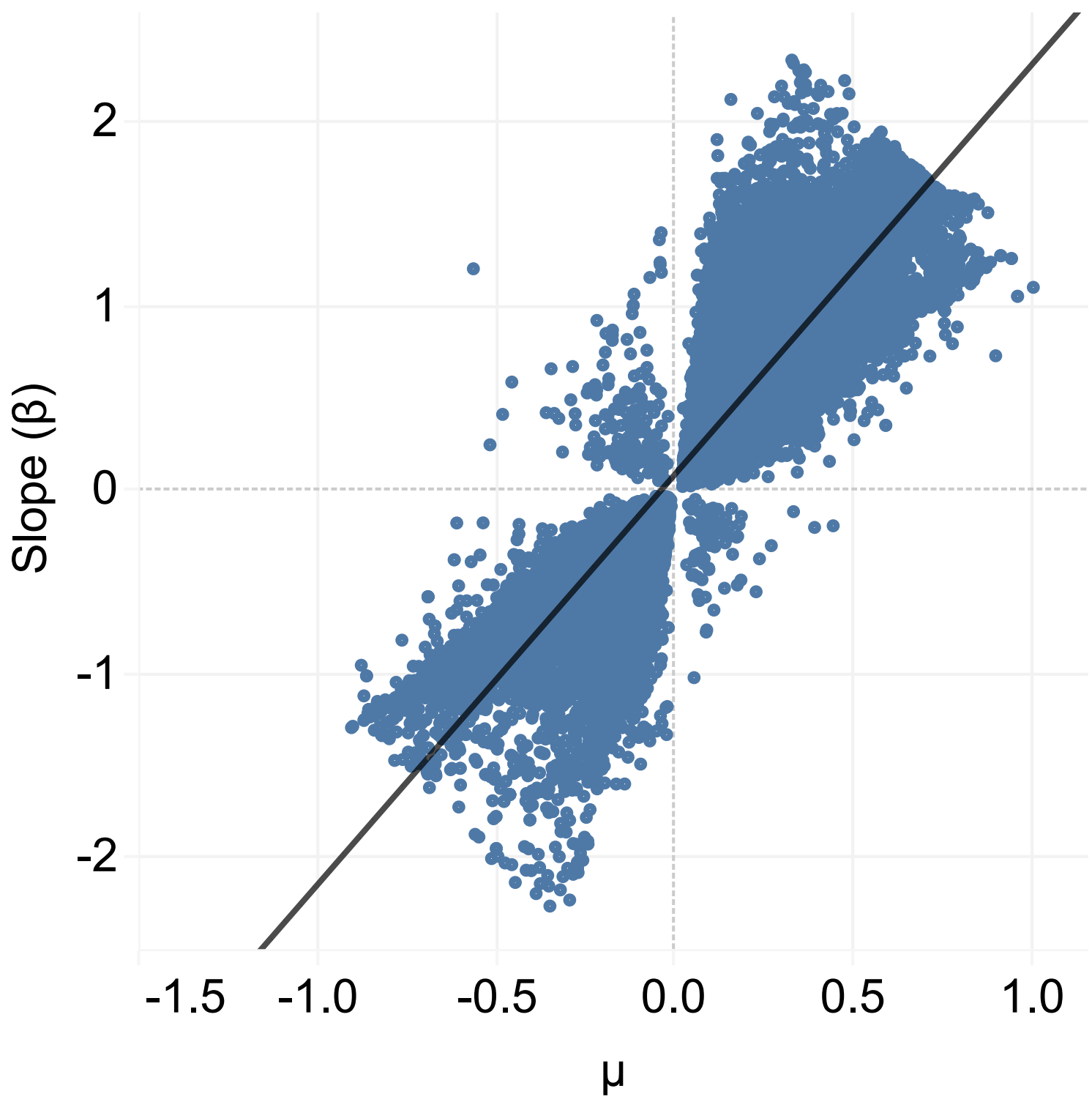

2
1
0
-1
-2
Slope (β)
-1.5
-1.0
-0.5
0.0
0.5
1.0
μ

**Supplementary Fig. 13. Count of Causal QTLs, Features and Genes.** Count of causal QTLs, transcriptome trait features, and genes identified in this study for various QTL types.

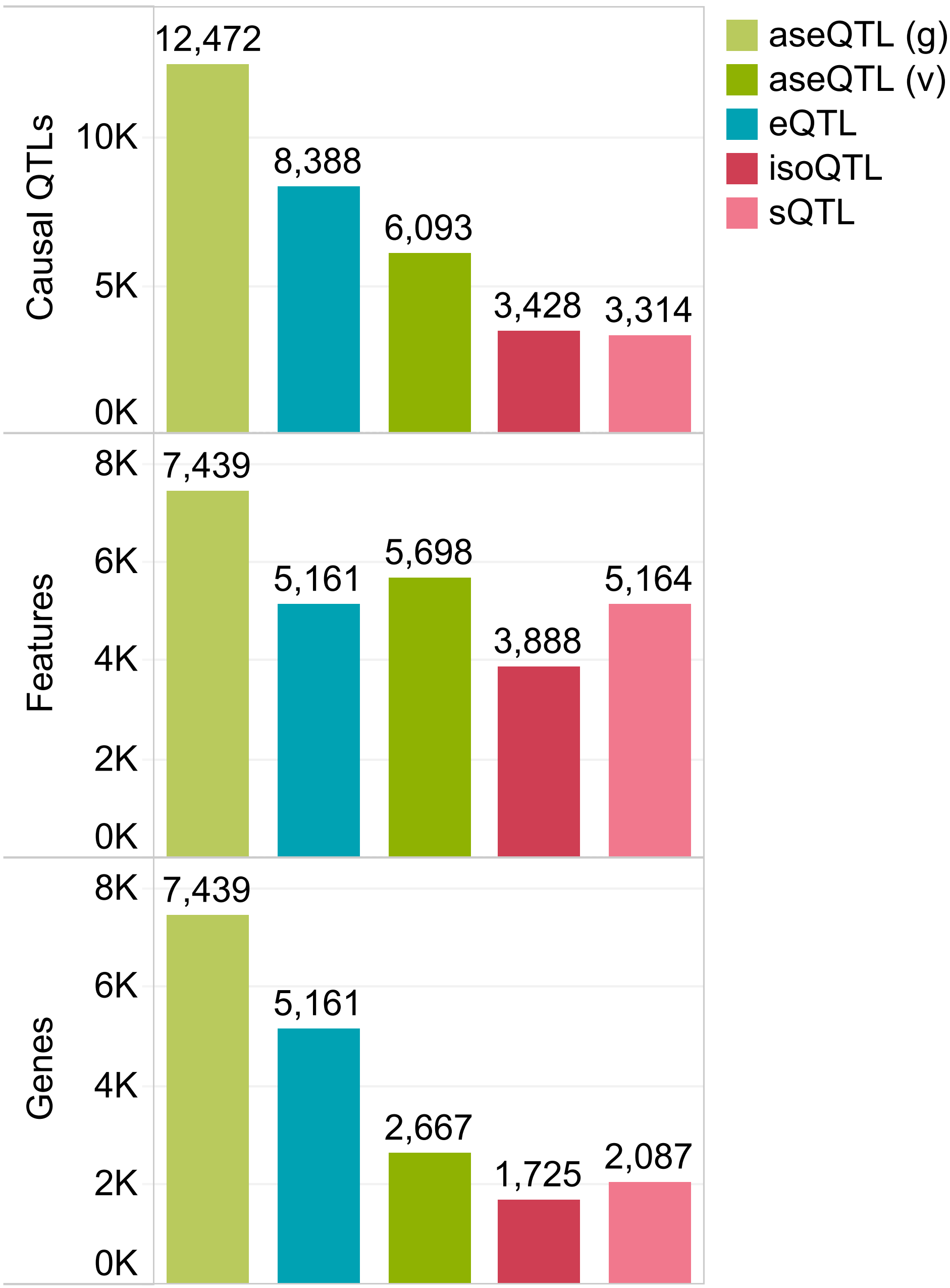

**Supplementary Fig. 14. Overlap of QBB/QGP Causal eQTLs with other Public Datasets.** UpSet plots showing the causal eQTLs, genes, and eQTL-gene pairs in the QGP/QBB dataset that are reported in various public cohorts of fine-mapped and univariate data. Intersections are colored with a gradient reflecting the corresponding counts displayed for each intersection.

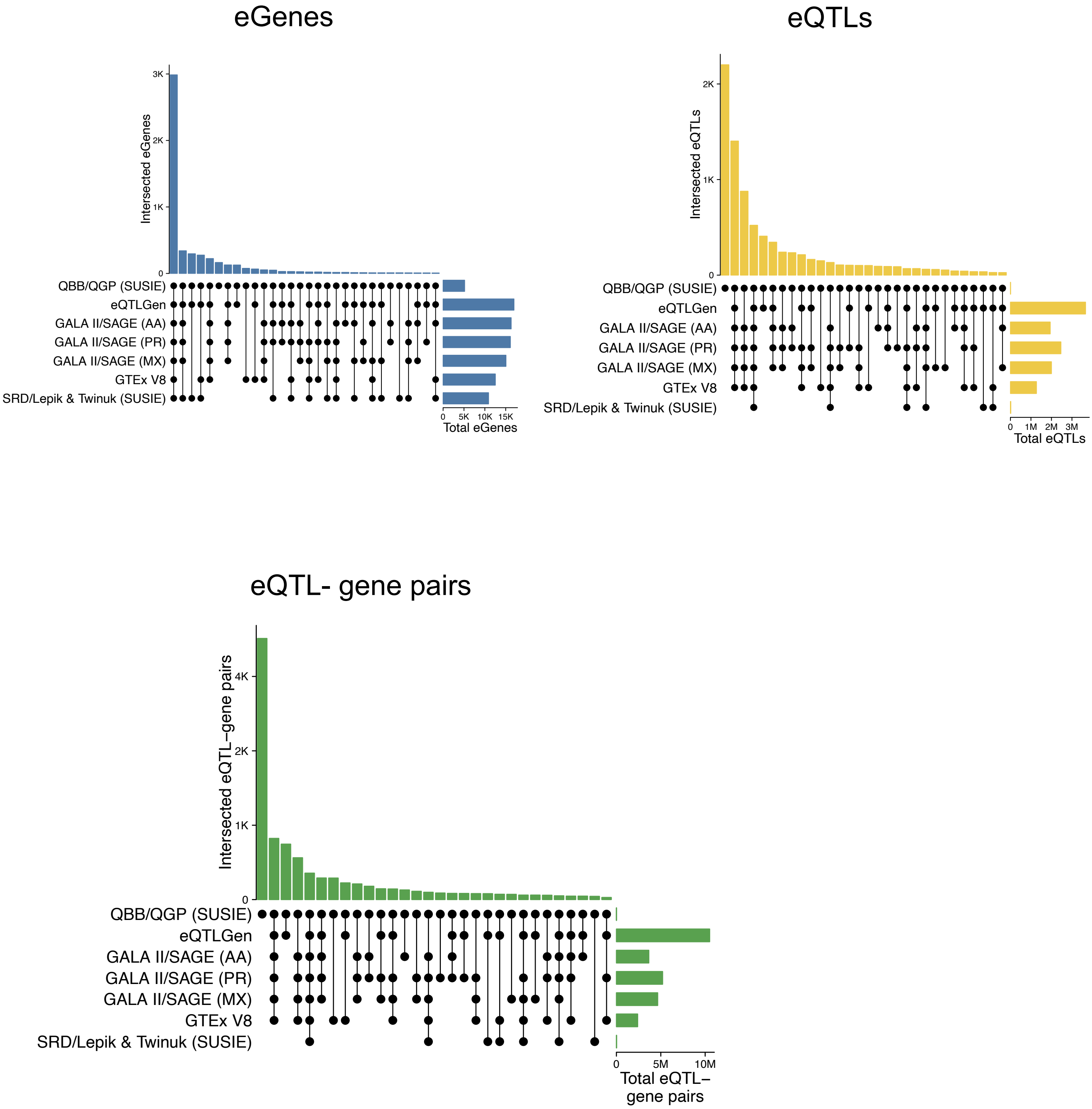

**Supplementary Fig. 15. Overlap of QBB/QGP Causal isoQTLs and sQTLs with other Public Datasets.** UpSet plot showing the causal QTLs, features, and QTL-feature pairs in the QGP/QBB dataset that are reported in various public cohorts of fine-mapped and univariate data, for isoform and splicing transcriptomic traits. Intersections are colored with a gradient reflecting the corresponding counts displayed for each intersection.

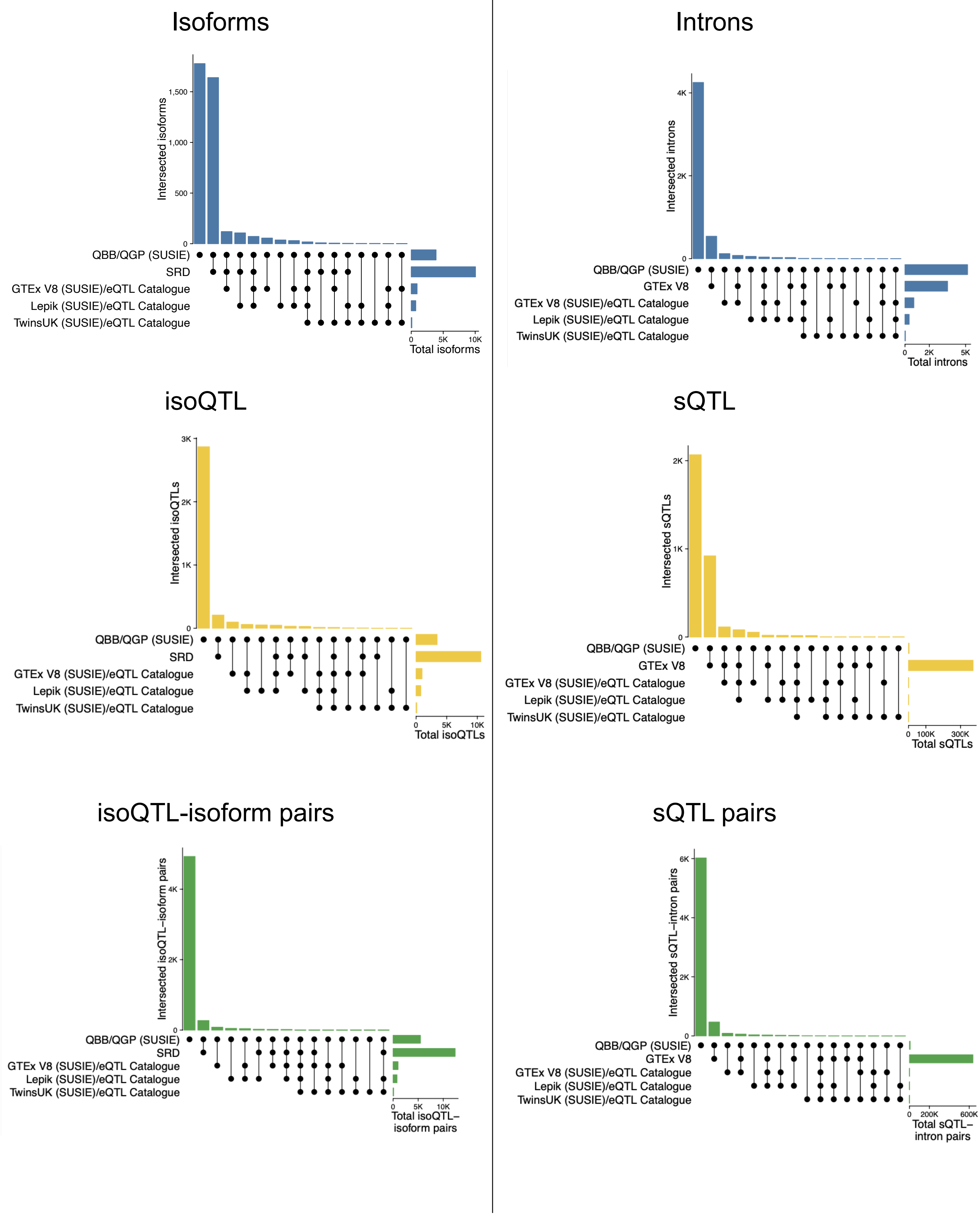

**Supplementary Fig. 16. Overlap of QBB/QGP Causal QTLs of All Types with other Public Datasets.** UpSet plot showing the causal QTLs, genes, and QTL-gene pairs of all QTL types in the QGP/QBB dataset that are reported in various public cohorts of fine-mapped and univariate data. Intersections are colored with a gradient reflecting the corresponding counts displayed for each intersection.

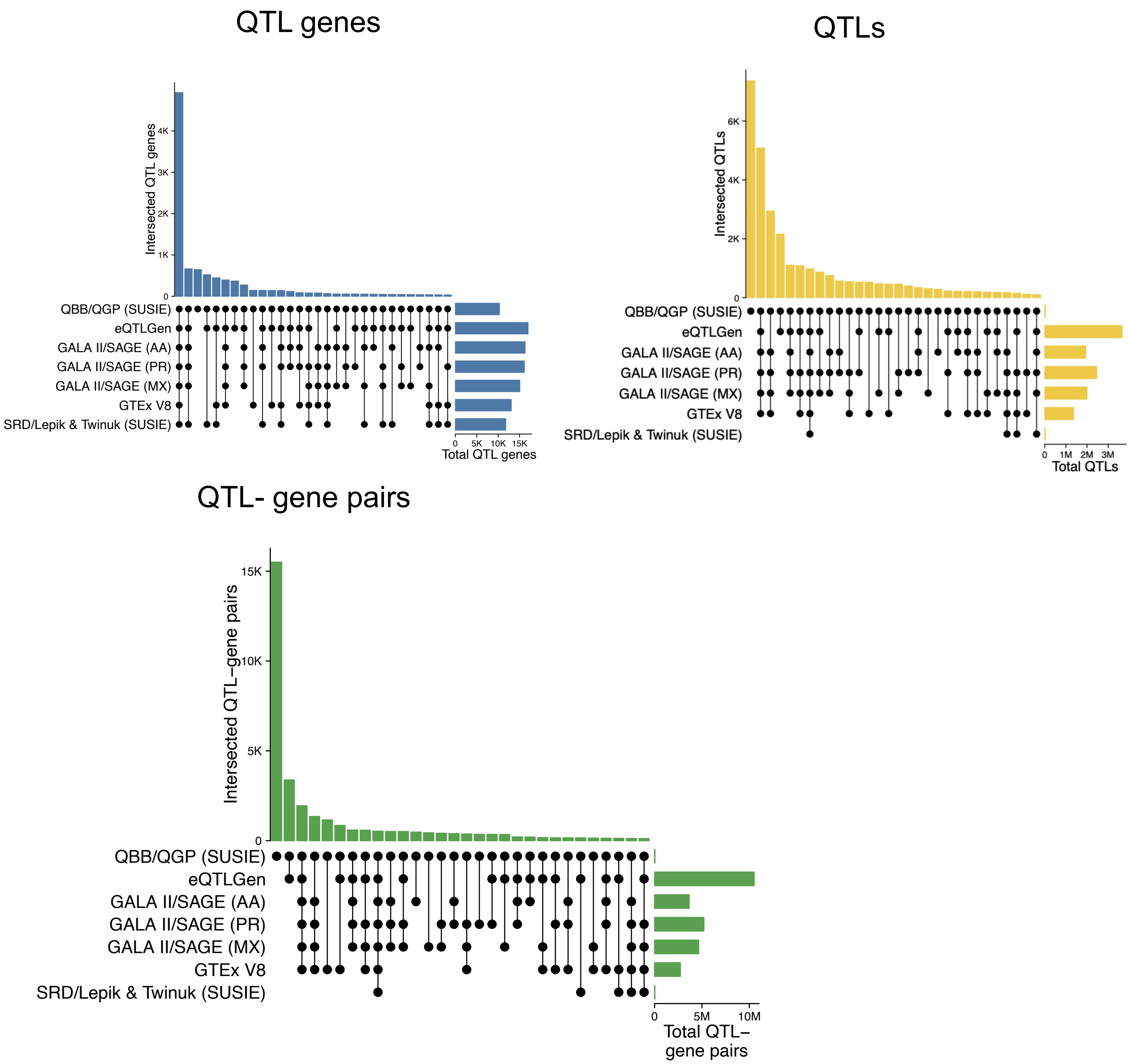

**Supplementary Fig. 17. Count of Genes Regulated by a Given Number of Causal QTLs. (a)** In QBB/QGP, **(b)** In other Public Datasets.

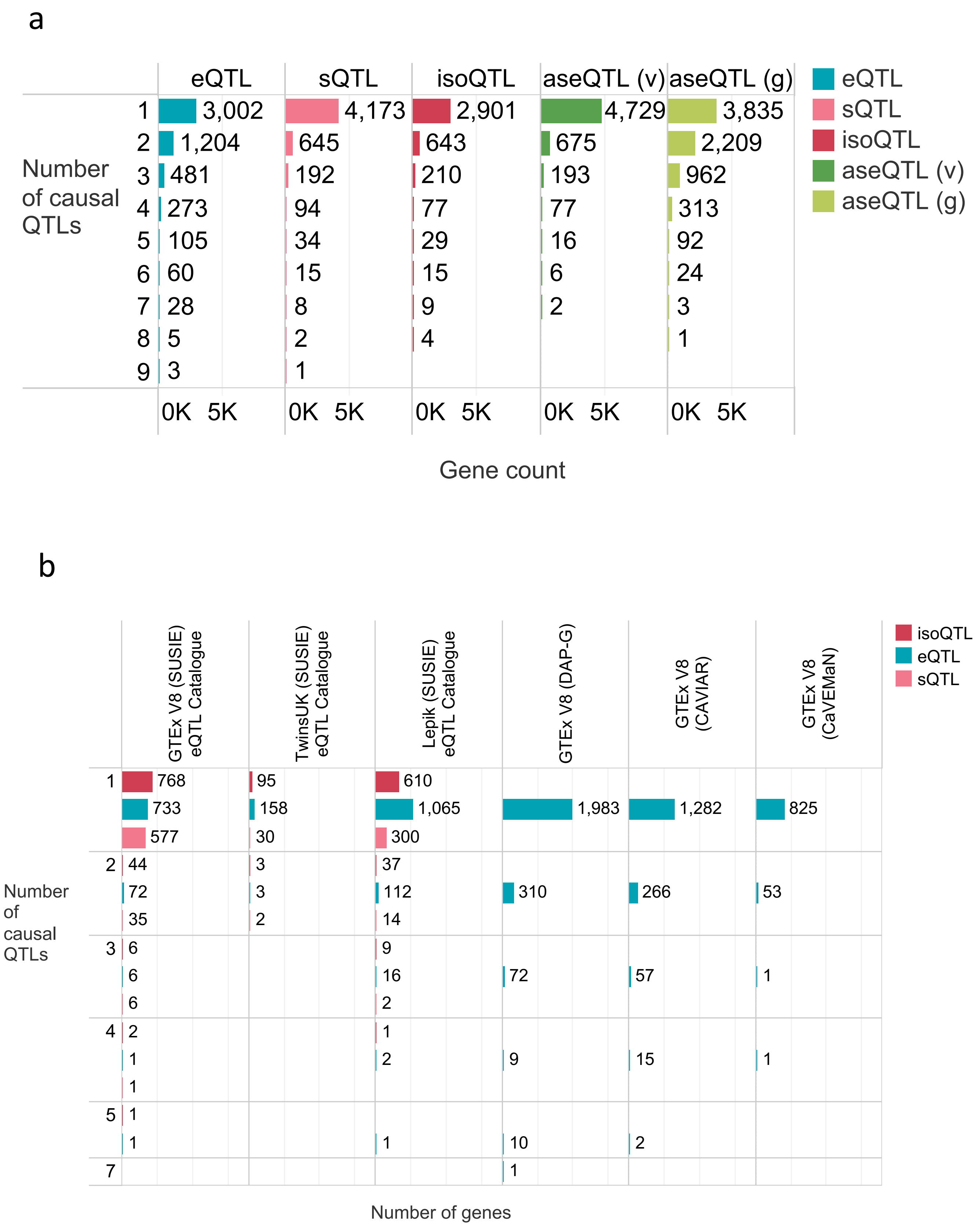

**Supplementary Fig. 18. Count of Causal QTLs in QBB/QGP regulating ACMG Genes**. Left bars are coloured by QTL type. Middle scatter plot shows causal QTLs' effect sizes (μ), with each dot representing a QTL-gene pair, colored by QTL type and shaped by VEP impact class. Right bars are coloured by VEP impact class.

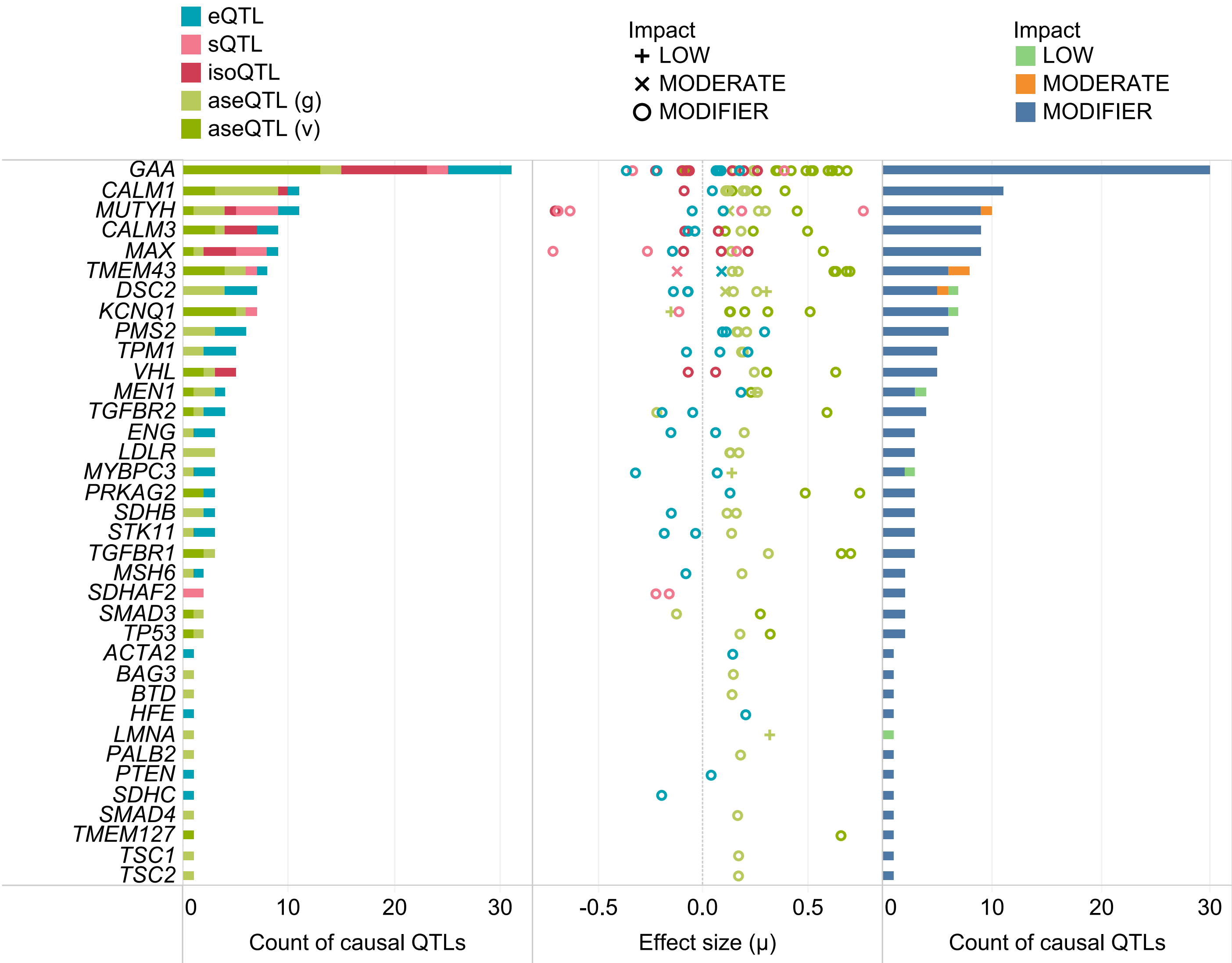

**Supplementary Fig. 19. Patterns of Divergent QBB/QGP Causal Variants in Global Ancestry Groups. (a)** Concordance of univariate effect sizes (slope β) between QBB/QGP and GTEx v8 for fine-mapped variant–gene pairs shared between the two studies (PIP > 0.95), both of which used the same SuSiE fine-mapping framework. **(b)** Volcano plots of AF ratios of causal QTLs in QBB/QGP relative to 1KG ancestry groups versus Bonferroni-adjusted p-values. Each dot represents a QTL and is colored based on $\log_2$ of the absolute AF ratio. **(c)** UpSet plot showing overlap of causal QTLs with significant AF differences between the QBB/QGP dataset and global ancestry groups from the 1KG project (Bonferroni p-value < 0.05). Intersecting areas are coloured with a gradient based on corresponding counts.

a

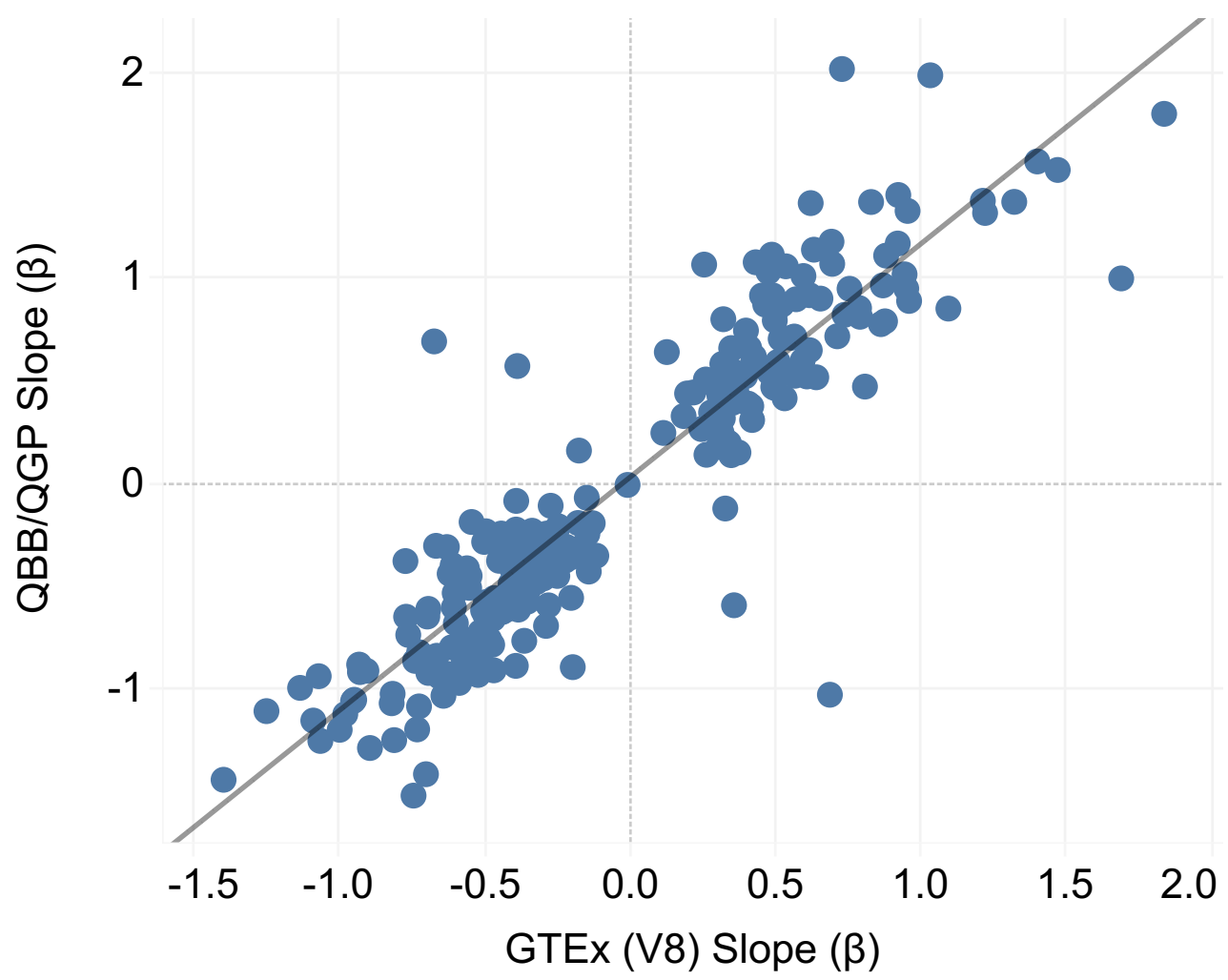


b

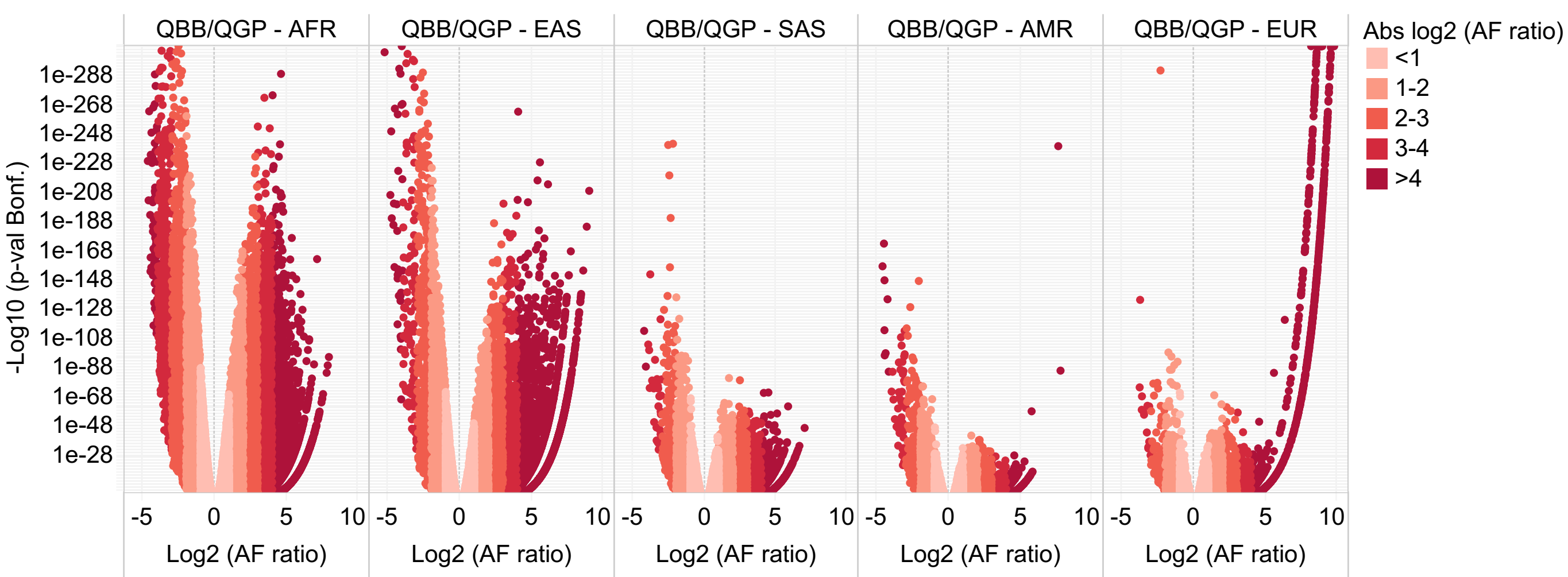

c

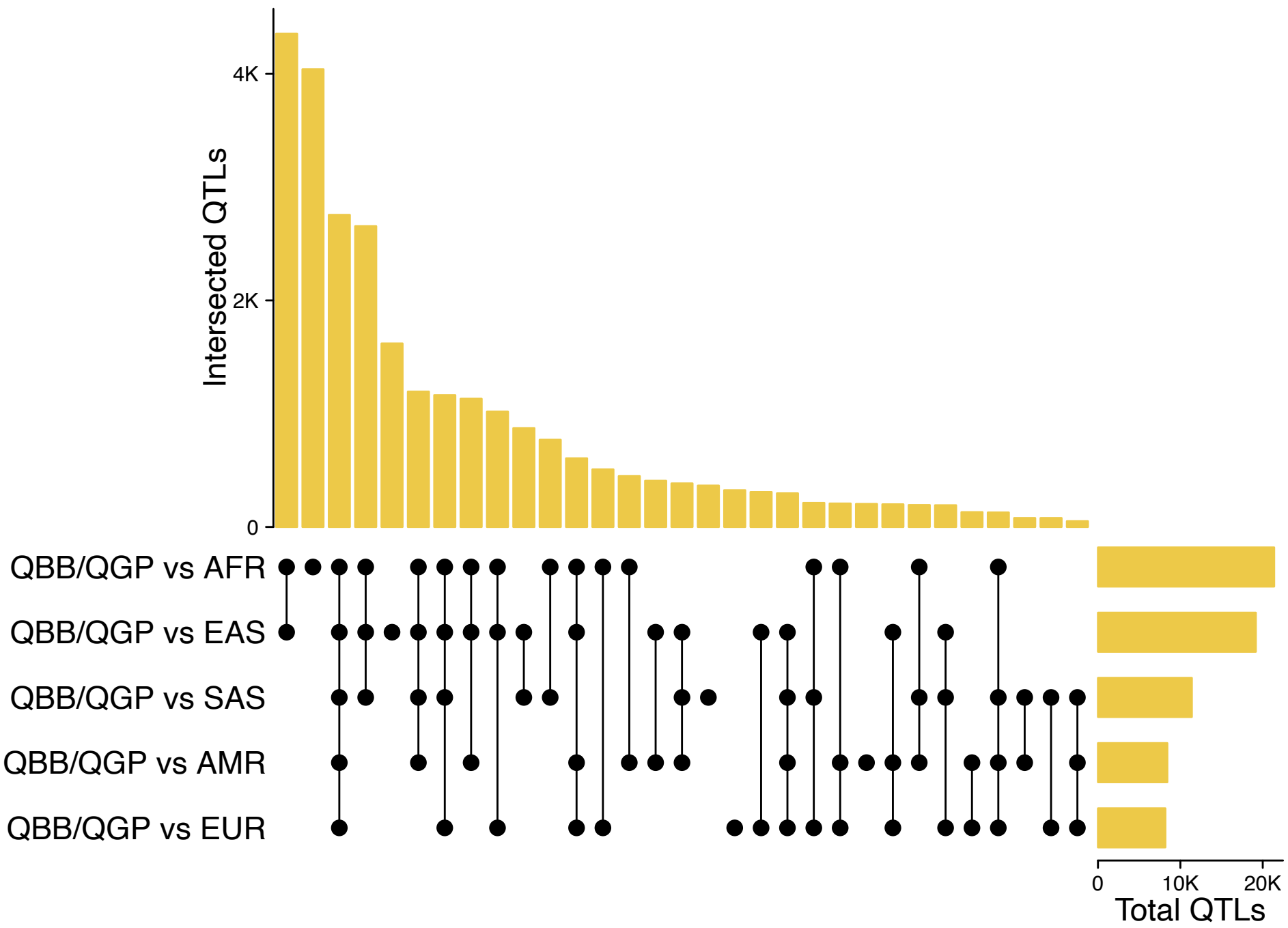

Intersected QTLs
4K
2K
0
QBB/QGP vs AFR
QBB/QGP vs EAS
QBB/QGP vs SAS
QBB/QGP vs AMR
QBB/QGP vs EUR
0
10K
20K
Total QTLs

**Supplementary Fig. 20. Highly Divergent Causal QTL SNVs Found in ClinVar.** ClinVar annotations for causal QTL SNVs in QBB/QGP showing significant AF differences against each of the 1KG ancestry groups (effect size > 4) and those only reported in QBB/QGP.

| Variant | RSID | QBB/QGP AF | Pub max AF | Consequence (VEP) | Gene name | Clinvar disease | QTL analysis | Mu | Clinvar class |
|---|---|---|---|---|---|---|---|---|---|
| 1:114449835:T:A | rs768197482 | 0.1589 | 0.2234 | intronic | *DCLRE1B* | not provided | eQTL | -0.12 | dark blue |
| 5:138665756:G:A | rs7305 | 0.6842 | 0.6820 | 3 prime UTR | *MATR3* | Amyotrophic lateral sclerosis type 21 | aseQTL (g) | 0.40 | dark blue |
| 11:65305450:G:A | rs199946104 | 0.0368 | 0.0101 | missense | *SCYL1* | not provided | sQTL | -0.38 | dark blue |
| | | | | | | | | 0.39 | dark blue |
| 14:94567097:T:C | rs149572076 | 0.0247 | 0.0010 | missense | *IFI27L1* | Inborn genetic diseases | sQTL | -0.24 | dark blue |
| 17:6493228:C:T | rs199909867 | 0.0412 | 0.0075 | missense | *KIAA0753* | not provided | aseQTL (g) | 0.13 | dark blue |
| 19:10277266:G:A | rs117294281 | 0.1123 | 0.0208 | splice region | *DNMT1* | Hereditary sensory neuropathy deafness dementia syndrome | sQTL | -0.63 | dark blue |
| 7:44102418:T:G | rs140230479 | 0.0286 | 0.0042 | missense | *DBNL* | Glycogen storage disease type X | eQTL | 0.13 | light blue |
| | | | | | | | isoQTL | -0.17 | light blue |
| | | | | | | | | -0.09 | light blue |
| | | | | | | | | 0.25 | light blue |
| | | | | | *PGAM2* | Glycogen storage disease type X | aseQTL (g) | 0.30 | light blue |
| 13:77566309:T:C | rs199727787 | 0.0389 | 0.0199 | missense | *CLN5* | Inborn genetic diseases\| Neuronal ceroid lipofuscinosis | aseQTL (g) | 0.19 | light blue |
| 17:902099:C:T | rs142757997 | 0.0329 | 0.0015 | missense | *TIMM22* | Inborn genetic diseases | aseQTL (g) | 0.15 | yellow |
| 5:132086583:A:G | rs768924657 | 0.0267 | 0.0002 | missense | *SEPT8* | Inborn genetic diseases | aseQTL (g) | 0.14 | yellow |
| 14:20945243:A:C | rs186060210 | 0.0361 | 0.0014 | 3 prime UTR | *PNP* | Purine-nucleoside phosphorylase deficiency | aseQTL (g) | 0.22 | yellow |
| 2:240897188:G:A | rs537694779 | 0.0260 | 0.0010 | 3 prime UTR | *NDUFA10* | Mitochondrial complex I deficiency, nuclear type 1\|Leigh syndrome | isoQTL | 0.15 | yellow |
| 22:21243115:C:A | rs868124207 | 0.0249 | Null | 3 prime UTR | *SNAP29* | CEDNIK syndrome | aseQTL (g) | 0.10 | yellow |

Clinvar class
- Benign (dark blue)
- Benign/Likely benign (dark blue)
- Likely benign (dark blue)
- Conflicting interpretation (light blue)
- Uncertain significance (yellow)

**Supplementary Fig. 21 Causal QTL Variants only reported in QBB/QGP and Their Divergence across Middle Eastern Ancestry Groups. (a)** Left: Number of fine-mapped QTLs with underlying variants that are only reported in QBB/QGP. Right: Count of impacted genes by biotype. **(b)** Density plot of the QTLs as a function of distance from transcription start sites (TSS). **(c)** Top: Volcano plot of AF ratio of only reported in QBB/QGP fine-mapped variants between pairwise QBB/QGP subpopulations versus Bonferroni-adjusted p-values. Middle: Corresponding pairwise comparisons of AFs between QBB/QGP ancestries. Each dot represents a variant. Coloured dots indicate significantly different AFs (Bonferroni-adjusted p-value < 0.05), while grey ones that are not significant. Blue indicates QTLs unique to a particular ancestry, while red indicates QTLs shared between both ancestry, with a gradient reflecting the magnitude of the AF difference. Bottom: Bar plot showing the number of fine-mapped QTLs per comparison.

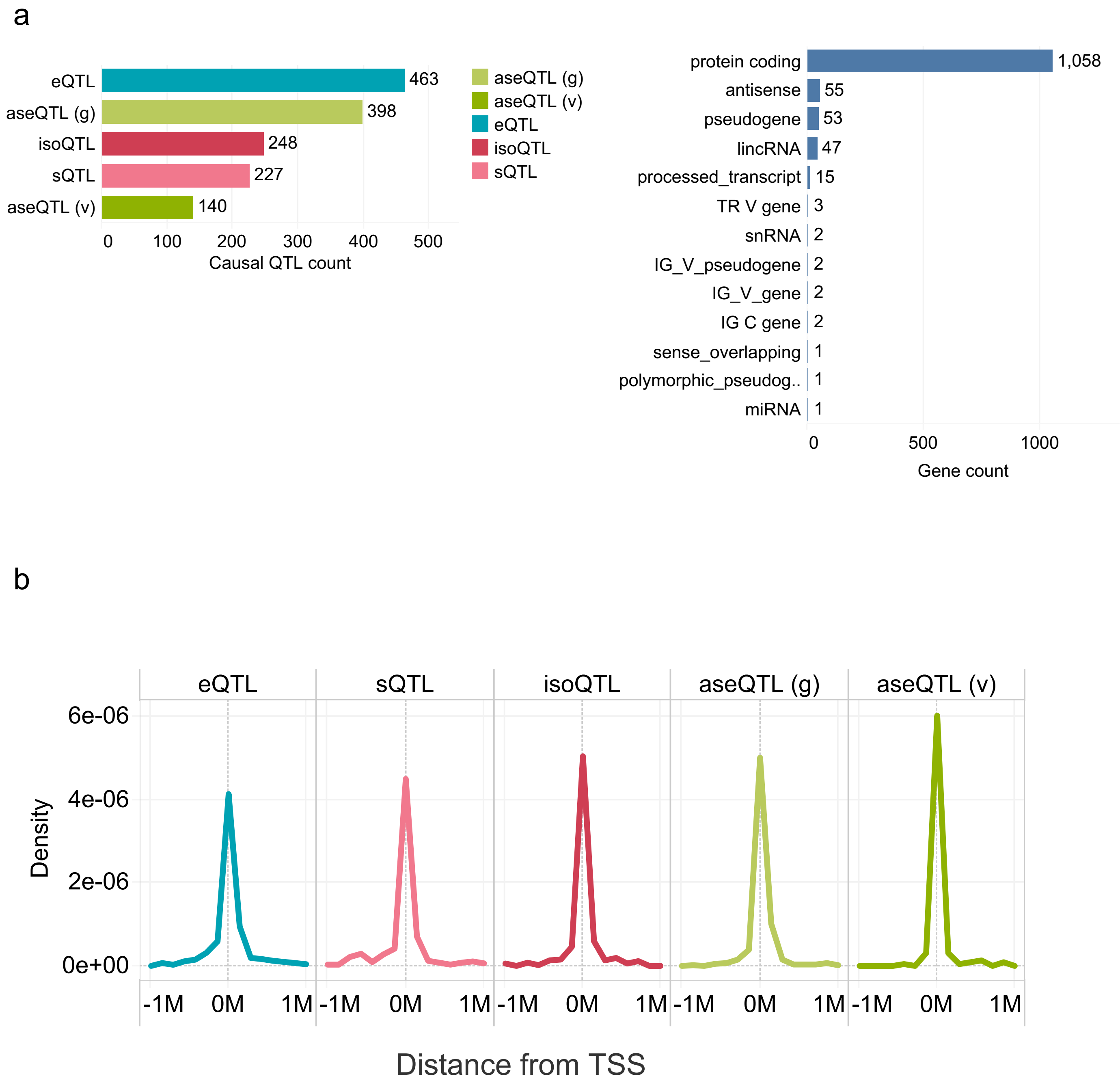

c

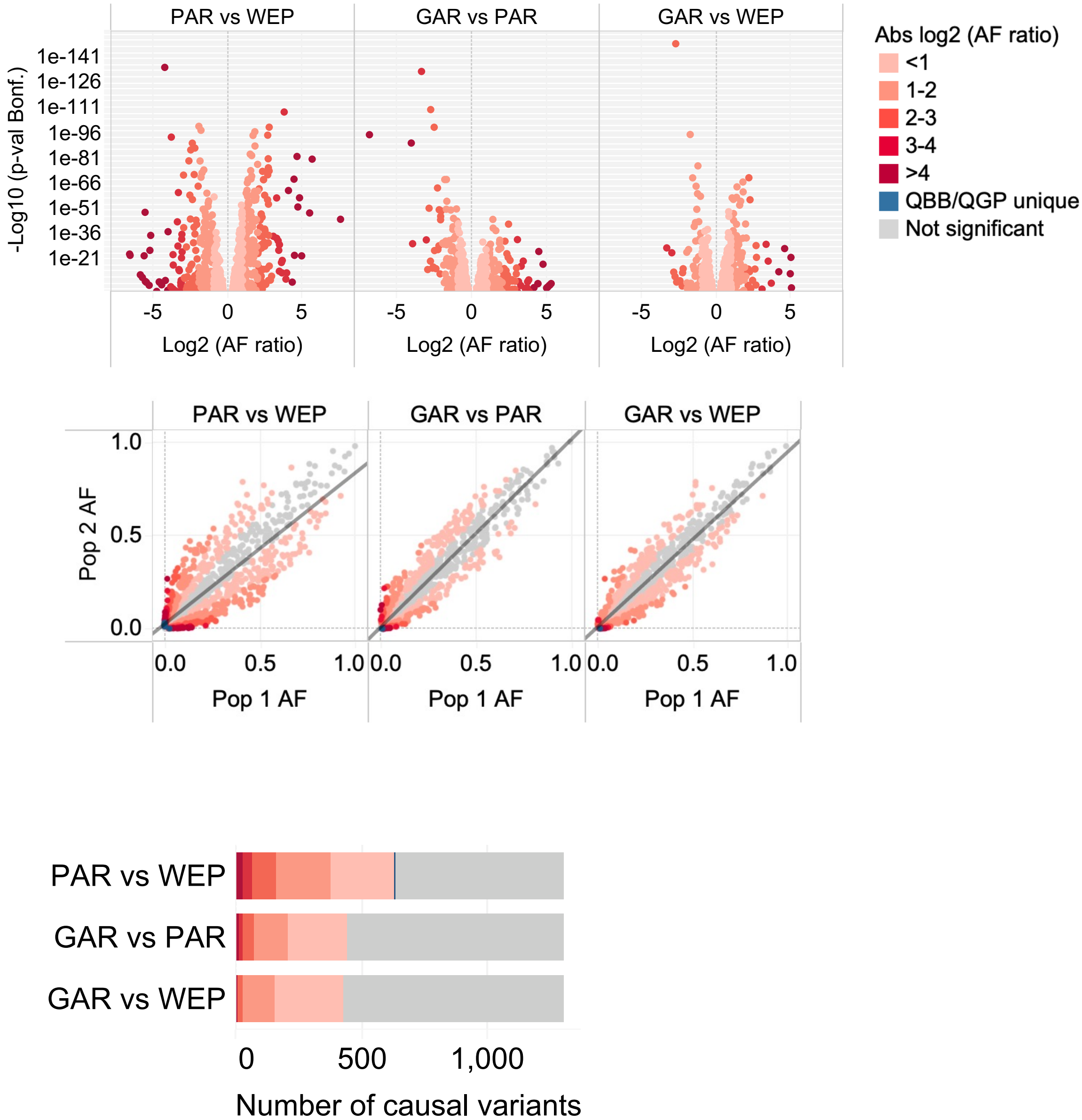

PAR vs WEP
GAR vs PAR
GAR vs WEP
-Log10 (p-val Bonf.)
1e-141
1e-126
1e-111
1e-96
1e-81
1e-66
1e-51
1e-36
1e-21
-5
0
5
Log2 (AF ratio)
Abs log2 (AF ratio)
<1
1-2
2-3
3-4
>4
QBB/QGP unique
Not significant
Pop 2 AF
1.0
0.5
0.0
Pop 1 AF
Number of causal variants
0
500
1,000

**Supplementary Fig. 22. Univariate SV QTLs in the QBB/QGP cohort. (a)** Numbers of significant SV QTLs, associated genes, features, and QTL-feature pairs by SV type and QTL type. **(b)** Box plot of SV QTL length (bp) by SV type and QTL type. **(c)** Count of significant SV QTLs per SV type and their genomic locations, annotated by SVAnnotate.

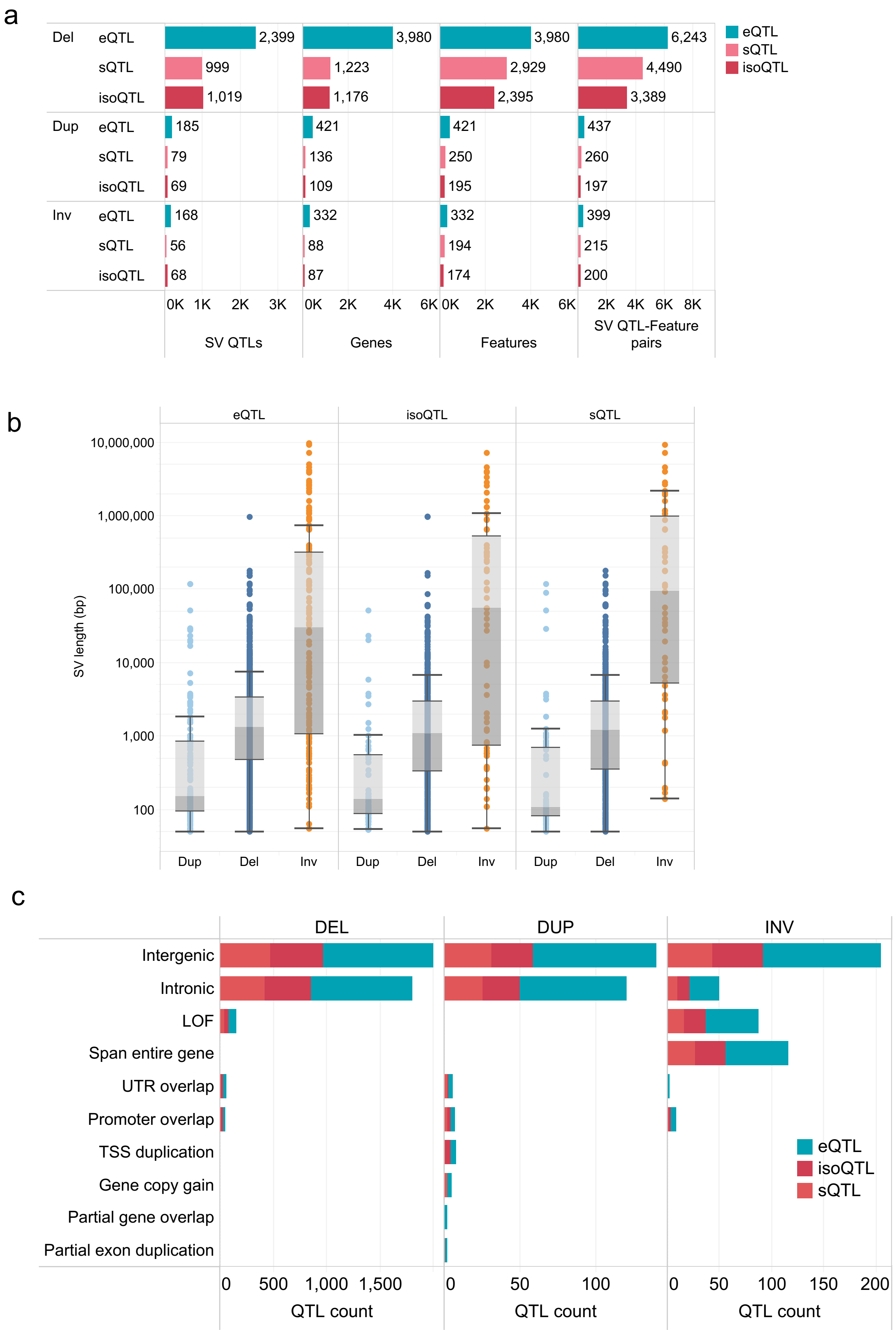

**Supplementary Fig. 23: Patterns of Causal SV QTLs. (a)** (Top) Effect size (μ) versus log Bayes factors (LBF) and (bottom) effect size (μ) versus Posterior Inclusion Probability (PIP) for SV causal QTLs identified in this study. Each dot is a QTL and coloured by Z score. **(b)** Density plot of causal SV QTLs as a function of distance from the transcription start site (TSS). **(c)** Distribution of SV QTL lengths by SV type. **(d)** Effect size (μ) versus QBB/QGP allele frequency (AF) for the SV causal QTLs. Each dot represents a QTL. **(e)** Box plots of AF (top) and absolute effect size (μ) (bottom) for causal SV QTLs, stratified by SV and QTL type. **(f)** AF of causal QTLs in QBB/QGP versus maximum AF in gnomAD. Each dot represents a QTL, coloured by QTL type and shaped by SV type. The Pearson correlation trend line is shown in grey (R = 0.7, p < 0.0001).

a

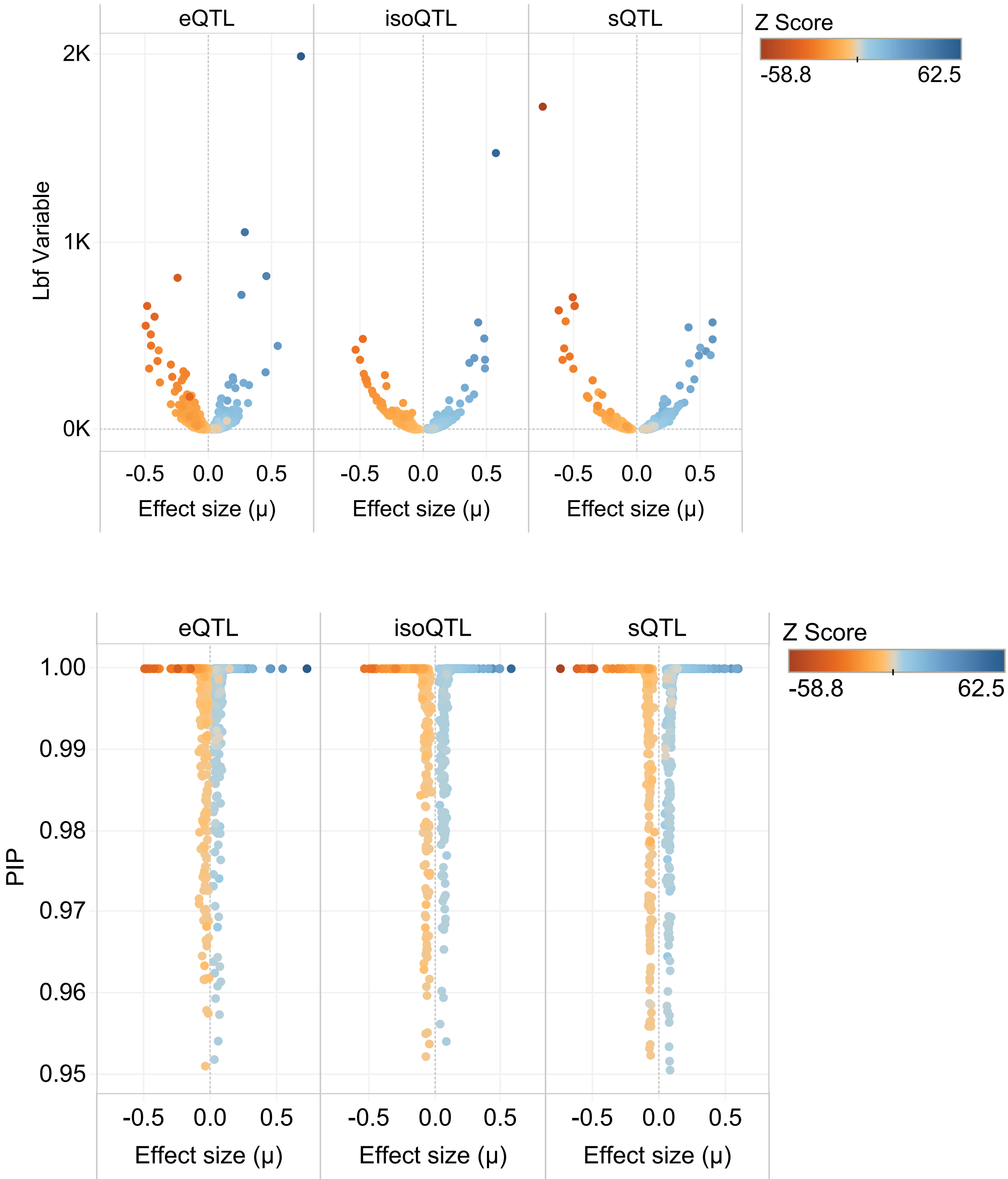

b

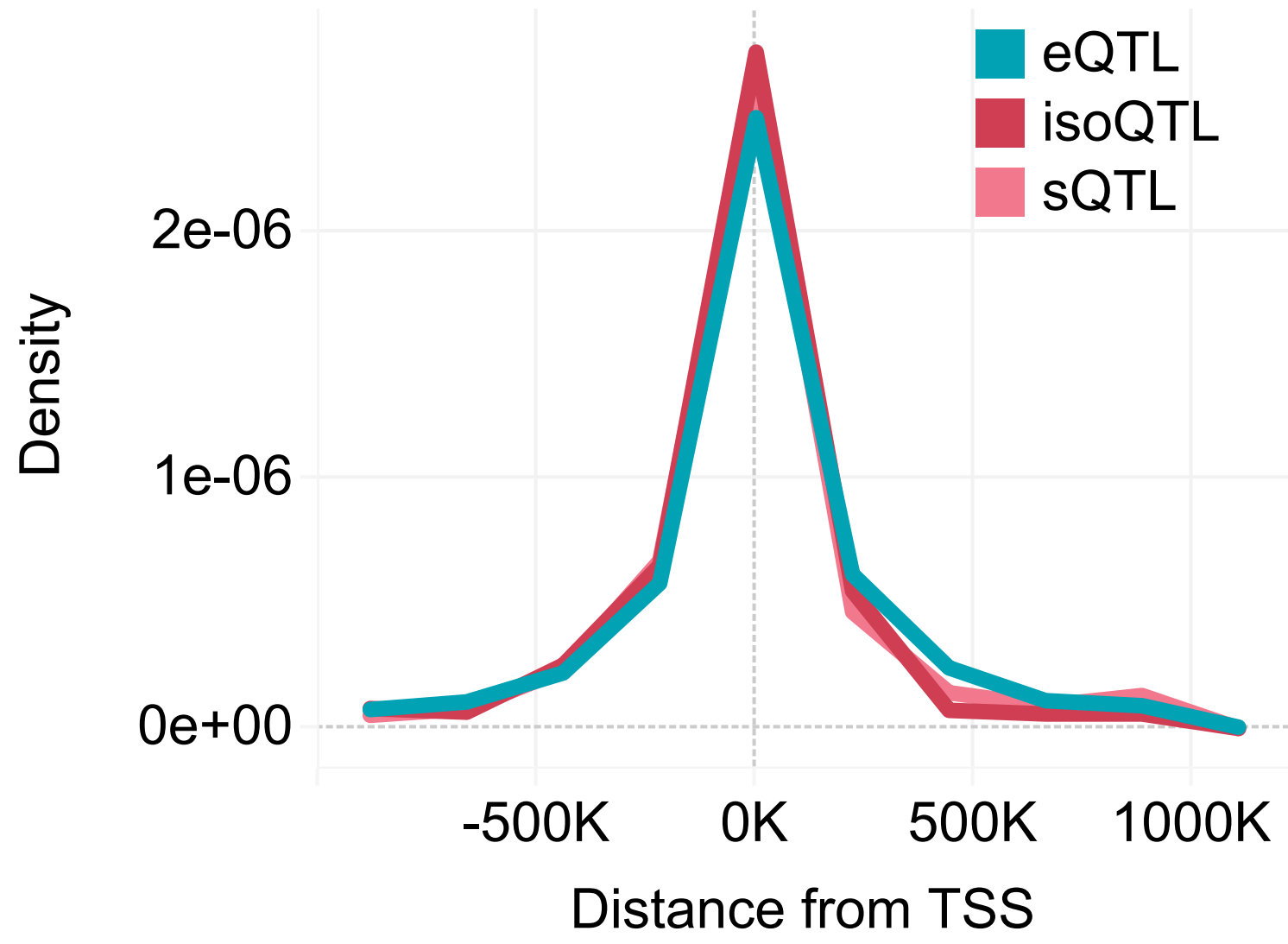
eQTL
isoQTL
sQTL
Density
2e-06
1e-06
0e+00
-500K
0K
500K
1000K
Distance from TSS

c

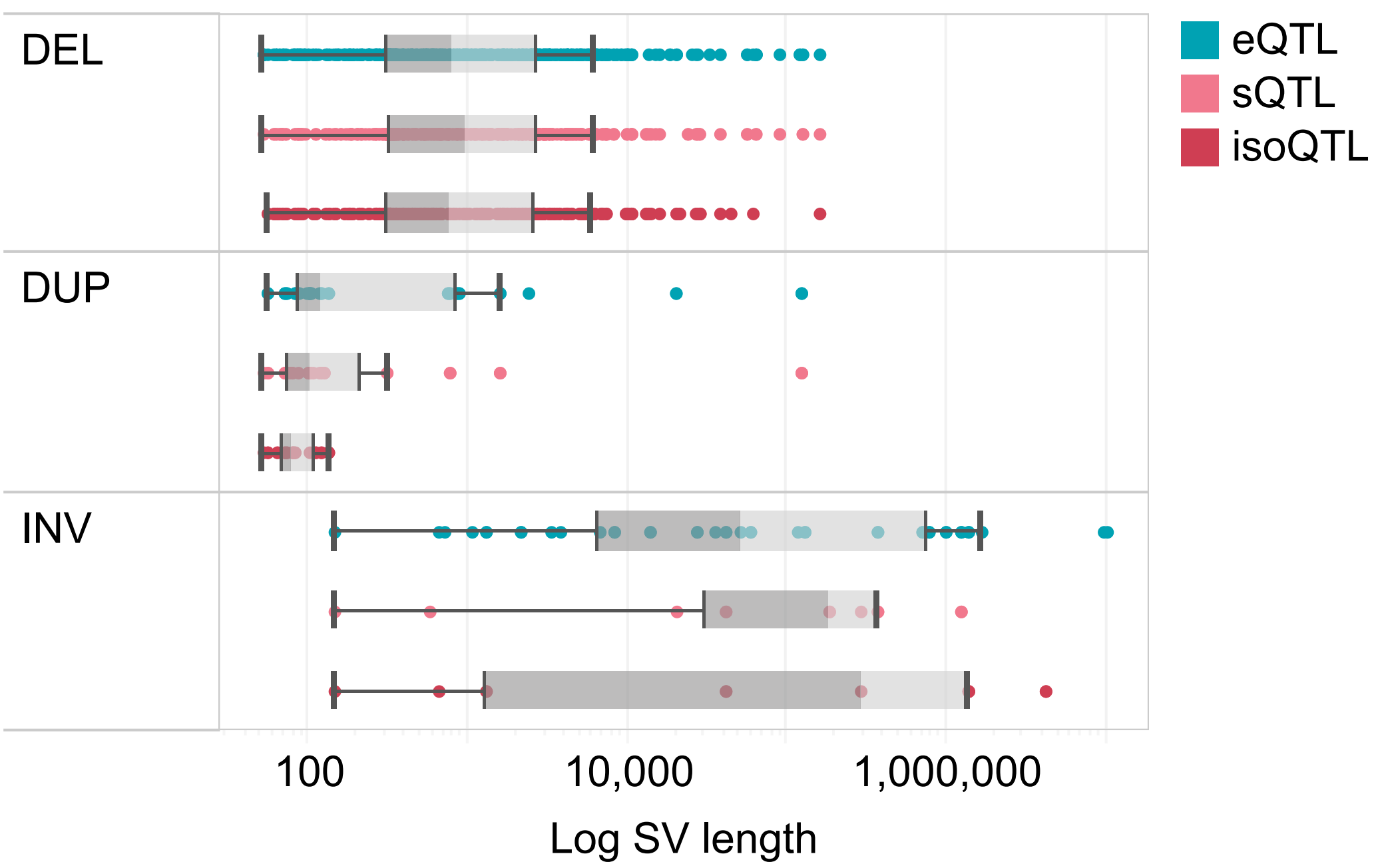
DEL
DUP
INV
eQTL
sQTL
isoQTL
100
10,000
1,000,000
Log SV length

d

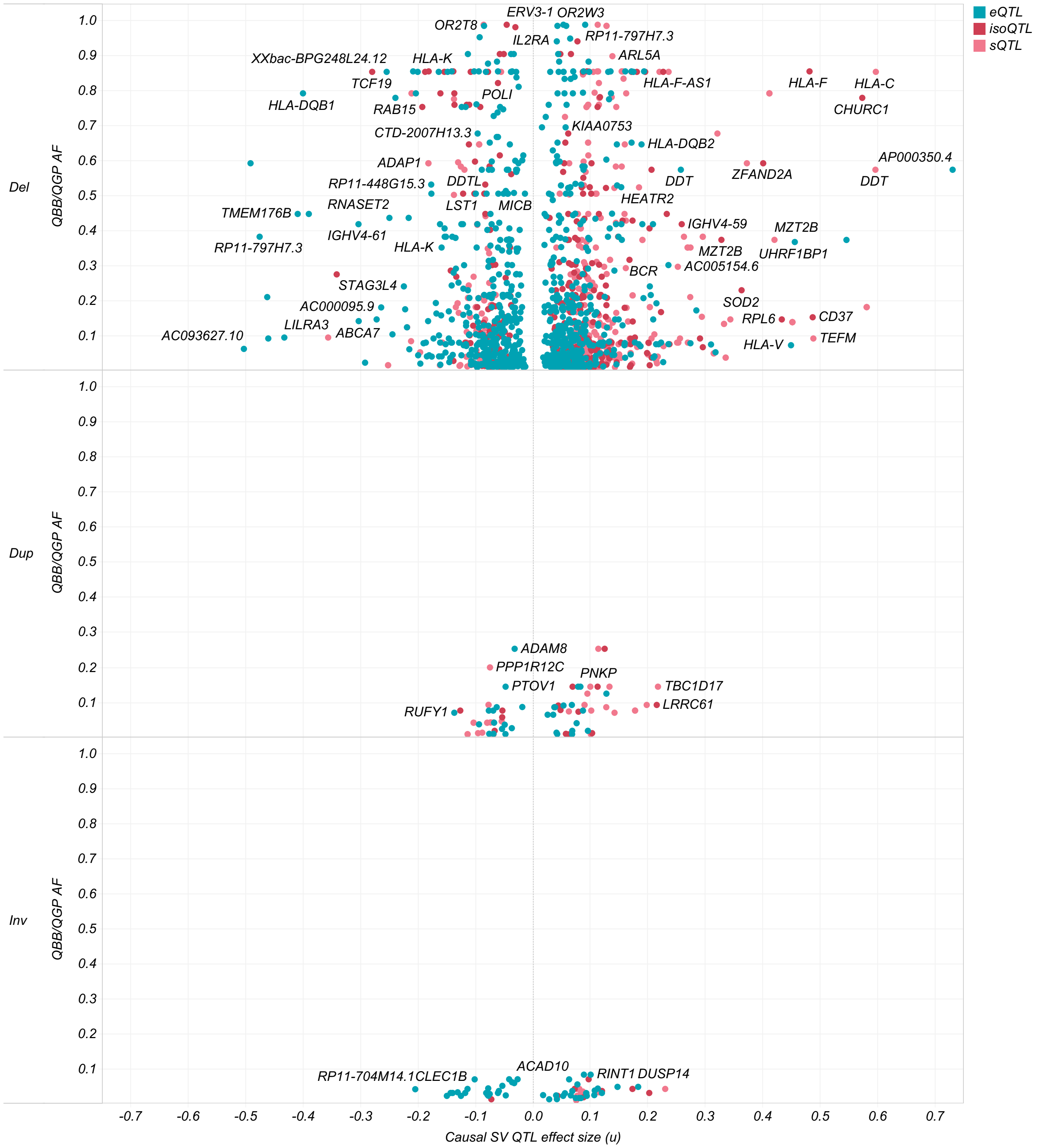
eQTL
isoQTL
sQTL
Del
Dup
Inv
QBB/QGP AF
Causal SV QTL effect size (u)
ERV3-1 OR2W3
OR2T8
IL2RA
RP11-797H7.3
XXbac-BPG248L24.12
HLA-K
ARL5A
TCF19
HLA-F-AS1
HLA-F
HLA-C
POLI
HLA-DQB1
RAB15
CHURC1
KIAA0753
CTD-2007H13.3
HLA-DQB2
ADAP1
AP000350.4
ZFAND2A
RP11-448G15.3
DDTL
DDT
DDT
HEATR2
RNASET2
LST1
MICB
TMEM176B
IGHV4-59
MZT2B
IGHV4-61
HLA-K
RP11-797H7.3
MZT2B
UHRF1BP1
AC005154.6
BCR
STAG3L4
SOD2
AC000095.9
RPL6
CD37
LILRA3
ABCA7
AC093627.10
HLA-V
TEFM
ADAM8
PPP1R12C
PNKP
PTOV1
TBC1D17
LRRC61
RUFY1
ACAD10
RP11-704M14.1CLEC1B
RINT1 DUSP14

e

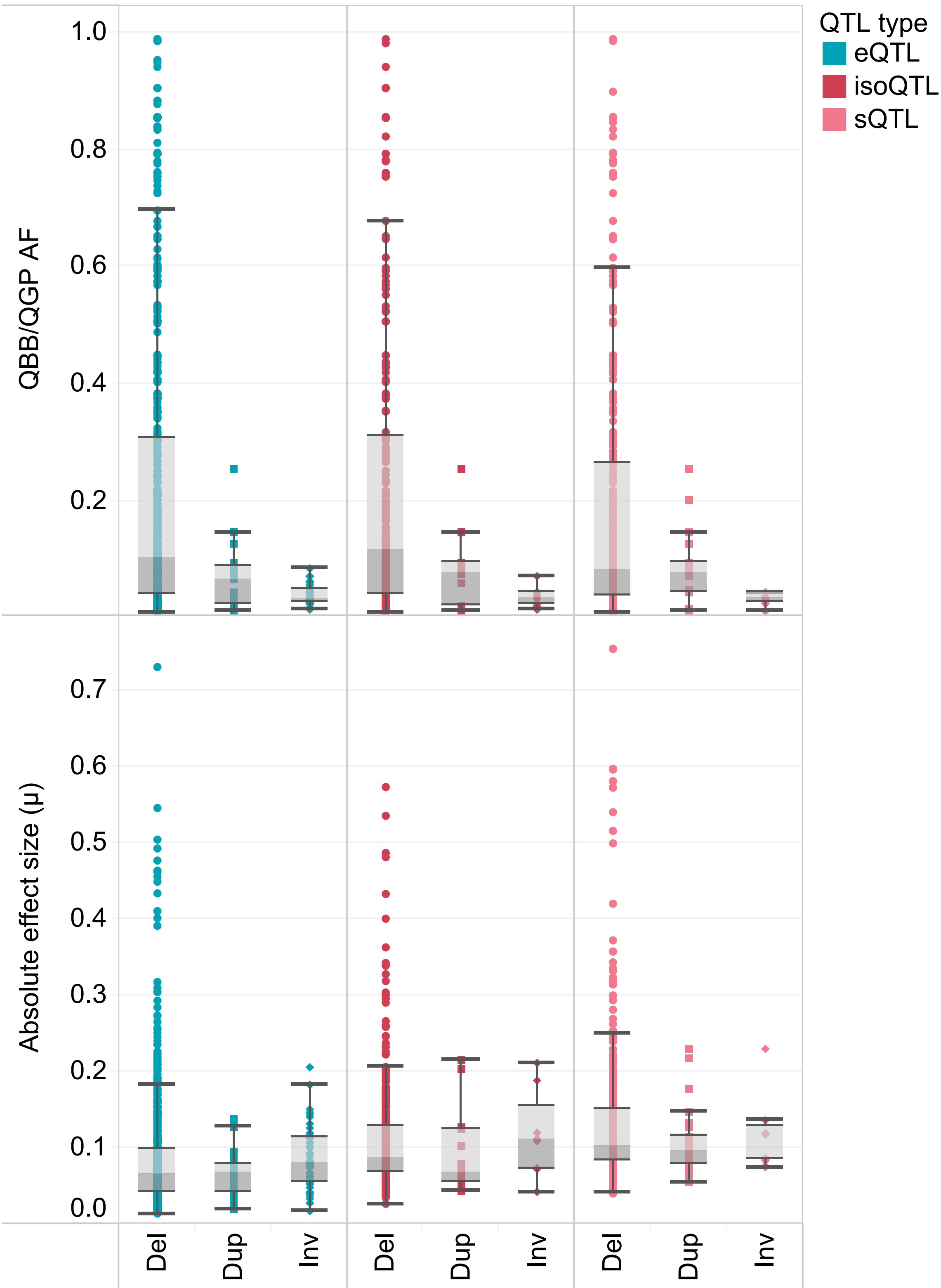
QTL type
eQTL
isoQTL
sQTL
QBB/QGP AF
1.0
0.8
0.6
0.4
0.2
Absolute effect size (μ)
0.7
0.6
0.5
0.4
0.3
0.2
0.1
0.0
Del
Dup
Inv
Del
Dup
Inv
Del
Dup
Inv

f

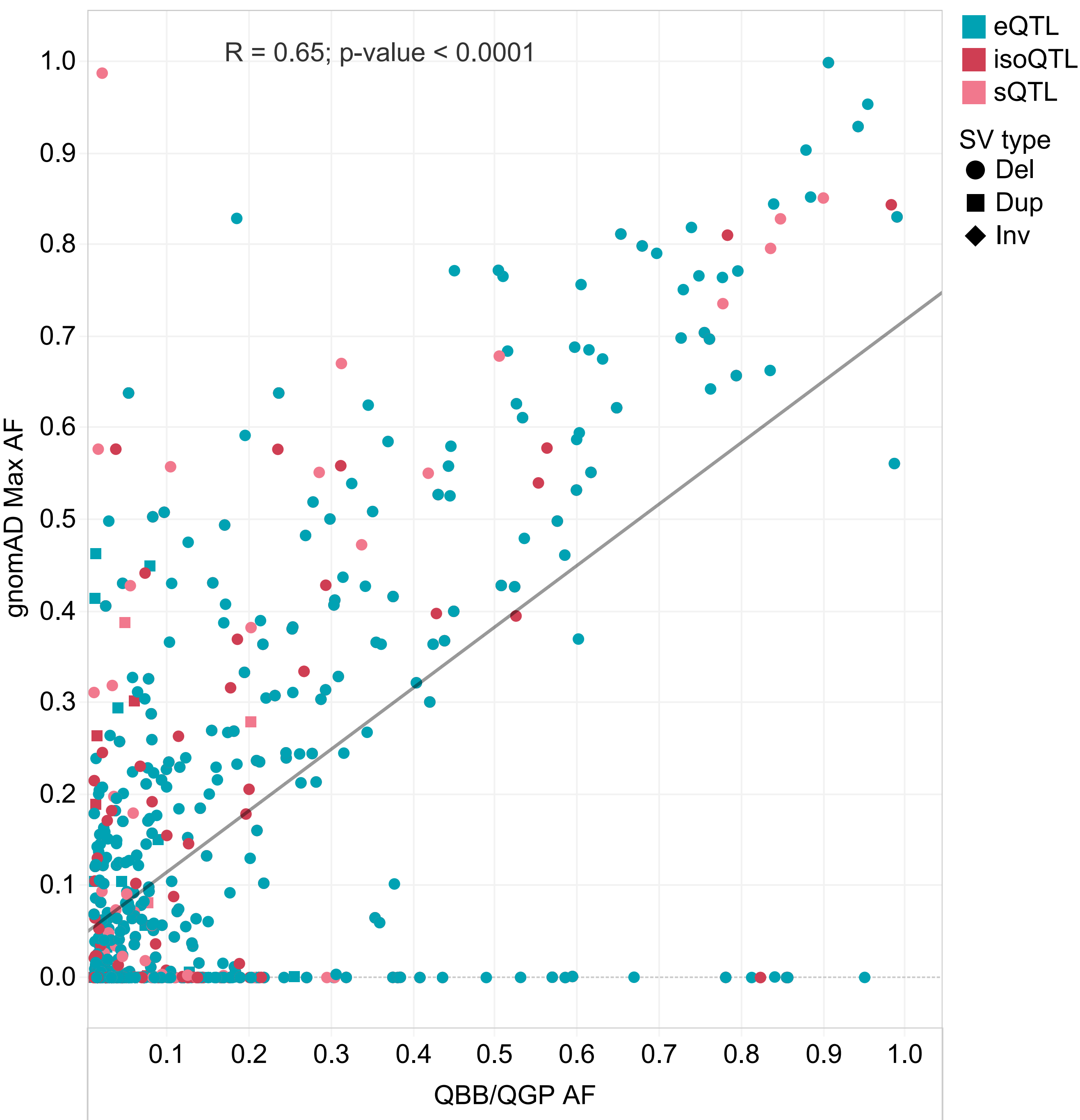
R = 0.65; p-value < 0.0001
gnomAD Max AF
QBB/QGP AF
0.0
0.1
0.2
0.3
0.4
0.5
0.6
0.7
0.8
0.9
1.0
eQTL
isoQTL
sQTL
SV type
Del
Dup
Inv

**Supplementary Fig. 24. Pipeline of Colocalization of Causal QTLs with GWAS Loci.** Flow chart indicates the various steps and related parameters used in the coloc/SuSiE workflow implemented in this study.

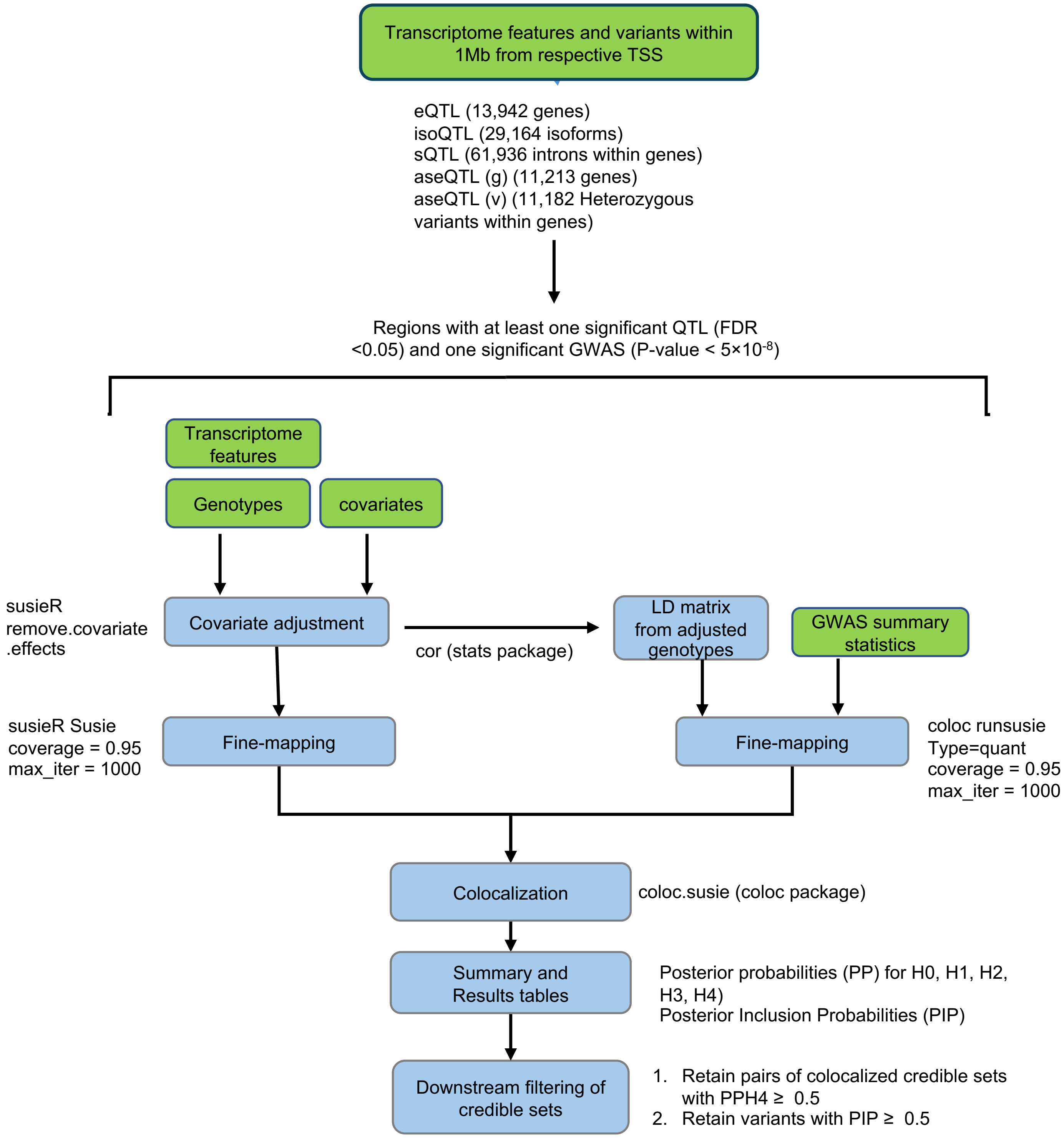

**Supplementary Fig. 25. Colocalized Causal Variants and Their Effect Sizes for Various Transcriptome Traits and GWAS phenotypes.** Scatter plot of colocalized variants depicting the effect sizes for transcriptome traits and GWAS phenotypes. Each dot is a QTL, labelled with the gene, biobank phenotype and coloured by QTL type.

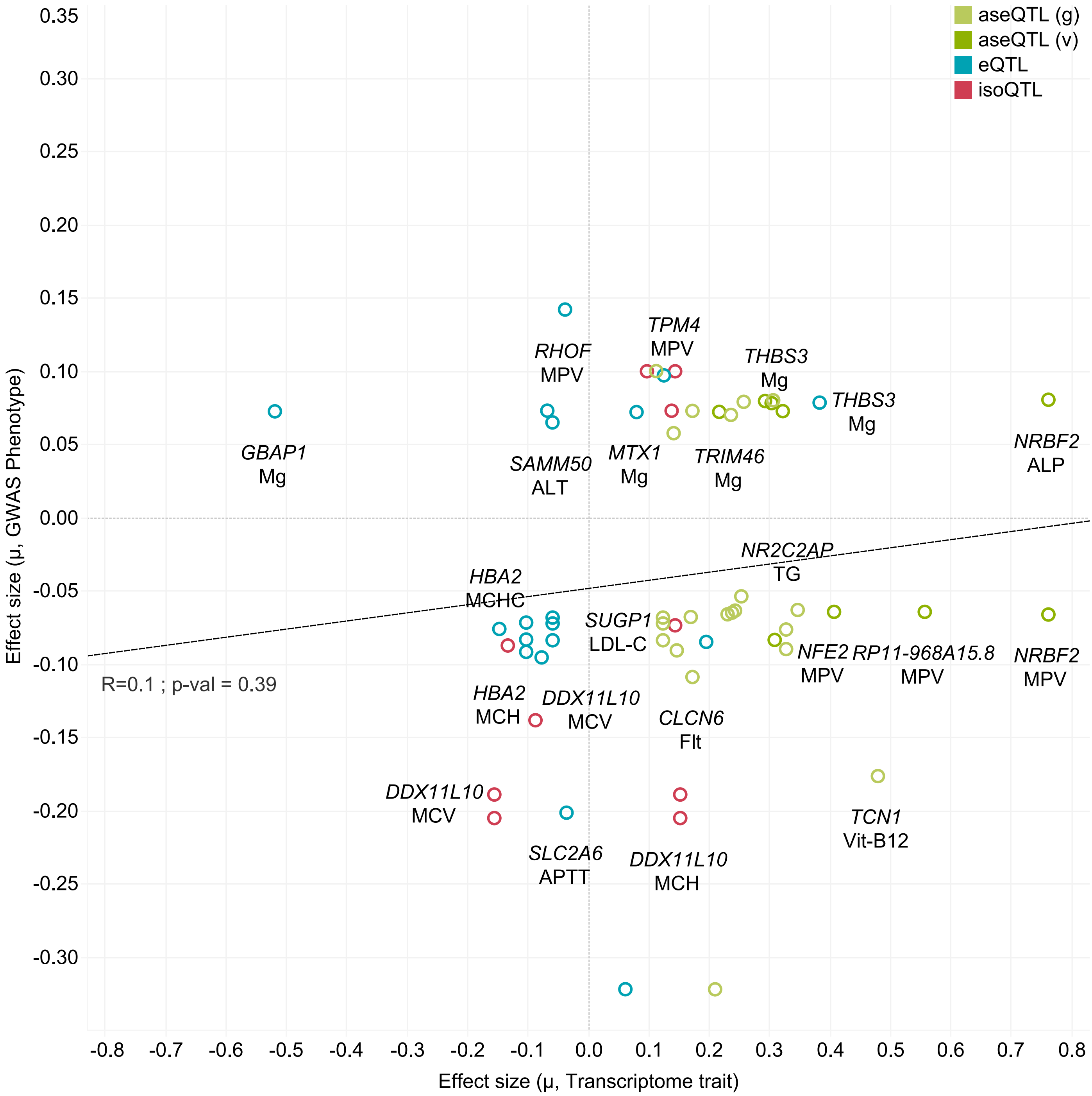

Supplement: Supplementary file 1 [file 41588_2026_2785_MOESM1_ESM.pdf]
