# Supplementary material for: A catalog of transcriptome genetic regulation in a consanguineous Middle Eastern biobank
Source: Nat Genet. 2026 Nov 2;58(11):2832–44. doi: 10.1038/s41588-026-02785-7 (PMC13754583; doi:10.1038/s41588-026-02785-7)
Supplement: Supplementary file 2 [file 41588_2026_2785_MOESM2_ESM.pdf]

nature portfolio

Corresponding author(s): Younes Mokrab

Last updated by author(s): Jul 23, 2026

Reporting Summary

Nature Portfolio wishes to improve the reproducibility of the work that we publish. This form provides structure for consistency and transparency in reporting. For further information on Nature Portfolio policies, see our Editorial Policies and the Editorial Policy Checklist.

Statistics

For all statistical analyses, confirm that the following items are present in the figure legend, table legend, main text, or Methods section.

n/a	Confirmed	
☐	☒	The exact sample size (*n*) for each experimental group/condition, given as a discrete number and unit of measurement
☐	☒	A statement on whether measurements were taken from distinct samples or whether the same sample was measured repeatedly
☐	☒	The statistical test(s) used AND whether they are one- or two-sided<br>*Only common tests should be described solely by name; describe more complex techniques in the Methods section.*
☐	☒	A description of all covariates tested
☐	☒	A description of any assumptions or corrections, such as tests of normality and adjustment for multiple comparisons
☐	☒	A full description of the statistical parameters including central tendency (e.g. means) or other basic estimates (e.g. regression coefficient) AND variation (e.g. standard deviation) or associated estimates of uncertainty (e.g. confidence intervals)
☐	☒	For null hypothesis testing, the test statistic (e.g. *F*, *t*, *r*) with confidence intervals, effect sizes, degrees of freedom and *P* value noted<br>*Give P values as exact values whenever suitable.*
☒	☐	For Bayesian analysis, information on the choice of priors and Markov chain Monte Carlo settings
☒	☐	For hierarchical and complex designs, identification of the appropriate level for tests and full reporting of outcomes
☐	☒	Estimates of effect sizes (e.g. Cohen's *d*, Pearson's *r*), indicating how they were calculated

Our web collection on statistics for biologists contains articles on many of the points above.

Software and code

Policy information about availability of computer code

Data collection	HiSeq X Ten (Illumina, San Diego, CA, USA), Illumina HiSeq 4000 instrument
Data analysis	AnnotSV v2.2, Bcftools v1.22/v1.3/v1.10, BWA-MEM v0.7.7, Breakdancer v1.4.5, Breakseq2 v2.2, coloc v5.2.3, CNVnator v1.0.0, ComplexHeatmap V2.22.0, Delly v0.7.8, DESeq2 v1.24.0, ERDS v1.0.2, FastQTL v2.184, FastQC v0.11.2, GATK v3.4, Genome STRiP v2.00.1711, ggVennDiagram v1.2.3/v1.5.2/v1.7.3, Ingenuity Pathway Analysis (IPA) v01-22-01, LeafCutter v0.2.9, Manta v1.6.0, Matrix eQTL v2.3, multiQC v1.21, multcomp v1.4.19, OUTRIDER v1.20.0, phASER v1.1.1, Picard v2.6.0, PLINK v2.0.0-a.6LM, qqman v0.1.9, RNOmni v1.0.1 , SeqyClean v1.10.07, SortMeRNA v2.1, SpeedSeq v0.1.2, STAR v2.7.3a/v2.5.0a, Subread v1.5.1, susieR v0.12.3, SURVIVOR v1.0.7, SVAnnotate (GATK) v4.4.0.0, SvABA v0.2.3, Trimmomatic v0.35, Variant Effect Predictor v99.0, VennDiagram v1.7.3, verifyBAMID v1.1.3, WHAM v1.7.0.

For manuscripts utilizing custom algorithms or software that are central to the research but not yet described in published literature, software must be made available to editors and reviewers. We strongly encourage code deposition in a community repository (e.g. GitHub). See the Nature Portfolio guidelines for submitting code & software for further information.

Data

Policy information about availability of data

All manuscripts must include a data availability statement. This statement should provide the following information, where applicable:

- Accession codes, unique identifiers, or web links for publicly available datasets
- A description of any restrictions on data availability
- For clinical datasets or third party data, please ensure that the statement adheres to our policy

QTL summary statistics from this study are openly accessible through Zenodo (10.5281/zenodo.20144650). Individual-level data are available from QBB, now part of the Qatar Precision Health Institute (QPHI). Requests for data access should be submitted though the QPHI portal (https://www.qphi.org.qa/research/how-to-apply; Accession ID: QPHI-RES-FO-009) and are subject to review by QPHI Access Committee and QBB IRB. Previously published data used in the study are accessible as follows: ClinVar database v2023-10-01 (https://www.ncbi.nlm.nih.gov/clinvar/), eQTL Catalogue (https://www.ebi.ac.uk/eqtl/Data_access/), eQTLGen (https://www.eqtlgen.org/cis-eqtls.html), GALA II/SAGE (https://zenodo.org/record/7735723), gnomAD v2.1.1 (https://gnomad.broadinstitute.org/), GTEx V8 (https://gtexportal.org/home/downloads/adult-gtex/qtl), GWAS summary statistics (https://www.ebi.ac.uk/gwas/publications/33623009), NCBI UniVec database (www.ncbi.nlm.nih.gov/tools/vecscreen/univec), Qatar Biobank (https://www.qphi.org.qa/), Sardinian QTL (http://eqtlsdownload.irgb.cnr.it/)

Research involving human participants, their data, or biological material

Policy information about studies with human participants or human data. See also policy information about sex, gender (identity/presentation), and sexual orientation and race, ethnicity and racism.

Reporting on sex and gender	Biological sex (male/female) was considered in the analysis.
Reporting on race, ethnicity, or other socially relevant groupings	Study cohort consists of participants from the Qatari population. Admixture and Principal Component Analysis (PCA) was used to delineate the genetic population structure, which is required fro the analysis. No external groupings based on race, ethnicity or social grounds were considered.
Population characteristics	Predominantly healthy Qatari adults (aged 18 or over) recruited from the general population at Qatar Biobank (QBB). 49.3 % of the subjects were females and 50.7% were males.
Recruitment	Subjects were randomly recruited as a population cohort by QBB.
Ethics oversight	All participants signed an informed consent form prior to their participation, and the study was approved by the Institutional Review Board (IRB) at QBB and Sidra Medicine.

Note that full information on the approval of the study protocol must also be provided in the manuscript.

Field-specific reporting

Please select the one below that is the best fit for your research. If you are not sure, read the appropriate sections before making your selection.

☒ Life sciences ☐ Behavioural & social sciences ☐ Ecological, evolutionary & environmental sciences

For a reference copy of the document with all sections, see nature.com/documents/nr-reporting-summary-flat.pdf

Life sciences study design

All studies must disclose on these points even when the disclosure is negative.

Sample size	1,882 subjects for transcriptome sequencing and 6,218 for whole genome sequencing and phenotypic data. These cohorts were provided as the first batch of participants that were recruited at the Qatar Biobank (QBB). The sample sizes are in line with what is needed for QTL and colocalizaiton studies.
Data exclusions	We excluded samples and genetic variants that did not meet standard quality thresholds. Notably, for whole genome sequencing (WGS) data, we excluded variants with non-PASS filter label, missingness level > 1%, MAF < 1% and HWE p-value < 10-6. Also, For the transcriptomics data, we performed QC on the RNA yield, RQN, mapping, coverage, GC content, sample-level matching with WGS, resulting in the exclusion of 146 RNA samples from 2,028 samples that were initially sequenced. For QTL discovery, we excluded results with FDR >0.05. For fine mapping, we excluded those with PIP <0.95 and LBF<2. For col-localisation, we excluded results with PPH4 <0.5 and where variant PIP <0.5.
Replication	We compared our results with various publicly-available datasets. We perform replication as recommended in PEER factors identification using random sets of 1,500 genes.
Randomization	Not applicable as this is a population-based cohort study.
Blinding	Not applicable for this study.

Reporting for specific materials, systems and methods

We require information from authors about some types of materials, experimental systems and methods used in many studies. Here, indicate whether each material, system or method listed is relevant to your study. If you are not sure if a list item applies to your research, read the appropriate section before selecting a response.

Materials & experimental systems

n/a	Involved in the study
☒	☐ Antibodies
☒	☐ Eukaryotic cell lines
☒	☐ Palaeontology and archaeology
☒	☐ Animals and other organisms
☐	☒ Clinical data
☒	☐ Dual use research of concern
☒	☐ Plants

Methods

n/a	Involved in the study
☒	☐ ChIP-seq
☒	☐ Flow cytometry
☒	☐ MRI-based neuroimaging

Clinical data

Policy information about clinical studies

All manuscripts should comply with the ICMJE guidelines for publication of clinical research and a completed CONSORT checklist must be included with all submissions.

Clinical trial registration	NA (No clinical trial)
Study protocol	Sidra IRB protocol 1604003028
Data collection	Clinically-relevant traits and information about age and biological sex for the study participants were collected by QBB. Information was de-identified by QBB prior to utilization in the study.
Outcomes	N/A

Plants

Seed stocks	*Report on the source of all seed stocks or other plant material used. If applicable, state the seed stock centre and catalogue number. If plant specimens were collected from the field, describe the collection location, date and sampling procedures.*
Novel plant genotypes	*Describe the methods by which all novel plant genotypes were produced. This includes those generated by transgenic approaches, gene editing, chemical/radiation-based mutagenesis and hybridization. For transgenic lines, describe the transformation method, the number of independent lines analyzed and the generation upon which experiments were performed. For gene-edited lines, describe the editor used, the endogenous sequence targeted for editing, the targeting guide RNA sequence (if applicable) and how the editor was applied.*
Authentication	*Describe any authentication procedures for each seed stock used or novel genotype generated. Describe any experiments used to assess the effect of a mutation and, where applicable, how potential secondary effects (e.g. second site T-DNA insertions, mosiacism, off-target gene editing) were examined.*
